# Full Structural Characterization of Homoleptic Complexes of Diacetylcurcumin with Mg, Zn, Cu, and Mn: Cisplatin-level Cytotoxicity in Vitro with Minimal Acute Toxicity in Vivo

**DOI:** 10.3390/molecules24081598

**Published:** 2019-04-23

**Authors:** William Meza-Morales, M. Mirian Estévez-Carmona, Yair Alvarez-Ricardo, Marco A. Obregón-Mendoza, Julia Cassani, María Teresa Ramírez-Apan, Carolina Escobedo-Martínez, Manuel Soriano-García, William F. Reynolds, Raúl G. Enríquez

**Affiliations:** 1 Instituto de Química, Universidad Nacional Autónoma de México, Circuito Exterior, Ciudad Universitaria, CDMX CP 07340, México; willy_meza_morales@hotmail.com (W.M.-M.); yfar30@hotmail.com (Y.A.-R.); obregonmendoza@yahoo.com.mx (M.A.O.-M.); mtrapan@yahoo.com.mx (M.T.R.-A.); soriano@unam.mx (M.S.-G.); 2 Escuela Nacional de Ciencias Biológicas, Instituto Politécnico Nacional, Wilfrido Massieu SN, CDMX CP 07738, México; mirianestevezc@gmail.com; 3 Departamento de Sistemas Biológicos, Universidad Autónoma Metropolitana, Unidad Xochimilco, CDMX CP 04960, México; cassani@correo.xoc.uam.mx; 4 Departamento de Farmacia, División de Ciencias Naturales y Exactas, Universidad de Guanajuato, Campus Guanajuato, Guanajuato CP 36050, México; karolesma@hotmail.com; 5 Department of Chemistry, University of Toronto, Toronto, ON M5S 1A1, Canada; wreynold@chem.utoronto.ca

**Keywords:** homoleptic metal complexes, curcuminoids, crystal structures, antioxidant and cytotoxic activity

## Abstract

At the present time, scientists place a great deal of effort worldwide trying to improve the therapeutic potential of metal complexes of curcumin and curcuminoids. Herein, the synthesis of four homoleptic metal complexes with diacetylcurcumin (DAC), using a ligand designed to prevent the interaction of phenolic groups, rendering metal complexes through the β-diketone functionality, is reported. Due to their physiological relevance, we used bivalent magnesium, zinc, copper, and manganese for complexation with DAC. The resulting products were characterized by ultraviolet-visible (UV-Vis), fluorescence spectroscopy, infrared spectroscopy (IR), liquid and solid-state nuclear magnetic resonance (NMR), electron paramagnetic resonance (EPR), magnetic moment, mass spectrometry (MS), single crystal, and powder X-ray diffraction (SCXRD and PXRD). Crystallization was achieved in dimethylsulfoxide (DMSO) or *N,N-*dimethylformamide (DMF) as triclinic systems with space group P-1, showing the metal bound to the β-diketone function, while the ^1^H-NMR confirmed the preference of the enolic form of the ligand. Single crystal data demonstrated a 1:2 metal:ligand ratio. The inhibition of lipid peroxidation was evaluated using the thiobarbituric acid reactive substance assay (TBARS). All four metal complexes (Mg, Zn, Cu, and Mn) exhibited good antioxidant effect (IC_50_ = 2.03 ± 0.27, 1.58 ± 0.07, 1.58 ± 0.15 and 1.24 ± 0.10 μM respectively) compared with butylated hydroxytoluene (BHT) and α-tocopherol. The cytotoxic activity in human cancer cell lines against colon adenocarcinoma (HCT-15), mammary adenocarcinoma (MCF-7), and lung adenocarcinoma (SKLU-1) was found comparable ((DAC)_2_Mg), or ca. 2-fold higher ((DAC)_2_Zn) than cisplatin. The acute toxicity assays indicate class 5 toxicity, according to the Organization for Economic Co-operation and Development (OECD) guidelines at doses of 3 g/kg for all complexes. No mortality or changes in the behavior of animals in any of the treated groups was observed. A therapeutic potential can be envisaged from the relevant cytotoxic activity upon human cancer cell lines in vitro and the undetected in vivo acute toxicity of these compounds.

## 1. Introduction

Curcumin (1,7-*bis*(4-hydroxy-3-methoxyphenol)-1,6-heptadiene-3,5-diketone) is the key secondary metabolite present in the yellow rhizome of the perennial herb, *Curcuma longa*, along with desmethoxycurcumin, and *bis*-desmethoxycurcumin. It has been widely studied for its multiple medicinal properties, e.g., antimicrobial, antioxidant, antibacterial, and anti-inflammatory, including anticancer activity, Alzheimer and infectious diseases [1,2,3,4,5,6,7]. However, the low solubility of curcumin in water, and its rapid metabolism and excretion, are related to a poor bioavailability that limits the scope of its potential benefits in living organisms [1,8,9,10,11,12]. Curcumin and curcuminoids, both natural and synthetic, are examples of β-diketone ligands capable of binding to a variety of metals to produce stable complexes [1,2].

The use of metals of physiological interest in complexation, such as magnesium, zinc, copper, and manganese, serves the purpose of exploring the potential benefits of these metal complexes in medicinal therapies, since the positive biological role of such metals is known to a good extent. For example, magnesium (Mg(II)) is a cofactor in several enzymatic reactions mainly related to energy metabolism and the synthesis of nucleic acids [9,13]. Zinc (Zn(II)) is involved in the regulation of mitochondrial apoptosis in many mammalian cells [14]. Copper (Cu(II)) is an essential micronutrient related to mitochondrial respiration, as well as the response to cellular stress. It is also involved in antioxidant and anti-tumoral defense [10,11,15]. Manganese (Mn(II)) is a trace element present in bone, liver, kidney, and pancreas. Mn(II) helps the body form connective tissue, bones, sex hormones and assists in blood clotting. It also plays a role in fat and carbohydrate metabolism, calcium absorption, and blood sugar regulation. This metal is also necessary for normal brain and nerve function and it is also a component of the antioxidant enzyme superoxide dismutase (SOD), helping to quench free radicals and reduce their damage [16,17].

The molecule curcumin belongs to a class of naturally occurring β-diketones, which can act as good ligand, like its closely related acetylacetone, and also displays the typical keto-enol tautomerism. However, unlike acetylacetone, the central β-diketone functionality is flanked by two large groups –CH=CH-C_6_H_4_(OH)(OMe)-3,4 bearing phenolic –OH groups, which are reactive centers that can interfere in the preparation of metal complexes. Thus, it was necessary to protect the hydroxyl substituents and use diacetylcurcumin (or 1,7-*bis*(3-methoxyl-4-acetoxy1)-pheny-1,6-heptadiene-3,5 diketone (DAC)), which proved to be a suitable ligand [1]. Acetylation turns curcumin into a ligand with its main binding capacities at the β-diketone function.

Wang et al. (2014) reported, for the first time, the crystal structure of two homoleptic curcumin metal complexes, using as ligands the *O*-ethoxy and *O*-butoxy derivatives, through strong alkaline conditions in their synthetic procedure [10]. In other work, Aliaga-Alcalde et al. [18,19] reported the crystal structures of two homoleptic metal complexes with zinc and copper, where the original aromatic rings of curcumin are replaced by anthracene substituents. The scarcity of homoleptic crystal structures reported until now, however, it has been attributed to the low crystallinity of the curcumin-metal association, making the obtention of suitable single crystals difficult [1,10,18,19,20]. By using diacetylcurcumin, we circumvented this barrier, while preserving the overall structure of curcumin in its complexes. The copper and manganese complexes of diacetylcurcumin have been reported previously [8,16], however, the X-ray structures have not been reported. In a recent work by Meza-Morales et al. (2019), the synthesis of five homoleptic complexes, with copper was reported using different curcuminoids [21], along with their single crystal structures.

**Scheme 1:**
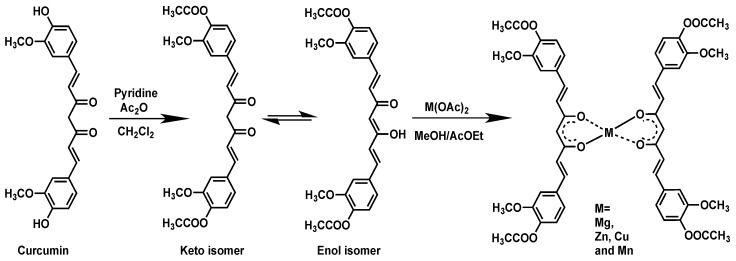
Synthetic route of diacetylcurcumin (DAC) and its DAC-metals complexes.

In the present work, the complexation was conducted under mild conditions to form the Mg(II), Zn(II), Cu(II) and Mn(II) complexes at the β-diketone region, in absence of a strong base. We report the synthesis (see Scheme 1), structural characterization by ultraviolet-visible (UV-Vis), fluorescence, and infrared spectroscopy, liquid and solid-state Nuclear Magnetic Resonance, Electronic Paramagnetic Resonance, magnetic moment and single-crystal X-ray diffraction using diacetylated curcumin as ligand with the physiologically relevant metals Mg, Zn, Cu and Mn. This approach successfully afforded the reportedly elusive homoleptic complexes as single crystals. In addition, these metal complexes of DAC showed enhanced antioxidant and antitumor activity respect to the ligand.

## 2. Results and Discussion

### 2.1. UV-Vis Spectra

The electronic spectra of compounds **1**–**5** were recorded in dimethylsulfoxide (DMSO) at room temperature. The spectra of compounds **1** and **2** exhibit two absorption bands at 408 and 328 nm; 414 and 328 nm. These bands are attributed to π–π* and n–π* transitions. The electronic spectrum of complexes **3**, **4,** and **5** exhibit three bands at 415, 338, and 439; 414 nm, 338 and 438 nm; 418, 328 and 440 nm, due to π–π*, n–π* transitions and charge transfer effects (CT) (see Table 1). The CT bands of compounds **3**, **4,** and **5** demonstrate the formation of the complexes. In addition, very high values of the molar extinction coefficient (ɛ) were found, indicating a high capacity to interact with light. It was not possible to observe the d-d transitions for the complexes **3**, **4,** and **5** [9].

### 2.2. Fluorescence Spectra

All compounds emit at the same wavelength ca. 458 nm, when excited between 408–418 nm (see Table 2). The Mg(II) complex shows an increase in fluorescence intensity, while Zn(II), Cu(II) and Mn(II) complexes show a decrease in intensity compared to the ligand as shown in Table 2. The increase in fluorescence intensity for Mg(II) can be attributed to the charge transfer from metal to ligand [22]. On the contrary, the decrease in intensity for complexes of Zn(II), Cu(II) and Mn(II), can be explained by the charge transfer from the ligand to the metal. The relative intensities for the complexes go in the order, Mg(II) > Cu(II) > Mn(II) > Zn(II) [23].

### 2.3. Infrared Spectra

The IR spectrum of DAC shows the presence of two bands, one of high intensity at 1755 cm^−1^ and the other of very low intensity at 1795 cm^−1^, due to the free carbonyl group of the β-diketone indicating that the compound exists in enolic form. The absorption in the range 1632–1610 cm^−1^ of low intensity corresponds to the intramolecular hydrogen bond of the enol. The -CH=C- band at 965.78 cm^−1^ is also observed. The IR spectra of metal complexes show intense bands for the interaction of the metal with the β-diketonate group at 1447.36, 1509.64, 1514.26 and 1505.53 cm^−1^ corresponding to (DAC)_2_Mg **2**, (DAC)_2_Zn **3**, (DAC)_2_Cu **4** and (DAC)_2_Mn **5**, respectively. All complexes spectra show additional bands at ~466.01, ~469.59, ~484.78, and ~463.93 cm^−1^, due to M-O vibrations [12].

### 2.4. NMR Spectra

The ^1^H nuclear magnetic resonance (NMR) spectrum of ligand DAC shows one singlet for the OH proton, at 16 ppm, and one singlet for the methine proton at ~6.20 ppm; both protons are involved in a strong intramolecular hydrogen bridge (enol tautomer). Protons α to the diketone function appear at 6.99 ppm and protons β at 7.66 ppm, with a trans coupling constant of 15.9 Hz. Methoxyl and acetyl protons appear as singlets at 3.85 ppm, and 2.28 ppm, respectively. The ^1^H-NMR spectra of the complexes lack the corresponding signal of the enol proton at 16 ppm, indicating complex formation through the β-diketonate. The chemical shift towards low frequencies by the protons of the methine, vinyl, and aromatic groups of metal complexes is presumably due to the fact that DMSO is a highly coordinating molecule that could be forming part of the coordination sphere (being part of the complex) [1], increasing the electron density, and shielding effect at the coordination site. The same behavior has been found in the ^1^H-NMR spectra of metal complexes (Cu(II) and Zn(II)) of curcumin [24]. By contrast, the spectra of the metal complexes of curcumin and curcuminoids in MeOD-*d_4_* or CDCl_3_ show the opposite behavior (protons are observed deshielded) [12,25,26]. The ^1^H-NMR spectra of manganese and copper complexes show overall paramagnetic broadening of signals. The methine proton in Zn and Mg complexes, receives the highest shielding effect (Table 3) that can be attributed to an increase in electron density [24]. Methoxyl and acetyl protons are not affected since they are far away from the ligand-metal site of interaction.

### 2.5. NMR Solid State Spectra

**Figure 1:**
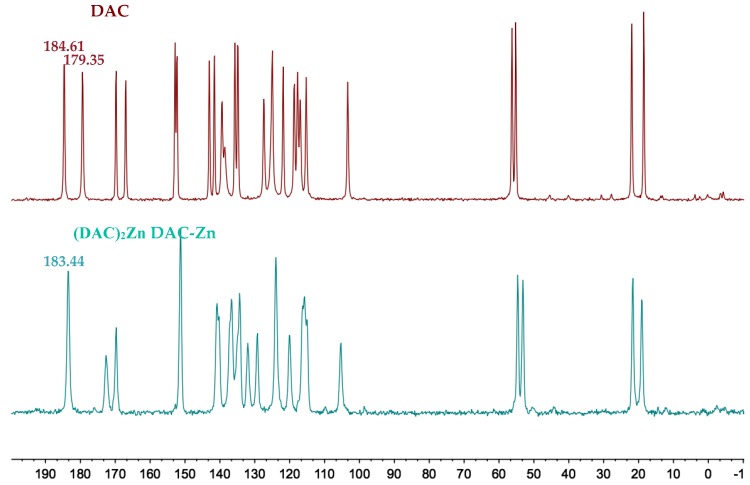
Comparison between 100 MHz ^13^C nuclear magnetic resonance (NMR) spectra of DAC and (DAC)_2_Zn.

The Cross Polarized Magic Angle Spinning (CP-MAS) ^13^C-NMR spectrum of ligand DAC shows signals at 184.61 and 179.35 ppm for the carbonyls of the β-diketone system. The diamagnetic metals complexes (DAC)_2_Mg and (DAC)_2_Zn show the overlap of these signals into a singlet (see Suplementary Information). The CP-MAS spectrum of (DAC)_2_Zn is shown to illustrate this effect (Figure 1). The carbonyls overlap into a singlet at 183.44, which can be attributed to the increased symmetry at the plane of coordination about the metal atom. The CP-MAS spectra of complexes (DAC)_2_Cu and (DAC)_2_Mn prevents such observation, due to the paramagnetic effects.

### 2.6. EPR Spectra

The ligand and their complexes with Mg(II) and Zn(II) were diamagnetic, while the electron paramagnetic resonance (EPR) spectra of Cu(II) and Mn(II) complexes (**4** and **5**) show a typical four and six lines pattern (see Supplementary Material). The g_║_, g_┴_, A_║_ and A_┴_ values were measured from the EPR spectra of the complexes. The g_║_ and g_┴_ values for DAC-Cu were 2.29, and 2.06, respectively (see Table 4); indicating that complexes have unpaired electrons in their d_x2-y2_ molecular orbital. The value of g_║_ is greater than 2.3, suggesting ionic environments in the copper complexes. The A_║_ value is ca. 160 × 10^−4^ cm^−1^ consistent with a typical monomeric distorted square planar structure. The quotient g_║_/A_║_ provides an index of departure from the tetrahedral structure. The quotient value falls in the range of 105–135 cm^−1^ for a regular square planar structure but the observed results (141 cm^−1^) are indicative of strong distortion from planarity. The EPR spectrum of the Mn(II) complex shows one broad isotropic signal with g value of 2.00 (see Table 5). Magnetic moment values of Cu(II) and Mn(II) complexes suggest that there are paramagnetic with µ_effect_ values of 1.7 and 4.8 B.M with one and five unpaired electrons, respectively (see Table 4 and Table 5) [9,27,28,29,30,31].

### 2.7. X-ray Diffraction

Good quality crystals of (DAC)_2_Mg, (DAC)_2_Zn, (DAC)_2_Cu, and (DAC)_2_Mn were obtained by controlled nucleation upon standing. The experimental set up was kept undisturbed for crystallization. Table 6 summarizes the crystal data, collection parameters and refinements for compounds **2**, **3**, **4,** and **5**.

**Figure 2:**
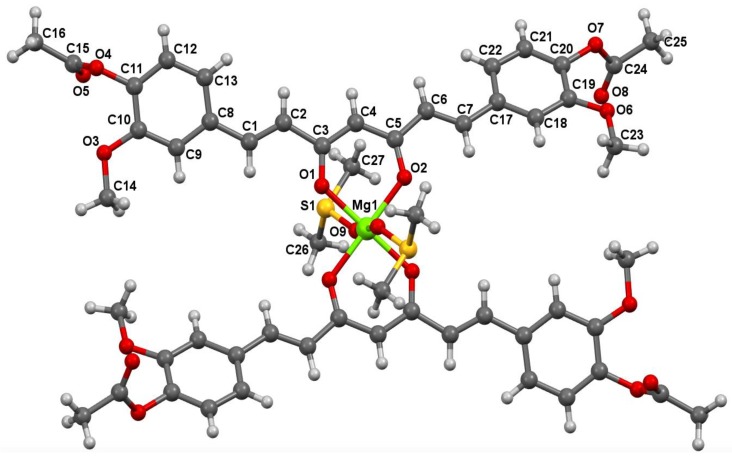
Molecular structure of **2**. Hydrogen atoms are drawn as circles with an arbitrary radius.

**Figure 3:**
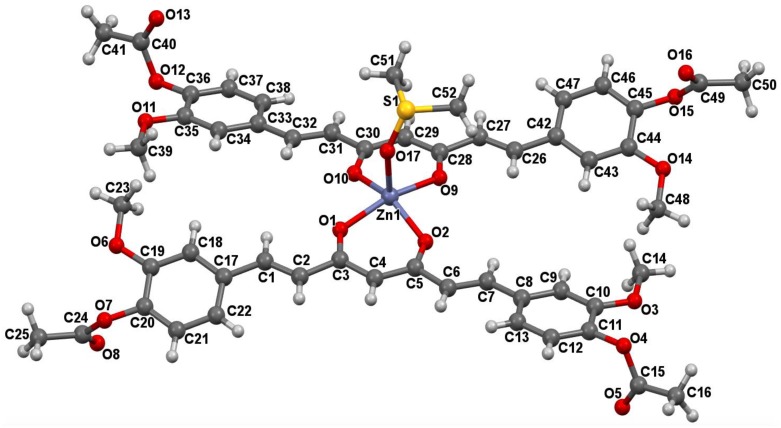
Molecular structure of **3**. Hydrogen atoms are drawn as circles with an arbitrary radius.

**Figure 4:**
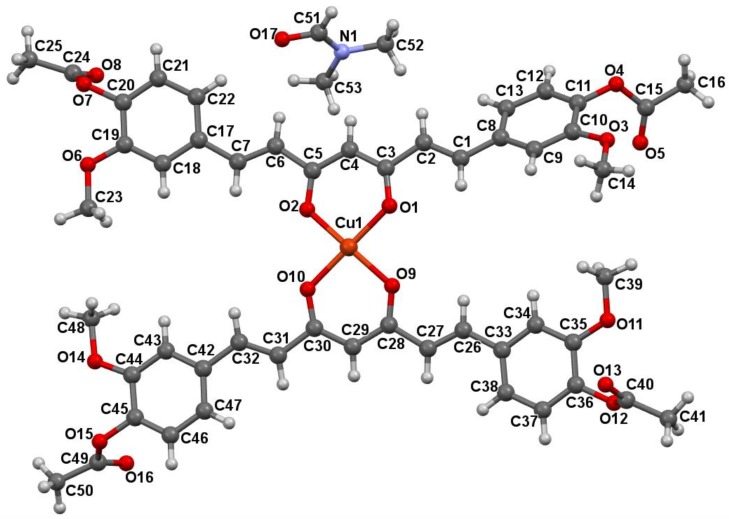
Molecular structure of **4**. Hydrogen atoms are drawn as circles with an arbitrary radius.

**Figure 5:**
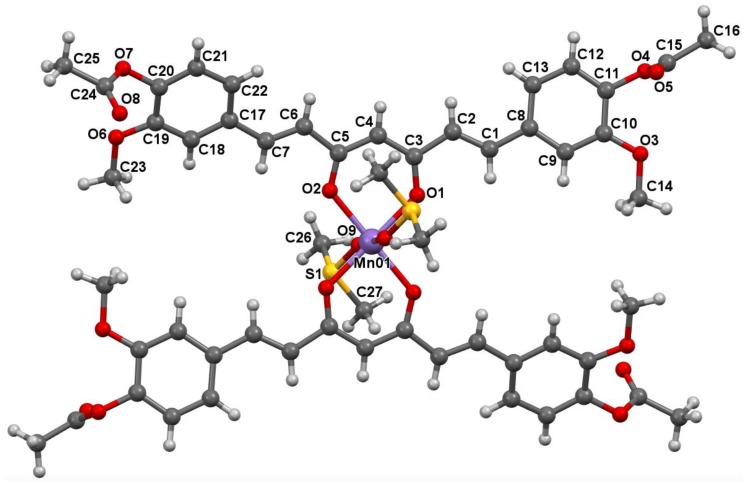
Molecular structure of **5**. Hydrogen atoms are drawn as circles with an arbitrary radius.

The molecular structures of compounds **2**, **3**, **4,** and **5** are shown in Figure 2, Figure 3, Figure 4 and Figure 5 with displacement ellipsoids at 50% probability level.

Selected bond distances and bond angles for compounds **2**, **3**, **4,** and **5** are listed in Table S1.

The values of the M-O bond distances of the metal complexes DAC and the corresponding metal acetylacetonates are shown in the Table 7, showing great similarity among them [32,33,34,35,36,37,38].

The new solid phases of metal complexes were confirmed by powder X-ray diffraction (PXRD), as can be appreciated from strong differences in the PXRD pattern of magnesium, zinc, copper, and manganese complexes compared to DAC (see Supplementary Material).

### 2.8. Inhibition of Lipid Peroxidation (LP) in Rat Brain Homogenate

The four metal complexes of DAC showed high antioxidant activity on the lipid peroxidation in rat brain homogenate model (see Table 8). The IC_50_ values of complexes **2**–**5** show higher antioxidant activity than α−tocopherol and comparable to that of BHT. According to previous works, the antioxidant effects of curcumin are retained or enhanced in its metal complex form [39,40].

### 2.9. Cytotoxic Activity

The results indicate that complexes **2**, **3** and **5** (Mg, Zn, and Mn, respectively) had important cytotoxic effects against the cell lines HCT-15, MCF-7, and SKLU-1, the IC_50_ values of three homoleptic complexes being similar or lower than cisplatin, and much lower than compound **1** (Table 9). These results agree with several studies of antitumoral activity with heteroleptic metal complexes of curcumin, where various authors suggest that the metal presence in the complex preserves the therapeutic potential of curcumin or increases its stability in cellular medium [1,39,41]. Moreover, the importance and application of transition metals in tumor-selective complexes, as drug-delivery agents, has been amply described previously [9,41]. Interestingly, in this work, compound **4** (copper) did not show a cytotoxic effect against the cell lines tested. Although there are several reports of antitumoral activity of copper complexes with curcumin, the cytotoxic effects seem to be related to the presence of free phenolic groups [8,15,42].

### 2.10. Acute Toxicity Study in Mice

There was no mortality of mice in any of the groups up to a dose of 3 g kg^−1^ (see Table 10). Only the oral administration of the (DAC)_2_Cu and (DAC)_2_Mn complexes provoked minor bristling of the fur (see Table 11). The body weight of all mice gradually increased and was not significantly different to the control groups, which started on the first day of treatment.

## 3. Materials and Methods

All chemicals were available commercially and the solvents were purified with conventional methods prior to use [43]. Curcumin was obtained from natural source by usual extractive procedures and purified by crystallization.

### 3.1. Physical Measurements

Melting points were determined on an Electrothermal Engineering IA9100X1 melting point apparatus and are uncorrected.

### 3.2. Spectroscopic Determinations

UV-Vis spectra were recorded in a UV-Visible Spectrophotometer Varian Cary 400 (Varian NMR Instruments Palo Alto, California, CA, USA; www.agilent.com). The fluorescence measurements of the samples were recorded on the Varian-Cary-Eclipse equipment (Varian NMR Instruments Palo Alto, California, CA, USA; www.agilent.com). IR absorption spectra were recorded in the range of 4000–230 cm^−1^ as KBr pellets on a BRUKER Tensor 27 spectrophotometer. ^1^H- and ^13^C-NMR spectra were recorded in CDCl_3_ and DMSO-*d*_6_ on a JEOL 300 MHz and Unity Varian 500 MHz and an Agilent One Probe 600 MHz spectrometers using TMS as an internal reference. Solid State CP-MAS NMR spectra were recorded to monitor complexation and data are included in the Supplementary Material. The EPR spectra were recorded in DMSO and DMF at liquid nitrogen temperature (77 K) on an Electronic Paramagnetic Resonance Spectrometer Jeol, JES-TE300, ITC Cryogenic System, Oxford. The magnetic moment of the compounds was determined using a Johnson-Matthey magnetic susceptibility balance type msb model mk II 13094-3002 (Thermo Fisher Scientific, Applied Biosystems de México, S. de R.L. México, CDMX), with the Gouy method at room temperature. High resolution mass spectra for complexes of (DAC)_2_Zn (**3**) and (DAC)_2_Cu (**4**) were recorded in a MStation JMS-700 equipment (JEOL Mexico S.A of C.V., México, CDMX) in the fast atom bombardment (FAB+) mode and low resolution mass spectra for (DAC)_2_Mg (**2**) and (DAC)_2_Mn (**5**) were recorded in a JEOL, SX 102. A spectrometer on Bruker Microflex equipped with MALDI-Flight time. Elemental analysis were performed in a Thermo Scientific elemental analyzer (ThermoFisher Scientific, Applied Biosystems de México, S. de R.L. de C.V., México, CDMX), model Flash 2000 and a Perkin Elmer elemental analyzer, model 2400CHNSO. Single-crystal X-ray diffraction (SCXRD) were determined in a Bruker diffractometer (Bruker Mexicana S.A. de C. V., Mexico, CDMX), model Smart Apex, equipped with Mo radiation (λ = 0.71073Å), CCD two-dimensional detector and a lw temperature device. Data collection and data reduction were performed by APEX and SAINT-Plus programs (Bruker-Nonius AXS Inc., Madison, WI, USA), [44]. The structures were solved by the direct method with the structure solution program-SHELXS-97 and Refinement program-SHELXL97 (Dept. of Structural Chemistry, Universität Göttingen, Göttingen, Germany) and refined by full-matrix least-squares procedure on F^2^ using SHELX-2008 program [45]. Powder X-ray diffraction (PXRD) were determined in a Bruker diffractometer, model D8 Advance Davinci, theta-theta configuration Bruker AXS, Lynxeye detector.

### 3.3. Inhibition of Lipid Peroxidation in Rat Brain

#### 3.3.1. Animals

Adult male Wistar rats (200–250 g) were provided by the Instituto de Fisiología Celular, Universidad Nacional Autónoma de México (UNAM). Procedures and care of animals were conducted in conformity with the Mexican Official Norm for Animal Care and Handling NOM-062-ZOO-1999. They were maintained at 23 ± 2 °C on a 12/12 h light-dark cycle with free access to food and water.

#### 3.3.2. Rat Brain Homogenate Preparation

Animal sacrifice was carried out avoiding unnecessary pain. Rats were sacrificed with CO_2_. The cerebral tissue (whole brain), was rapidly dissected and homogenized in phosphate-buffered saline (PBS) solution (0.2 g of KCl, 0.2 g of KH_2_PO_4_, 8 g of NaCl, and 2.16 g of NaHPO_4_∙7H_2_O/L, pH adjusted to 7.4), as described elsewhere to produce a 1/10 (*w*/*v*) homogenate [46,47]. The homogenate was then centrifuged for 10 min at 800 rcf (relative centrifugal field) to yield a pellet that was discarded. The supernatant protein content was measured using Folin and Ciocalteu’s phenol reagent [48] and adjusted with PBS at 2.666 mg of protein/mL.

#### 3.3.3. Induction of Lipid Peroxidation and Thiobarbituric Acid Reactive Substances (TBARS) Quantification

As an index of lipid peroxidation, TBARS levels were measured using rat brain homogenates according to the method described by Ng and co-workers [49], with some modifications. Supernatant (375 μL) was added with 50 μL of 20 μM EDTA and 50 μL of each sample concentration dissolved in DMSO (50 μL of DMSO for control group) and incubated at 37 °C for 30 min. Lipid peroxidation was started adding 50 μL of freshly prepared 100 μM FeSO_4_ solution (final concentration 10 μM), and incubated at 37 °C for 1 h. The TBARS content was determined, as described by Ohkawa and co-workers [50], with some modifications. A volume of 500 μL of TBA reagent (1% 2-thiobarbituric acid in 0.05 N NaOH and 30% trichloroacetic acid, in 1:1 proportion) was added to each tube and the final suspension was cooled on ice for 10 min, centrifuged at 13,400 rcf for 5 min and heated at 80 °C in a water bath for 30 min. After cooling at room temperature, the absorbance of 200 μL of supernatant was measured at λ = 540 nm in a UltraMicroplate (Synergy/HT BIOTEK Instrument Inc., Winooski, VT, USA). The concentration of TBARS was calculated by interpolation on a standard curve of tetra-methoxypropane (TMP) as a precursor of MDA [51]. Results are expressed as nmoles of TBARS per mg of protein. The inhibition ratio (IR [%]) was calculated using the formula IR = (C – E) × 100/C, where C is the control absorbance and E is the sample absorbance. Butylated hydroxytoluene (BHT) and α-tocopherol were used as positive standards. All data were represented as mean ± standard error (SEM). Data were analyzed by one-way analysis of variance (ANOVA) followed by Dunnett’s test for comparison against control. Values of *p* ≤ 0.05 (*) and *p* ≤ 0.01 (**) were considered statistically significant.

### 3.4. Citotoxic Activity in Human Tumor Cells

Cytotoxicity of all compounds was tested against three cancer cell lines: HCT-15 (human colon adenocarcinoma), MCF-7 (human mammary adenocarcinoma) and SKLU-1 (human lung adenocarcinoma). Cell lines were supplied by the U.S. National Cancer Institute (NCI). The cell lines were cultured in RPMI-1640 medium, supplemented with 10% fetal bovine serum, 2 mM l-glutamine, 10,000 units/mL penicillin G sodium, 10,000 μg/mL streptomycin sulfate, 25 μg/mL amphotericin B (Invitrogen/Gibco™, Thermo Fisher Scientific, Waltham, MA, USA) and 1% non-essential amino acids (Gibco). They were maintained at 37 °C in a humidified atmosphere with 5% CO_2_.The viability of the cells used in the experiments exceeded 95% as determined with trypan blue. The human tumor cytotoxicity was determined using the protein-binding dye sulforhodamine B (SRB) in microculture assay to measure cell growth, as described in the protocols established by the NCI [52,53,54]. The results were expressed as inhibitory concentration 50 (IC_50_) values. They were calculated according to the protocol of Monks [52], where a dose–response curve was plotted for each compound and the concentration (IC_50_), resulting in an inhibition of 50% estimated through non-linear regression analysis.

### 3.5. Acute Toxicity Study In Mice

The experiments were performed using adult male CD1 mice (body-weight range, 25–30 g). Food was withheld for 12 h prior to experiment with animals having free access to drinking water. The concentration of the tested compounds (DAC, (DAC)_2_Mg, (DAC)_2_Zn, (DAC)_2_Cu and (DAC)_2_Mn) was adjusted for oral administration of 1000 and 3000 mg/kg (three animals per dose) according to the Lorke method [55]. The mice were observed daily for a period of 14 days for mortality, toxic effects, and/or changes in weight and behavioral patterns.

All experiments were performed in accordance with ethical standards for experimental pain research in animals [56] and the Mexican Official Standard for animal care and handling (NOM-062-ZOO-1999). The experimental protocol (CIBIUG-P15-2018) was approved and overseen by the Institutional Ethics Committee for Care and Use of Laboratory Animals of the Universidad de Guanajuato, and the CD1 mice were obtained from the Natural and Exact Science Vivarium at Universidad de Guanajuato, under controlled temperature (23 ± 2 °C) and humidity (55 ± 10%), with a 12-h light/darkness cycle, and were familiarized with the environment one week before the experiments. The animals were allowed food and water ad libitum. Immediately after the experiments, all animals were sacrificed in a CO_2_ chamber.

### 3.6. Synthesis of Compounds

General synthesis procedure of DAC **1**, DAC-Magnesium **2**, DAC-Zinc **3**, DAC-Copper **4** and DAC-Manganese **5** are shown in Scheme 1.

DAC (**1**). DAC was obtained in accordance to a synthetic method reported [57]. 70.1% yield. ^1^H-NMR (300 MHz CDCl_3_): δ 2.32 (s, 6H), 3.87 (s, 6H), 5.85 (s, 1H), 6.56 (d, 2H, *J* 16.2 C_vinyl_H), 7.11 (m, 6H, C_aryl_H), 7.61 (d, 2H, *J* 16.2 C_vinyl_H), 15.85 (br s, 1H,), ^13^C-NMR (^13^C {^1^H} 75 MHz, CDCl_3_): δ 20.74 (C-H), 56.01 (C-H), 101.88 (C-H), 111.56 (C_aryl_H), 121.16 (C_aryl_H), 123.39 (C_aryl_H), 124.36 (C_vinyl_H), 134.06 (C_aryl_H), 140.04 (C_vinyl_H), 141.41 (C_aryl_), 151.51(C_aryl_), 168.88 (C=O), 183.19 (C=O), ^1^H-NMR (600 MHz DMSO-*d*_6_): δ 2.28 (s, 6H), 3.85 (s, 6H), 6.20 (s, 1H), 6.99 (d, 2H, *J* 15.9 C_vinyl_H), 7.16 (d, 2H, *J* 8.1 C_aryl_H), 7.33 (dd, 2H, *J* 8.2; 1.9 C_aryl_H), 7.52 (d, 2H, *J* 2 C_aryl_H), 7.66 (d, 2H, *J* 15.9 C_vinyl_H), 16.1 (br s, 1H,), ^13^C-NMR (^13^C {^1^H} 150 MHz, DMSO-*d*_6_): δ 20.17 (C-H), 55.72 (C-H), 101.62 (C-H), 111.93 (C_aryl_H), 121.27 (C_aryl_H), 123.23 (C_aryl_H), 124.58 (C_vinyl_H), 133.59 (C_aryl_H), 139.77 (C_vinyl_H), 140.93 (C_aryl_), 151.11(C_aryl_), 168.30 (C=O), 183.10 (C=O), IR (1755.84 cm^−1^, 1596.49 cm^−1^, 1506.17 cm^−1^, 1295.51 cm^−1^, 1154.04 cm^−1^, 619.80 cm^−1^), HRMS: M^+^ 453.1541 (calculated exact mass 453.1549), yellow crystals m.p.: 170.5 °C. Elemental Analysis: Calculated for C_25_H_24_O_8_: C 66.36, H 5.35; experimental: C 66.27; H 5.33.

DAC-Magnesium (**2**). 1 mmol of DAC was dissolved in a mixture of 25 mL ethyl acetate-methanol (7:3), and later a solution of magnesium acetate in methanol (0.5 mmol) was added slowly. After 2 h of stirring at room temperature, a yellow powder was formed in the flask (m.p. 245.6 °C), which was filtered and crystallized with DMSO (m.p. 239.0 °C), 83.1% yield. ^1^H-NMR (600 MHz DMSO-*d*_6_): δ 2.26 (s, 6H), 3.83 (s, 6H), 5.71 (s, 1H), 6.85 (d, 2H, *J* 15.6 C_vinyl_H), 7.09 (d, 2H, *J* 8.1 C_aryl_H), 7.20 (dd, 2H, *J* 8.1; 1.8 C_aryl_H), 7.41 (d, 2H, *J* 1.9 C_aryl_H), 7.41 (d, 2H, *J* 15.6 C_vinyl_H), ^13^C-NMR (^13^C {^1^H} 150 MHz, DMSO-*d*_6_): δ 20.32 (C-H), 55.63 (C-H), 103.33 (C-H), 111.81 (C_aryl_H), 120.31 (C_aryl_H), 123.01 (C_aryl_H), 131.08 (C_vinyl_H), 134.58 (C_aryl_H), 135.18 (C_vinyl_H), 139.67 (C_aryl_), 150.91 (C_aryl_), 168.04 (C=O), 180.96 (C=O)), IR (3263.94 cm^−1^, 1754.19 cm^−1^, 1595.53 cm^−1^, 1509.41 cm^−1^, 1457.34 cm^−1^, 1204.07 cm^−1^, 1119.45 cm^−1^, 630.36 cm^−1^) LRMS: M^+^ 927.347. (calculated exact mass 927.2709). Elemental Analysis: Calculated for C_50_H_46_MgO_16_·2H_2_O: C 62.35, H 5.23; experimental: C 62.54; H 5.42.

DAC-Zinc (**3**). 1 mmol of DAC was dissolved in a mixture of 25 mL ethyl acetate-methanol (7:3), and later, a solution of zinc acetate in methanol (0.5 mmol) was added slowly. After 2 h of stirring at room temperature, a yellow powder was formed in the flask (m.p. 247.7 °C), which was filtered and crystallized with DMSO (m.p of crystal: 258.9 °C), 95.6% yield. ^1^H-NMR (600 MHz DMSO-*d*_6_): δ 2.26 (s, 6H), 3.83 (s, 6H), 5.84 (s, 1H), 6.91 (d, 2H, *J* 15.7 C_vinyl_H), 7.11 (d, 2H, *J* 8.1 C_aryl_H), 7.26 (dd, 2H, *J* 8.3; 1.8 C_aryl_H), 7.47 (d, 2H, *J* 1.9 C_aryl_H), 7.53 (d, 2H, *J* 15.6 C_vinyl_H), ^13^C-NMR (^13^C {^1^H} 150 MHz, DMSO-*d_6_*): δ 20.24 (C-H), 55.74 (C-H), 102.92 (C-H), 111.40 (C_aryl_H), 120.58 (C_aryl_H), 123.11 (C_aryl_H), 129.89 (C_vinyl_H), 134.20 (C_aryl_H), 137.49 (C_vinyl_H), 140.07 (C_aryl_), 150.90 (C_aryl_), 168.83 (C=O), 183.89 (C=O)), IR (3325.95 cm^−1^, 1757.91 cm^−1^, 1587.25 cm^−1^, 1508.59 cm^−1^, 1444.73 cm^−1^, 1203.53 cm^−1^, 1119.43 cm^−1^, 631.93 cm^−1^), HRMS: M^+^ 967.2168 (calculated exact mass 967.2156). Elemental Analysis: Calculated for C_50_H_46_O_16_Zn·H_2_O: C 60.89, H 4.91; experimental: C 60.23; H 5.09.

DAC-Copper (**4**). 1 mmol of DAC was dissolved in a mixture of 25 mL ethyl acetate-methanol (7:3), and later, a solution of copper acetate in methanol and water (0.5 mmol) was added slowly. After 2 h of stirring at room temperature, a brown powder was formed in the flask (m.p. 242.5 °C), which was filtered and crystallized with DMF (m.p 240 °C.), 86.9% yield. ^1^H-NMR (500 MHz DMSO-*d*_6_): δ 2.27 (s, 6H), 3.85 (s, 6H), 6.26 (br s, 1H), 6.81 (br s, 4H), 7.03 (br s, 1H), 7.16 (br s, 1H), 7.34 (br s, 2H), 7.52 (br s, 1H), 7.66 (br s, 1H), ^13^C-NMR (^13^C {^1^H} 125 MHz, DMSO-*d*_6_): δ 20.37 (C-H), 55.89 (C-H), 101.59 (C-H), 112.29 (C_aryl_H), 121.51 (C_aryl_H), 123.21 (C_aryl_H), 124.72 (C_vinyl_H), 133.57 (C_aryl_H), 139.99 (C_vinyl_H), 140.87 (C_aryl_), 151.13 (C_aryl_), 168.48 (C=O), 183.31 (C=O), IR (2975.04 cm^−1^, 2941.34 cm^−1^, 1752.55 cm^−1^, 1592.19 cm^−1^, 1514.26 cm^−1^, 1412.95 cm^−1^, 1299.11 cm^−1^, 1156.63 cm^−1^, 604.42 cm^−1^), HRMS: M^+^ 966.2164 (calculated exact mass 966.2160). Elemental Analysis: Calculated for C_50_H_46_CuO_16_·H_2_O: C 61.00, H 4.91; experimental: C 60.96; H 5.04.

DAC-Manganese (**5**). 1 mmol of DAC was dissolved in a mixture of 25 mL ethyl acetate-methanol (7:3), and later, a solution of manganese acetate in methanol (0.5 mmol) was added slowly. After 2 h of stirring at room temperature, a brown powder was formed in the flask (m.p.191.5 °C), which was filtered and crystallized from DMSO (m.p. 201.3°), 85.9% yield. ^1^H-NMR (500 MHz DMSO-*d*_6_): δ 2.27 (br, 6H), 3.76 (br, 6H), 5.32 (br s, 1H), 7.98 (br s, 2H), IR (3264.94, 1752.62, 1629.42, 1592.61, 1505.53, 1203.53, 1155.12, 602.92, 463.93 cm^−1^), LRMS: M^+^ 958.167. (calculated exact mass 958.2239). Elemental Analysis: Calculated for C_50_H_46_MnO_16_·H_2_O: C 61.54, H 4.96; experimental: C 61.42; H 4.79.

## 4. Conclusions

Four homoleptic complexes of Mg(II), Zn(II), Cu(II), and Mn(II), with DAC, were synthesized and characterized by physicochemical means as amorphous powders and single crystals. All complexes crystallized in the P-1 space group show that metals are linked to the β-diketone functionality of DAC in its enolic form. While the ligand design preserving the curcumin *motif* had the choices of etherification or esterification, it was acetylation that proved to be synthetically advantageous. The NMR spectra in solid state (CP-MAS) of non-paramagnetic amorphous complexes, allowed the assessment of complexation through the maximum change in the chemical shifts of carbonyls at the β-diketone function once the 1:2 metal:ligand ratio is reached. It was found also that the use of DMSO and DMF as solvents for crystallization was important for the formation of single crystals. The approach reported here provides an accesible route for the synthesis of homoleptic metal complexes of DAC overcoming the fact that curcumin and its derivatives have been described as elusive in the formation of single crystals. From a pharmacological point of view, it can be considered more convenient to handle a homoleptic complex composed exclusively of two curcuminoid moieties rather than a heteroleptic one (stabilizer, curcuminoid and metal ion).

The four homoleptic complexes studied inhibited lipid peroxidation in the rat brain tissue model with higher activity than α-tocopherol and comparable to BHT. It is noteworthy that all copper complexes synthesized in our previous report [21] as well as the copper complex in the present one, exhibited considerable antioxidant effect but negligible cytotoxicity. Of special significance is the remarkable cytotoxic effect found for (DAC)_2_Mg and (DAC)_2_Zn against HCT-15, MCF-7 and SKLU-1 human cancer cell lines, which surprisingly equated or surpassed that of cisplatin. Moreover, the preliminary in vivo experiments showed that at a dose of 3 g/kg the complexes fall within category 5 (2000–5000 mg/kg, i.e., the less toxic category for chemical compounds, according to the OECD protocols). Considering the powerful antioxidant effects and the significant cytotoxicity produced by the metal complexes of DAC reported here upon three human cancer cell lines, together with their minimal in vivo acute toxicity found, further research can be expected to provide a deeper insight into this findings.

## Figures and Tables

**Table 1:** Transition bands in ultraviolet-visible (UV-Vis) spectra for ligand and complexes.

Ligand & Complexes	π–π* (nm)	ɛ_max_ (L/cm mol)	n–π* (nm)	ɛ _max_ (L/cm mol)	CT (nm)	ɛ _max_ (L/cm mol)
DAC (**1**)	408	349,187.06	328	34,955.35	-	-
(DAC)_2_Mg (**2**)	414	587,658.78	328	137,652.06	-	-
(DAC)_2_Zn (**3**)	415	250,821.69	338	211,677.96	439	161,111.68
(DAC)_2_Cu (**4**)	414	387,091.23	338	128,582.44	438	266,119.18
(DAC)_2_Mn (**5**)	418	300,551.07	328	111,206.39	440	213,207.90

**Table 2:** Fluorescence of diacetylcurcumin (DAC) and metal complexes in dimethylsulfoxide (DMSO).

Ligand & Complexes	Excitation Wavelength (nm)	Fluorescence Wavelength (nm)	Intensity (I)
DAC (**1**)	408	457.77	280,465.66
(DAC)_2_Mg (**2**)	414	458.64	412,828.49
(DAC)_2_Zn (**3**)	415	459.06	175,399.40
(DAC)_2_Cu (**4**)	414	458.50	230,331.13
(DAC)_2_Mn (**5**)	418	459.08	188,354.49

**Table 3:** ^1^H-NMR chemical shifts of the methine proton of DAC and their metal complexes.

Ligand & Complexes	δ (ppm) of Methine in DMSO-*d*_6_
DAC (**1**)	6.20
(DAC)_2_Mg (**2**)	5.71
(DAC)_2_Zn (**3**)	5.84
(DAC)_2_Cu (**4**)	(Not observed)
(DAC)_2_Mn (**5**)	5.32

**Table 4:** Electron paramagnetic resonance (EPR) spectral data of complex (DAC)_2_Cu in DMF.

Complex	*g*_║_	*g*_┴_	A_║_ (10^−4^ cm^−1^)	A_┴_ (10^−4^ cm^−1^)	*g*_║_/A_║_ (cm^−1^)	µ_effect_
(DAC)_2_Cu (**4**)	2.29	2.06	162.3	1.13	141.1	1.7

**Table 5:** EPR spectral data of complex (DAC)_2_Mn in DMSO.

Complex	*g*	A (10^−4^ cm^−1^)	*g*/A (cm^−1^)	µ_effect_
(DAC)_2_Mn (**5**)	2.00	85	235.3	6.2

**Table 6:** Crystal Data, Collection Parameters and Refinements for compounds **2, 3, 4,** and **5**.

Compound	2	3	4	5
CCDC deposit No.	1,418,638	1,453,160	1,506,152	1,534,704
Crystal size (mm)	0.23 × 0.05 × 0.04	0.20 × 0.18 × 0.04	0.10 × 0.04 × 0.040	0.29 × 0.11 × 0.03
Color/shape	Colorless/prism	Yellow/prism	Yellow/prism	Colorless/prism
Empirical formula	C_54_H_58_ Mg O_18_S_2_	C_52_H_52_ Zn O_17_S	C_53_H_53_CuNO_17_	C_54_H_58_MnO_18_S_2_
Formula weight	1083.43	1046.36	1039.50	1114.06
Crystal system	Triclinic	Triclinic	Triclinic	Triclinic
Space group	*P*-1	*P*-1	*P*-1	*P*-1
Unit cell dimensions				
*a*, Å	7.4419(5)	7.4706(2)	12.7683(7)	7.513(5)
*b*, Å	12.1480(7)	11.7637(4)	13.2372(7)	12.165(14)
*c*, Å	15.8082(10)	30.7699(10)	15.3255(8)	15.688(17)
*α*, deg	80.701(2)	81.6770(10)	99.5684(18)	80.87(10)
*β*, deg	85.653(2)	86.8260(10)	103.0083(19)	85.68(6)
γ, deg	81.390(2)	79.6230(10)	94.303(2)	80.40(7)
Volume, Å^3^	1392.50(15)	2630.68(14)	2471.6(2)	1394(2)
*Z*	1	2	2	1
Density (calculated), Mg /m^3^	1.292	1.321	1.397	1.327
Absorption coefficient, mm^−1^	0.177	0.576	0.517	0.382
*F* (000)	570	1092	1086	583
*Θ* range for data collection (°)	2.31 to 25.37	2.07 to 25.36	2.41 to 25.37	2.31 to 25.40
Index ranges	−8 ≤ η ≤ 8, −14 ≤ κ ≤ 14, −19 ≤ λ ≤ 18	−8 ≤ η ≤ 8, −14 ≤ κ ≤ 14, −36 ≤ λ ≤ 37	−15 ≤ η ≤ 15, −15 ≤ κ ≤ 15, −18 ≤ λ ≤ 16	−8 ≤ η ≤ 9, −14 ≤ κ ≤ 14, −18 ≤ λ ≤ 18
Reflections collected	20,701	43,039	40,595	22,968
Independent reflections	5086 (*R*int = 0.1991)	9550 (*R*int = 0.0736)	9019 (*R*int = 0.16875)	5086 (*R*int = 0.1893)
Data/restraints/parameters	5086/0/346	9550/90/676	9019/0/659	5086/20/357
Goodness-of-fit on F^2^	0.859	1.054	1.096	1.034
Final *R* índices [(*Ι*) > 2σ (*Ι*)]	*R1 =* 0.0676, w*R2 =* 0.1330	*R1 =* 0.0685, w*R2 =* 0.1517	*R1 =* 0.0889, w*R2 =* 0.1580	*R1 =* 0.0768, w*R2 =* 0.1538
*R* indices (all data)	*R1 =* 0.1798, *wR2 =* 0.1812	*R1 =* 0.1073, *wR2 =* 0.1714	*R1 =* 0.1644, *wR2 =* 0.1949	*R1 =* 0.1571, *wR2 =* 0.1939
Largest diff. peak and hole (ε/A^−3^)	0.300 and −0.430	1.354 and −0.495	0.539 and −0.606	0.337 and −0.373

**Table 7:** Comparison of bond distances (Å) of metal complexes of diacetylcurcumin and acetylacetonates.

Compounds	M-O (Å)	Melting Point	Geometry
(DAC)_2_Mg (**2**)	Mg(1)-O(1) 2.022 Mg(1)-O(2) 2.053	245.6 °C	octahedral
acac-Mg	Mg(1)-O(1) 2.040 Mg(1)-O(2) 2.027	265 °C	octahedral
(DAC)_2_Zn (**3**)	Zn(1)-O(1) 2.021 Zn(1)-O(2) 1.987 Zn(1)-O(9) 2.031 Zn(1)-O(10) 1.991	247.7 °C	trigonal bipyramid
acac-Zn	Zn(1)-O(11) 2.002 Zn(1)-O(12) 1.971 Zn(1)-O(21) 2.008 Zn(1)-O(22) 2.096	136.5 °C	trigonal bipyramid
(DAC)_2_Cu (**4**)	Cu(1)-O(1) 1.906 Cu(1)-O(2) 1.900 Cu(1)-O(9) 1.916 Cu(1)-O(10) 1.913	242.5 °C	square planar
acac-Cu	Cu(1)-O(1) 1.914 Cu(1)-O(2) 1.912	285.5 °C	square planar
(DAC)_2_Mn (**5**)	Mn(1)-O(1) 2.108 Mn(1)-O(2) 2.132	191 °C	octahedral
acac-Mn	Mn(1)-O(1) 2.150 Mn(1)-O(2) 2.129	249 °C	octahedral

**Table 8:** IC_50_ of inhibition of lipid peroxidation of compounds **1**, **2**, **3**, **4,** and **5**.

Compounds	Concentration (μM)	TBARS (nmol/mg Protein)	% Inhibition	IC_50_ (μM)
Basal	-	0.19 ± 0.03	-	-
Control FeSO_4_	-	10.11 ± 0.56	-	-
α−Tocoferol (*n* = 4)	Basal Control 0.1 0.32 1 3.16 10 31.62 100	0.200 ± 0.011 6.589 ± 0.213 6.283 ± 0.188 6.048 ± 0.242 5.211 ± 0.332 * 3.676 ± 0.569 ** 2.725 ± 0.335 ** 1.849 ± 0.319 ** 1.408 ± 0.364 **	- - 4.62 ± 0.57 8.26 ± 1.31 21.13 ± 2.56 * 44.84 ± 6.74 ** 59.00 ± 3.71 ** 72.3 ± 3.87 ** 79.09 ± 4.79 **	6.78 ± 2.16
BHT (*n* = 5)	Basal Control 0.32 0.42 0.56 0.75 1 1.33 1.78 2.37 3.16	0.268 ± 0.053 7.384 ± 0.630 6.690 ± 0.655 6.510 ± 0.551 6.098 ± 0.353 5.559 ± 0.294 * 4.457 ± 0.283 ** 3.228 ± 0.572 ** 1.315 ± 0.489 ** 0.487 ± 0.075 ** 0.53 ± 0.044 **	- - 9.53 ± 2.91 11.62 ± 2.78 16.64 ± 2.86 23.92 ± 2.69 * 37.14 ± 7.44 ** 53.59 ± 8.93 ** 81.59 ± 6.89 ** 93.16 ± 1.16 ** 95.08 ± 0.68 **	1.22 ± 0.44
DAC (**1**)	0.1 0.32 1 3.16 10	9.68 ± 0.43 9.29 ± 0.67 7.78 ± 0.53 * 5.22 ± 0.35 ** 1.43 ± 0.08 **	4.16 ± 1.97 8.32 ± 1.49 23.19 ± 0.92 * 48.46 ± 1.53 ** 85.82 ± 0.60 **	3.21 ± 0.16
(DAC)_2_Mg (**2**)	0.1 0.33 1 3.28 10.38	9.50 ± 0.63 8.48 ± 0.78 6.55 ± 0.67 ** 3.56 ± 0.37 ** 0.34 ± 0.02 **	6.20 ± 1.00 16.41 ± 3.31 35.52 ± 3.78 ** 64.52 ± 4.42 ** 96.63 ± 0.08 **	2.03 ± 0.27
(DAC)_2_Zn (**3**)	0.1 0.32 1 3.22 10.20	9.16 ± 0.90 8.02 ± 0.72 6.22 ± 0.67 ** 2.87 ± 0.14 ** 0.35 ± 0.02 **	9.87 ± 3.73 20.99 ± 2.56 38.85 ± 3.04 ** 71.53 ± 1.67 ** 96.53 ± 0.41 **	1.58 ± 0.07
(DAC)_2_Cu (**4**)	0.1 0.33 1 3.22 10.20	9.08 ± 0.66 8.04 ± 0.70 6.24 ± 0.71 ** 3.08 ± 0.21 ** 1.32 ± 0.48 **	10.34 ± 1.48 20.78 ± 2.44 38.70 ± 3.82 ** 69.57 ± 1.56 ** 86.58 ± 5.21 **	1.58 ± 0.15
(DAC)_2_Mn (**5**)	0.1 0.33 1 3.22 10.19	8.79 ± 0.68 7.87 ± 0.69 * 5.71 ± 0.31 ** 1.61 ± 0.48 ** 0.29 ± 0.01 **	14.18 ± 2.76 23.20 ± 2.76 * 44.10 ± 0.55 ** 83.69 ± 5.37 ** 97.18 ± 0.14 **	1.24 ± 0.10

Data are represented as mean ± SEM of thee replicates. * *P* ≤ 0.05 and ** *P* ≤ 0.05 compared to FeSO_4_.

**Table 9:** IC_50_ (μM) for cancer cell lines with compounds **1**, **2**, **3**, **5** and cisplatin.

Compounds	HCT-15	MCF-7	SKLU-1
DAC (**1**)	22.32 ± 3.0	21.79 ± 5.0	14.07 ± 3.3
(DAC)_2_Mg (**2**)	11.59± 1.97 *	5.72 ± 0.31 *	8.87 ± 1.56 *
(DAC)_2_Zn (**3**)	7.21 ± 0.61 *	4.92 ± 0.071 *	4.74 ± 0.31 *
(DAC)_2_Mn (**5**)	15.54 ± 1.94 *	13.46 ± 1.63 *	9.23 ± 1.22 *
Cisplatin	10.0 ± 0.9 *	9.4 ± 1.0 *	4.3 ± 0.5 *

Data are represented as mean ± standard error on the mean (SEM) of three replicates, * *P* ≤ 0.05, compared to **1**.

**Table 10:** Body weight (g) and body weight gain (%) of mice given acute oral dose (values are in mean ±SD).

Complexes	Doses (mg/kg)	No. of Animals	Sex	Body Weight at Day			% Body Weight Gain	
				1	8	15	1–8	8–15
(DAC)_2_Mg	1000	3	Male	25.3 ± 0.3	27.0 ± 0.5	30.0 ± 0.4	6.7 ± 0.3	10.7 ± 0.6
(DAC)_2_Mg	3000	3	Male	26.5 ± 0.1	28.6 ± 0.2	32.0 ± 1.5	7.5 ± 0.3	13.3 ± 1.5
(DAC)_2_Zn	1000	3	Male	27.1 ± 0.5	28.6 ± 0.2	31.3 ± 0.5	5.5 ± 0.7	9.8 ± 0.4
(DAC)_2_Zn	3000	3	Male	28.4 ± 0.4	30.0 ± 0.3	33.7 ± 0.3	5.6 ± 0.1	12.3 ± 0.0
(DAC)_2_Cu	1000	3	Male	29.3 ± 0.6	31.4 ± 1.1	33.6 ± 0.3	8.5 ± 0.7	8.0 ± 1.1
(DAC)_2_Cu	3000	3	Male	27.5 ± 0.9	29.4 ± 1.5	30.5 ± 0.8	6.5 ± 0.6	4.1 ± 0.8
(DAC)_2_Mn	1000	3	Male	25.8 ± 0.2	26.9 ± 0.5	29.0 ± 0.4	6.5 ± 0.6	4.1 ± 0.8
(DAC)_2_Mn	3000	3	Male	26.1 ± 1.8	27.6 ± 0.8	29.4 ± 1.3	4.2 ± 1.0	5.8 ± 0.5

**Table 11:** Clinical signs and mortality of mice receiving acute oral dose.

Complexes	Doses (mg/kg)	No. of Animals	Sex	Clinical Signs	Mortality
(DAC)_2_Mg	1000	3	male	normal	0
(DAC)_2_Mg	3000	3	male	normal	0
(DAC)_2_Zn	1000	3	male	normal	0
(DAC)_2_Zn	3000	3	male	normal	0
(DAC)_2_Cu	1000	3	male	bristly fur	0
(DAC)_2_Cu	3000	3	male	bristly fur	0
(DAC)_2_Mn	1000	3	male	bristly fur	0
(DAC)_2_Mn	3000	3	male	bristly fur	0

## Supplementary Materials

The supplementary materials are available online.
