# Supplementary material for: Full Structural Characterization of Homoleptic Complexes of Diacetylcurcumin with Mg, Zn, Cu, and Mn: Cisplatin-level Cytotoxicity in Vitro with Minimal Acute Toxicity in Vivo
Source: Molecules. 2019 Apr 23;24(8):1598. doi: 10.3390/molecules24081598 (PMC6515169; doi:10.3390/molecules24081598)
Supplement: Supplementary file 1 [file molecules-24-01598-s001.pdf]

Supplementary Information

Full Structural Characterization of Homoleptic Complexes of Diacetylcurcumin with Mg, Zn, Cu and Mn: Cisplatin-level Cytotoxicity *in vitro* with Minimal Acute Toxicity *in vivo*

William Meza-Morales, M. Mirian Estévez-Carmona, Yair Alvarez-Ricardo, Marco A. Obregón-Mendoza, Julia Cassani, María Teresa Ramírez-Apan, Carolina Escobedo-Martínez, Manuel Soriano-García, William F. Reynolds, and Raúl G. Enríquez[*]

Corresponding author: *enriquezhabib@gmail.com

Table of Contents

NUCLEAR MAGNETIC RESONANCE	3
ELECTRON PARAMAGNETIC RESONANCE	29
POWDER CRYSTAL X-RAY DIFFRACTION	34
INFRARED SPECTROSCOPY	39
MASS SPECTROMETRY	44
SINGLE CRYSTAL X-RAY DIFFRACTION	53
UV-Vis SPECTROSCOPY	61
FLUORESCENCE SPECTROSCOPY	63
METHODOLOGY OF CYTOTOXICITY ASSAY	66

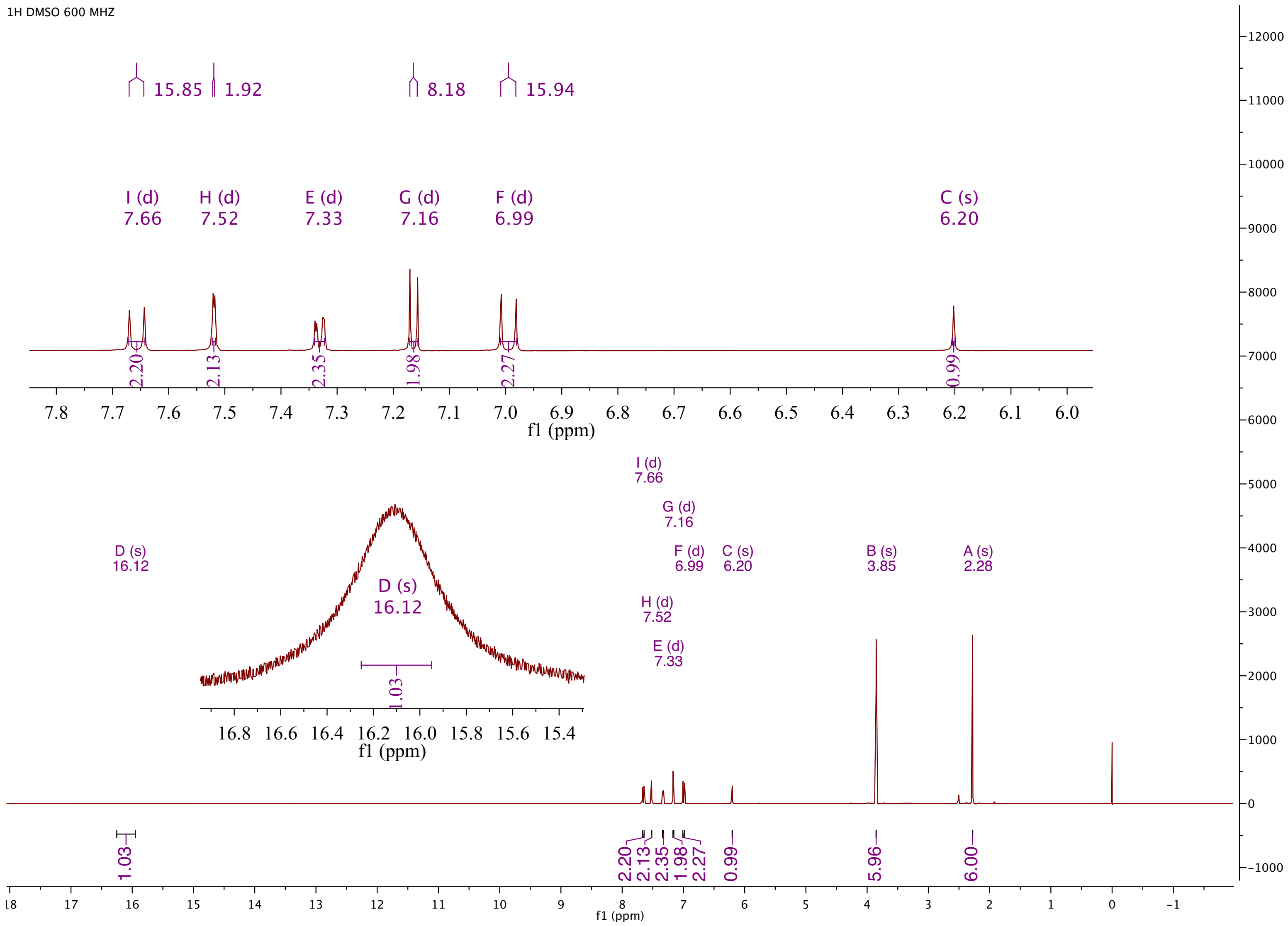

Figure S1. 600 MHz ^{1}H NMR spectrum of diacetyl-curcumin.

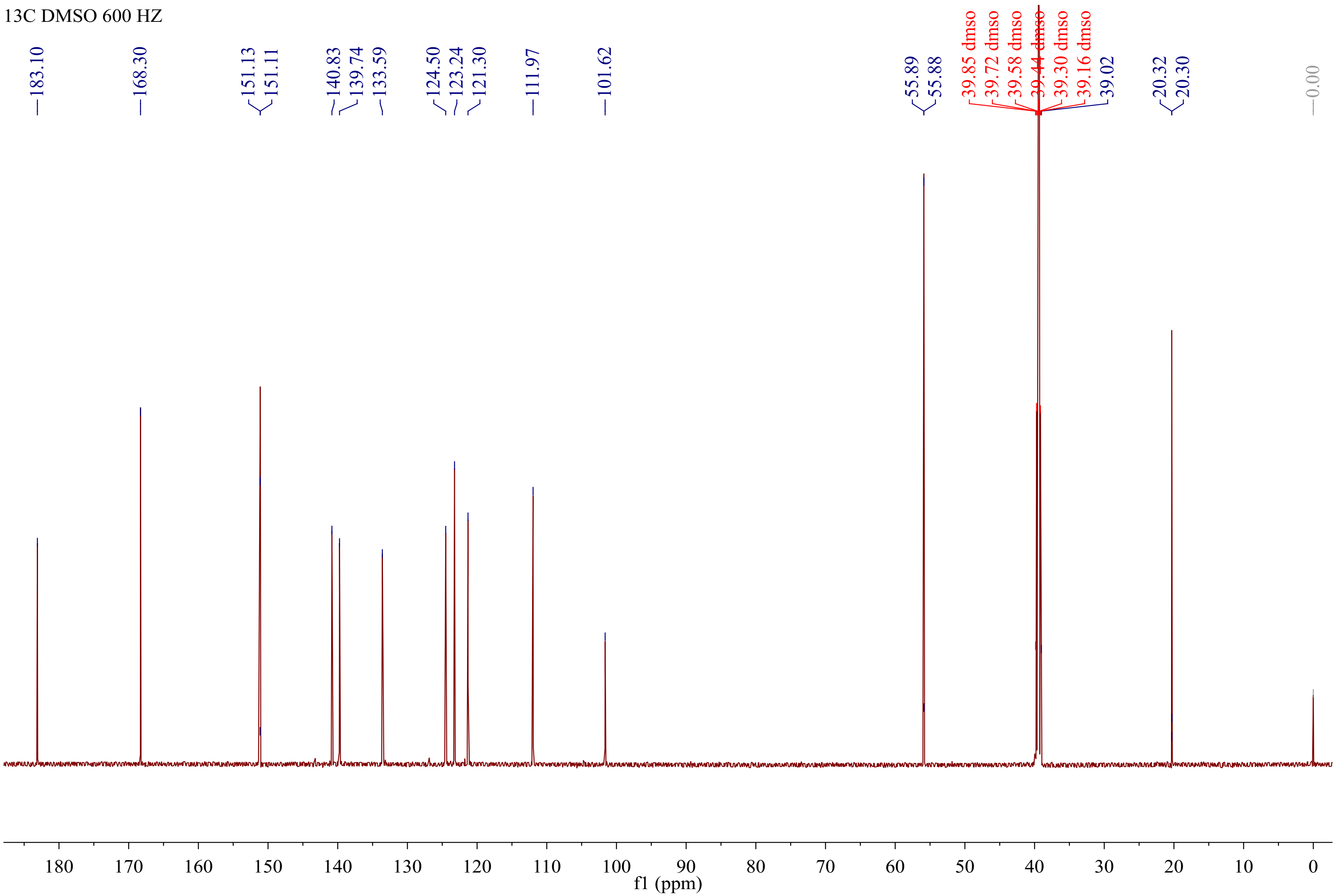

Figure S2. 150 MHz ^{13}C NMR spectrum of diacetyl-curcumin.

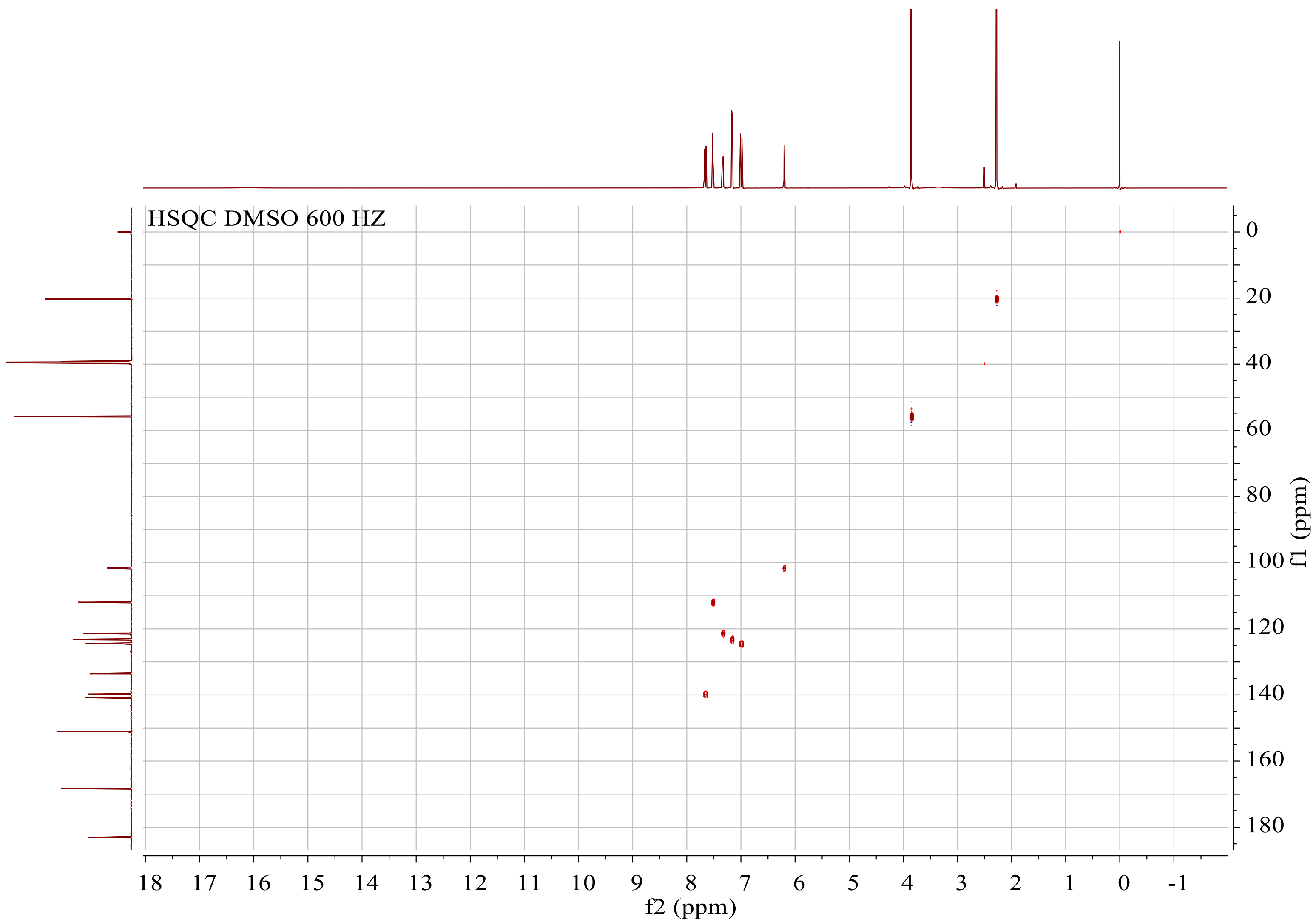

Figure S3. 600 MHz HSQC NMR spectrum of diacetyl-curcumin.

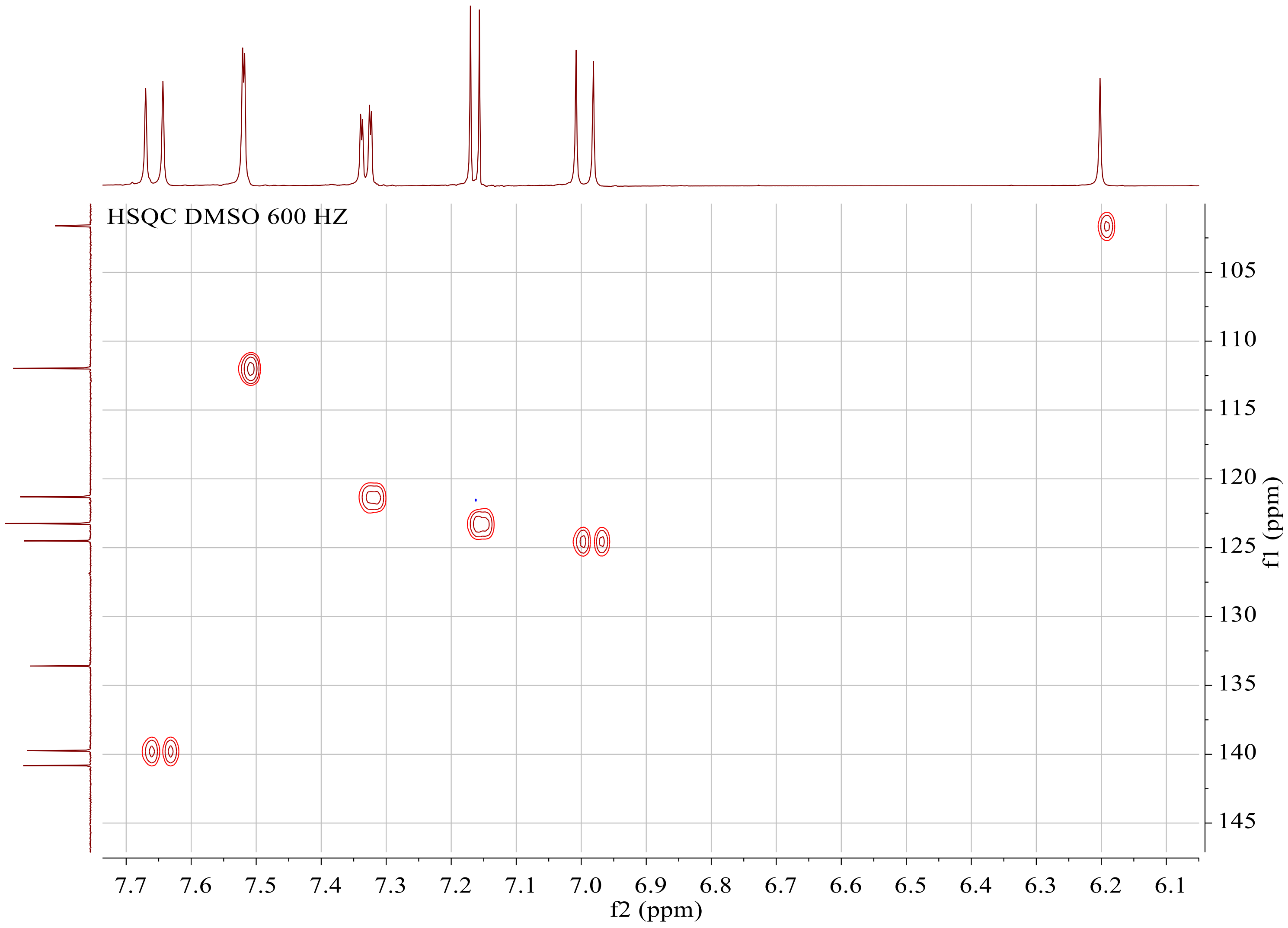

Figure S4. 600 MHz HSQC NMR spectrum expansion of diacetyl-curcumin.

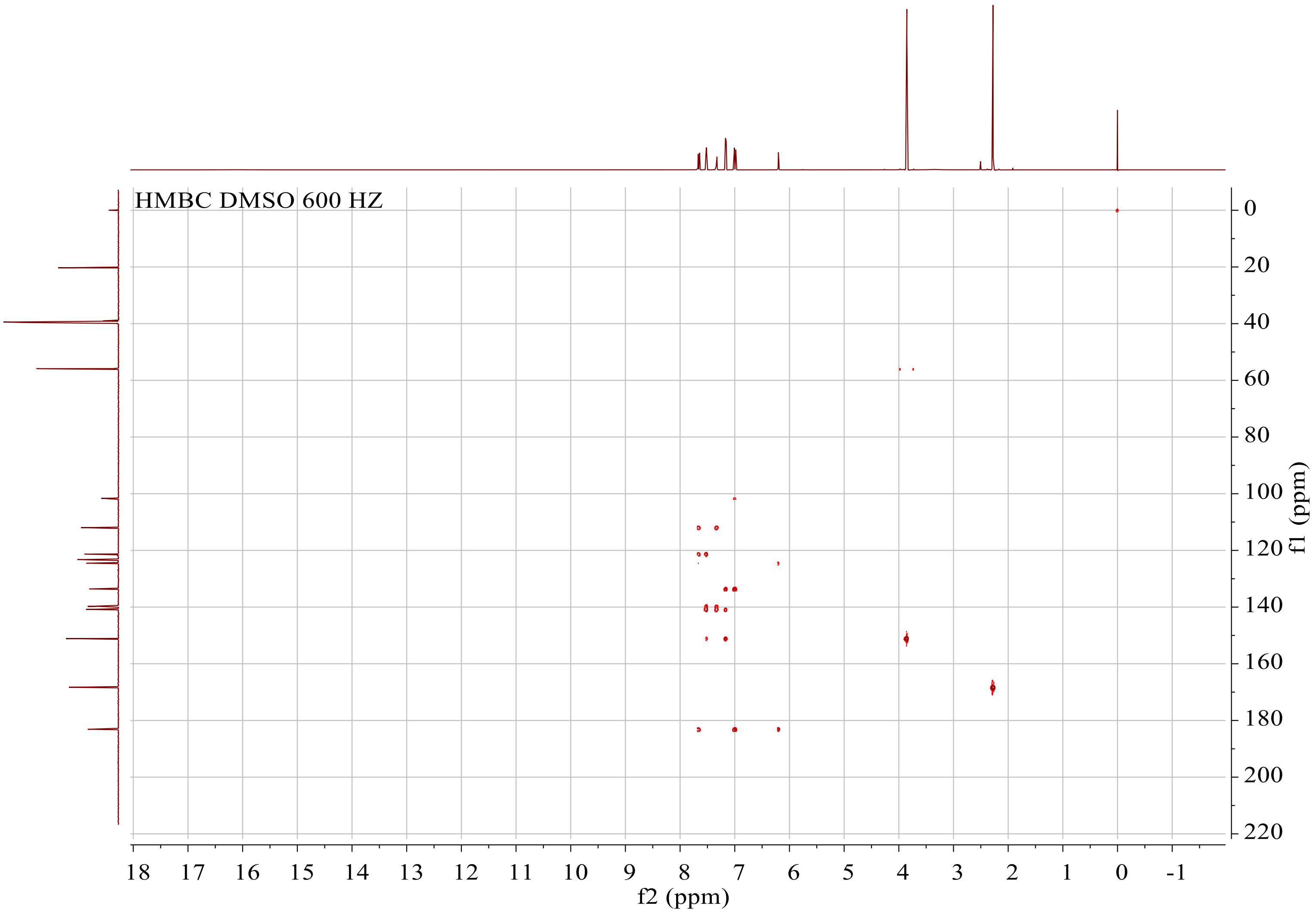

Figure S5. 600 MHz HMBC NMR spectrum of diacetyl-curcumin.

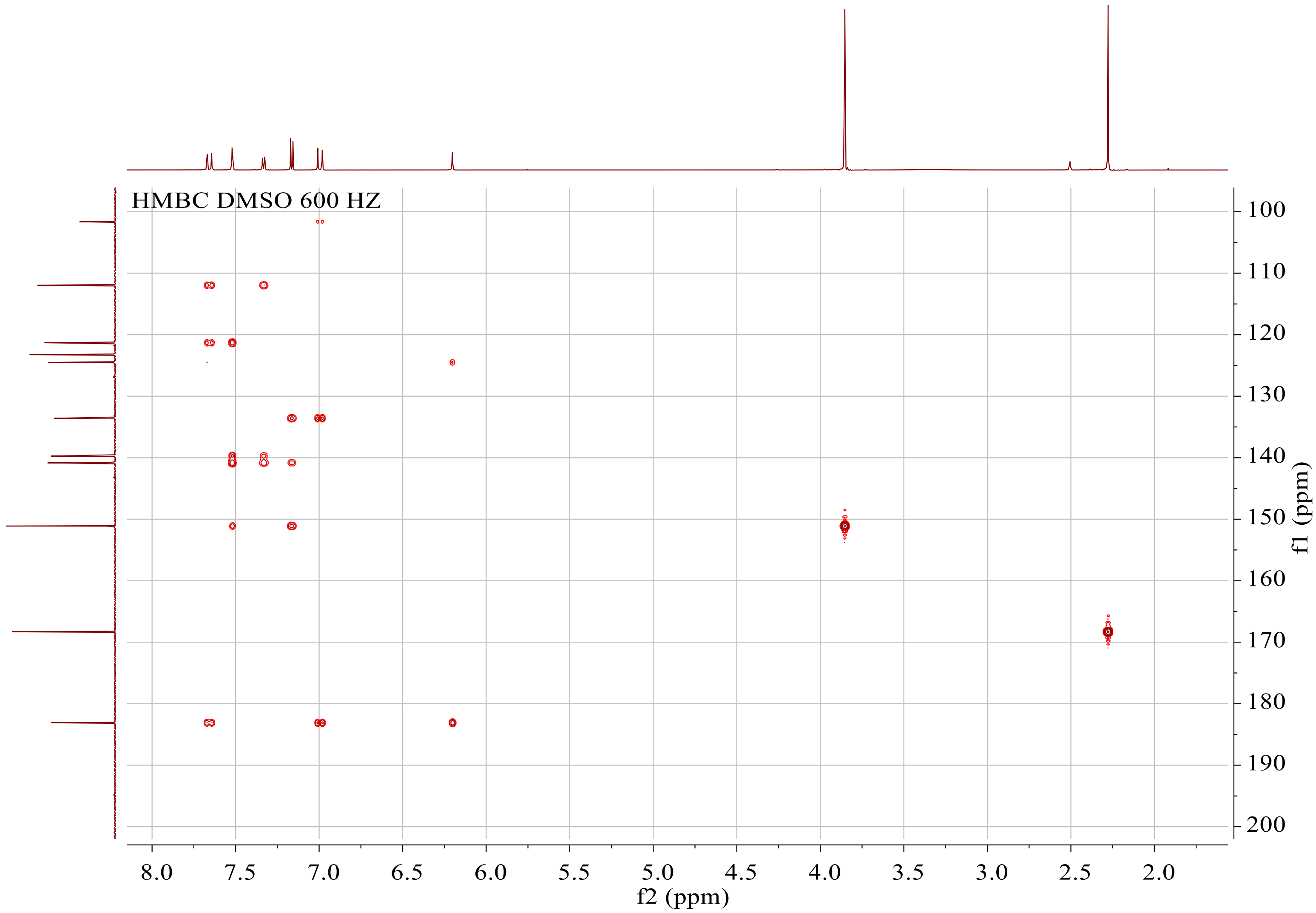

Figure 6. 600 MHz HMBC NMR spectrum expansion of diacetyl-curcumin.

1H DMSO 600 HZ

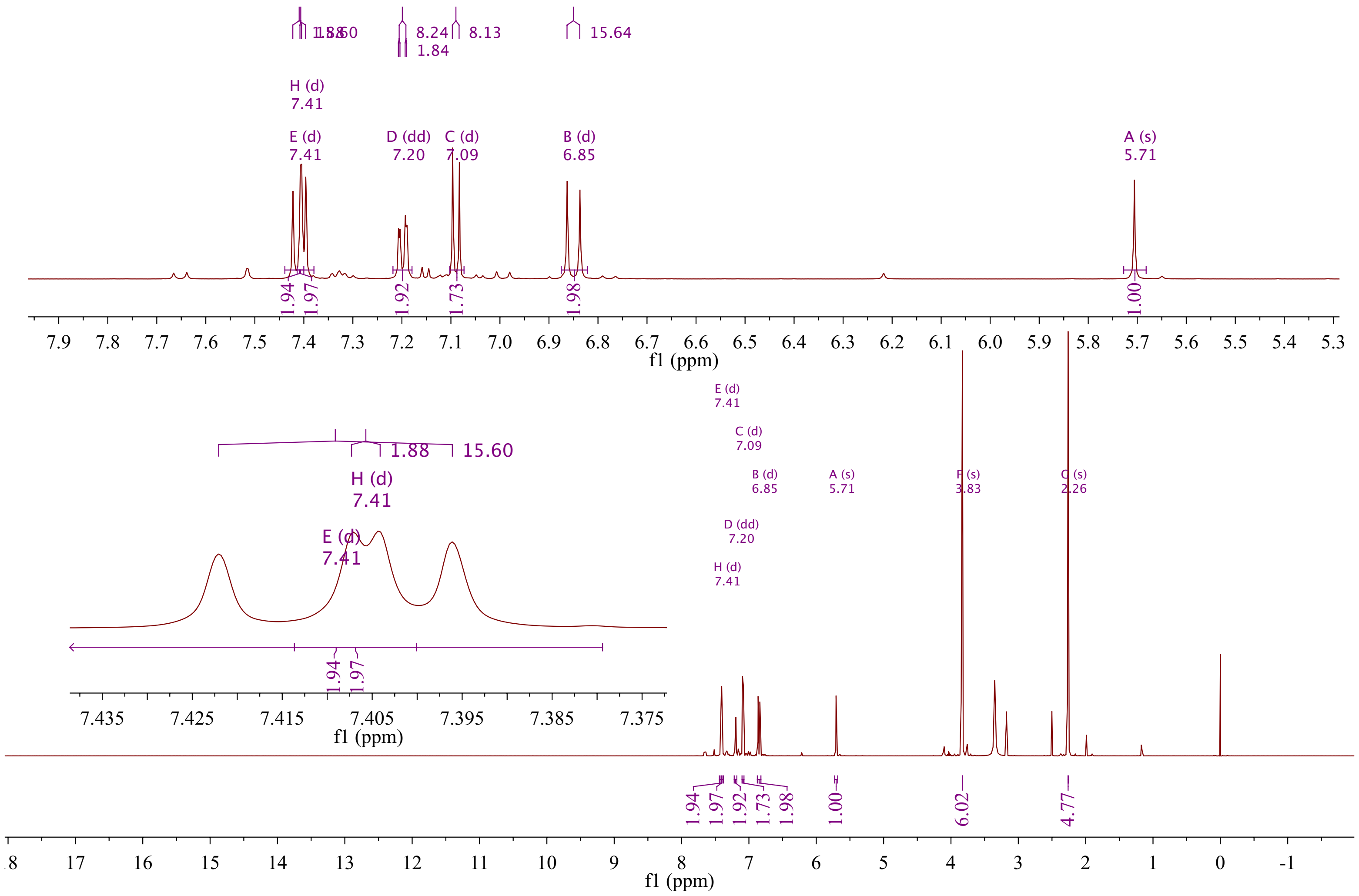

Figure S7. 600 MHz ^1H NMR spectrum of diacetyl-curcumin with Mg (II).

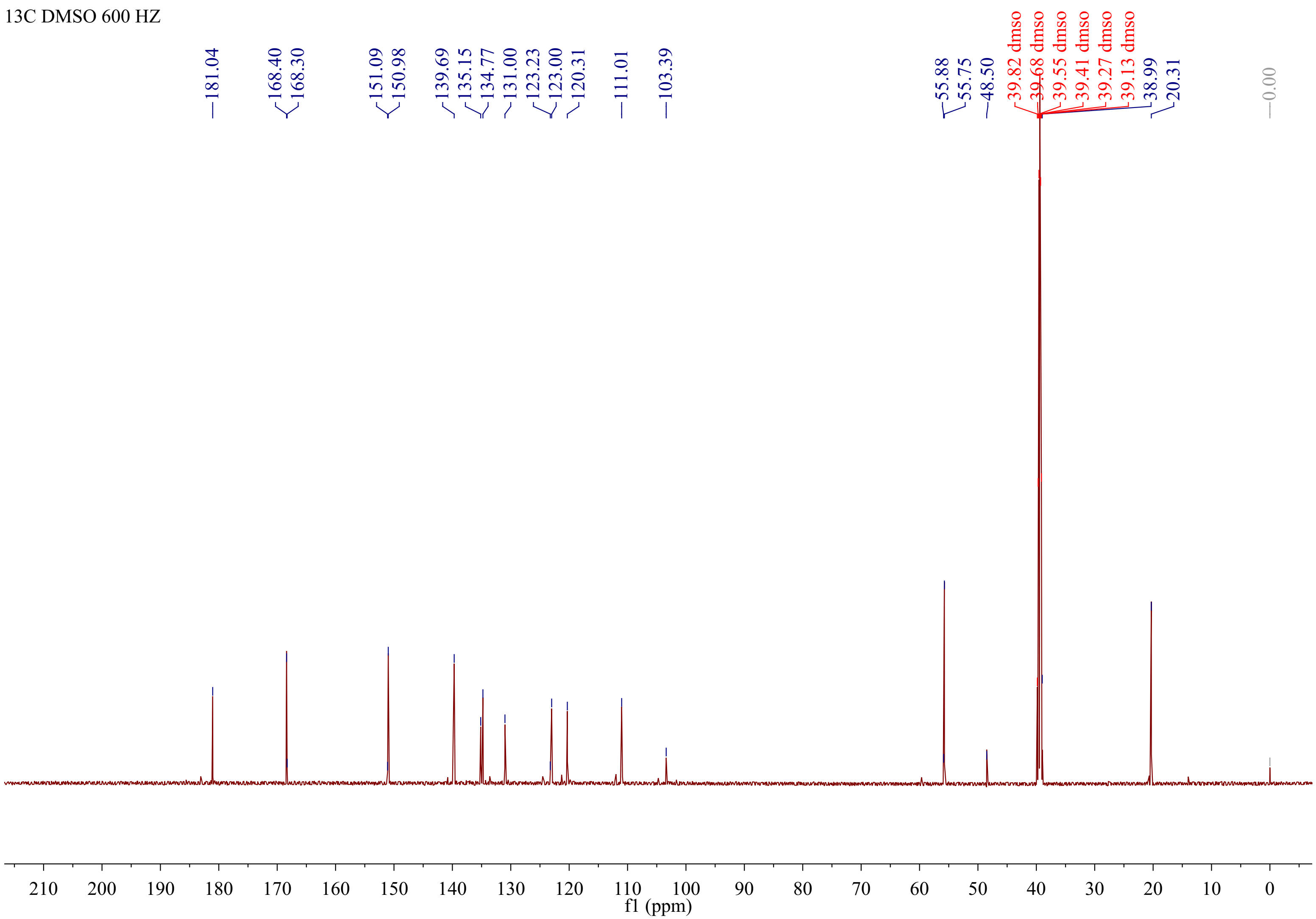

Figure S8. 150 MHz ^{13}C NMR spectrum of diacetyl-curcumin with Mg (II).

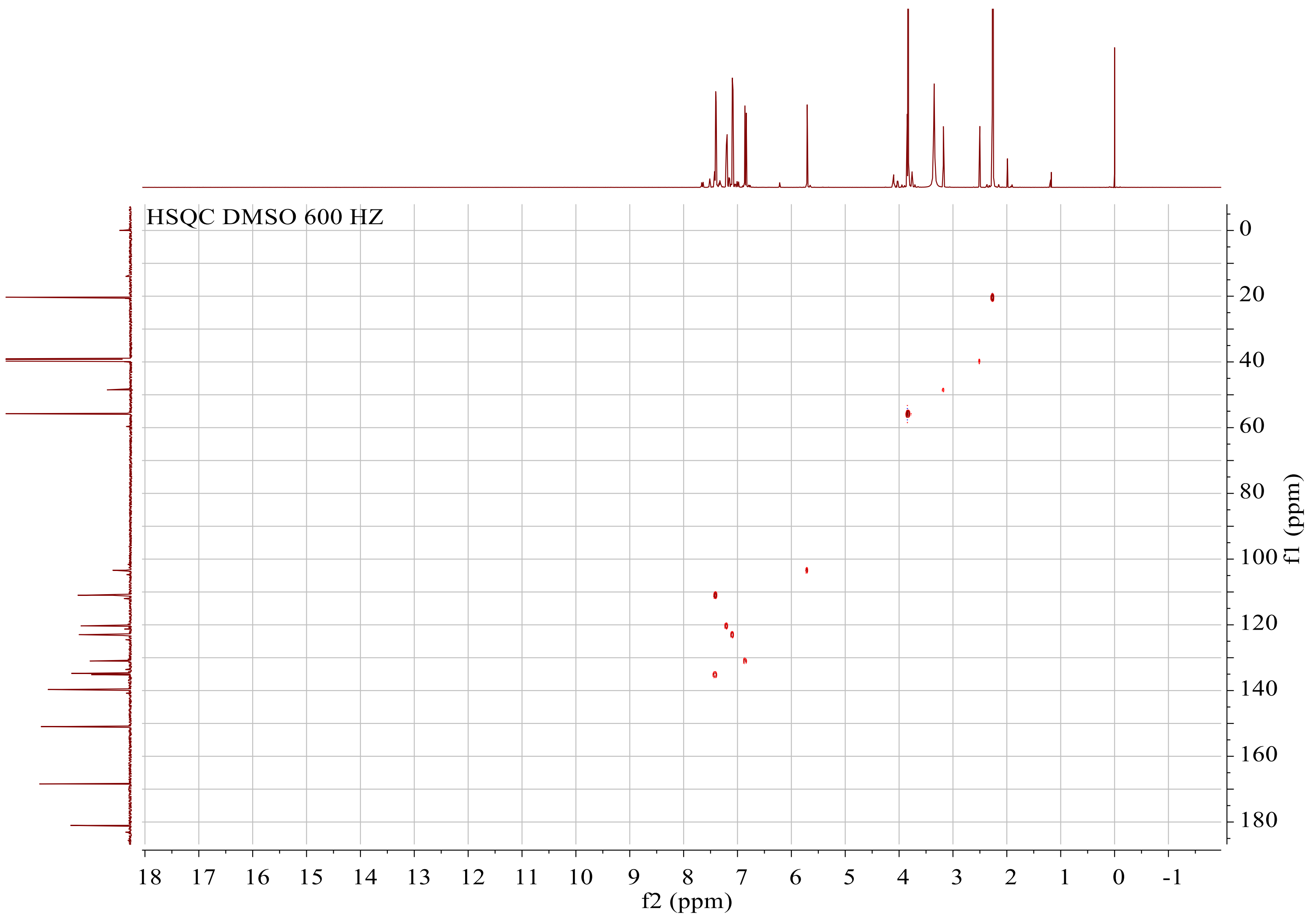

Figure S9. 600 MHz HSQC NMR spectrum of diacetyl-curcumin with Mg (II).

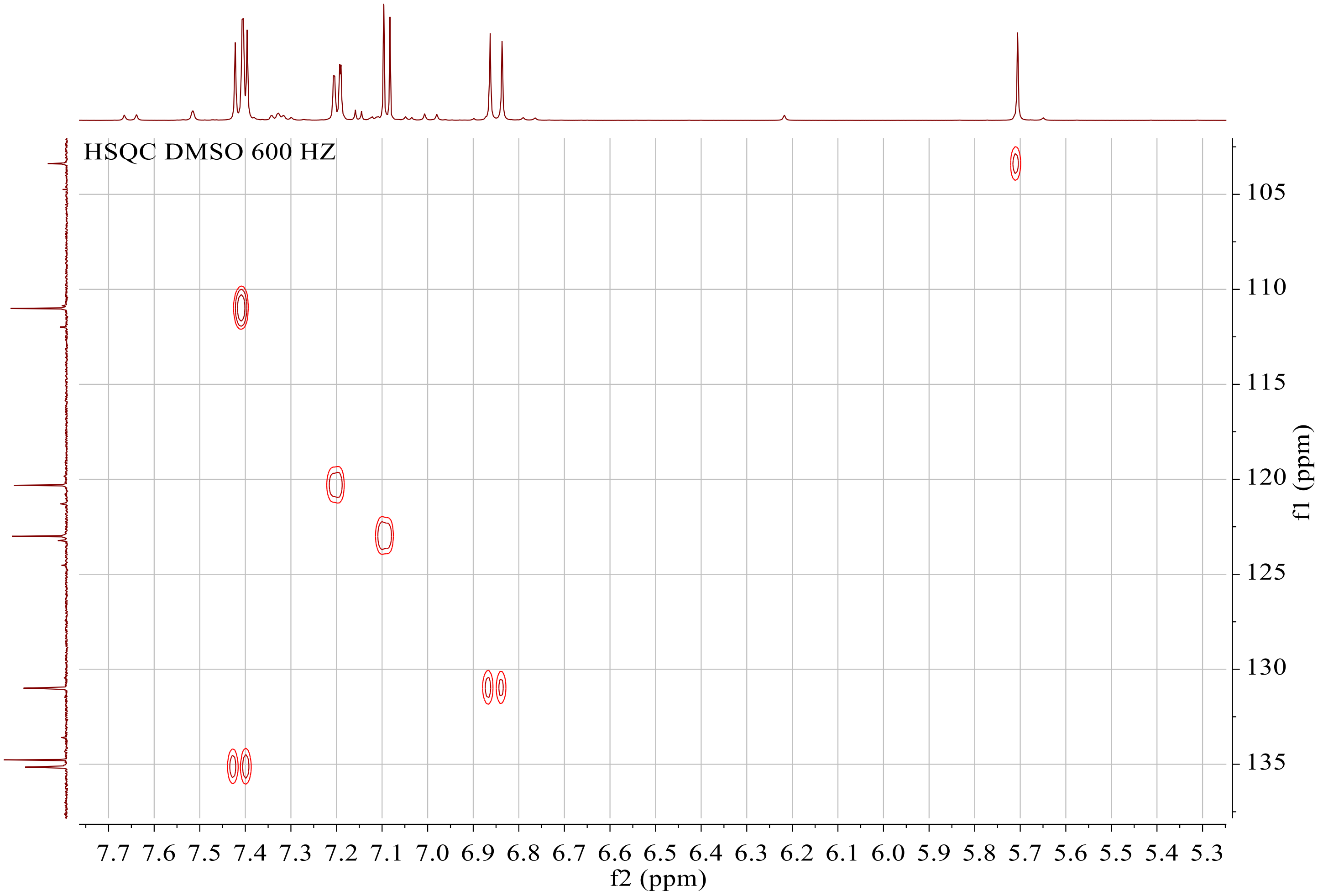

Figure S10. 600 MHz HSQC NMR spectrum expansion of diacetyl-curcumin with Mg (II).

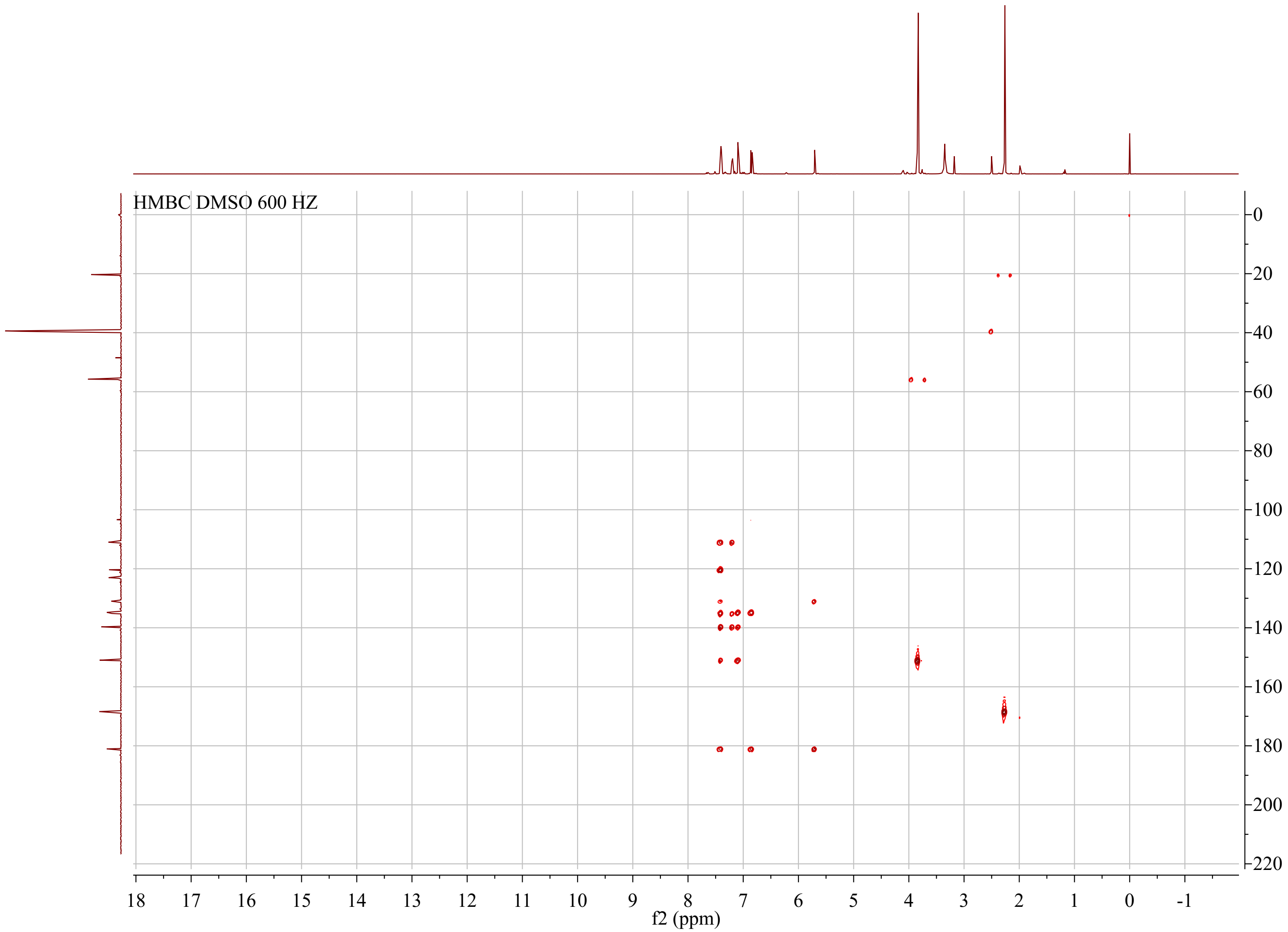

Figure S11. 600 MHz HMBC NMR spectrum of diacetyl-curcumin with Mg (II).

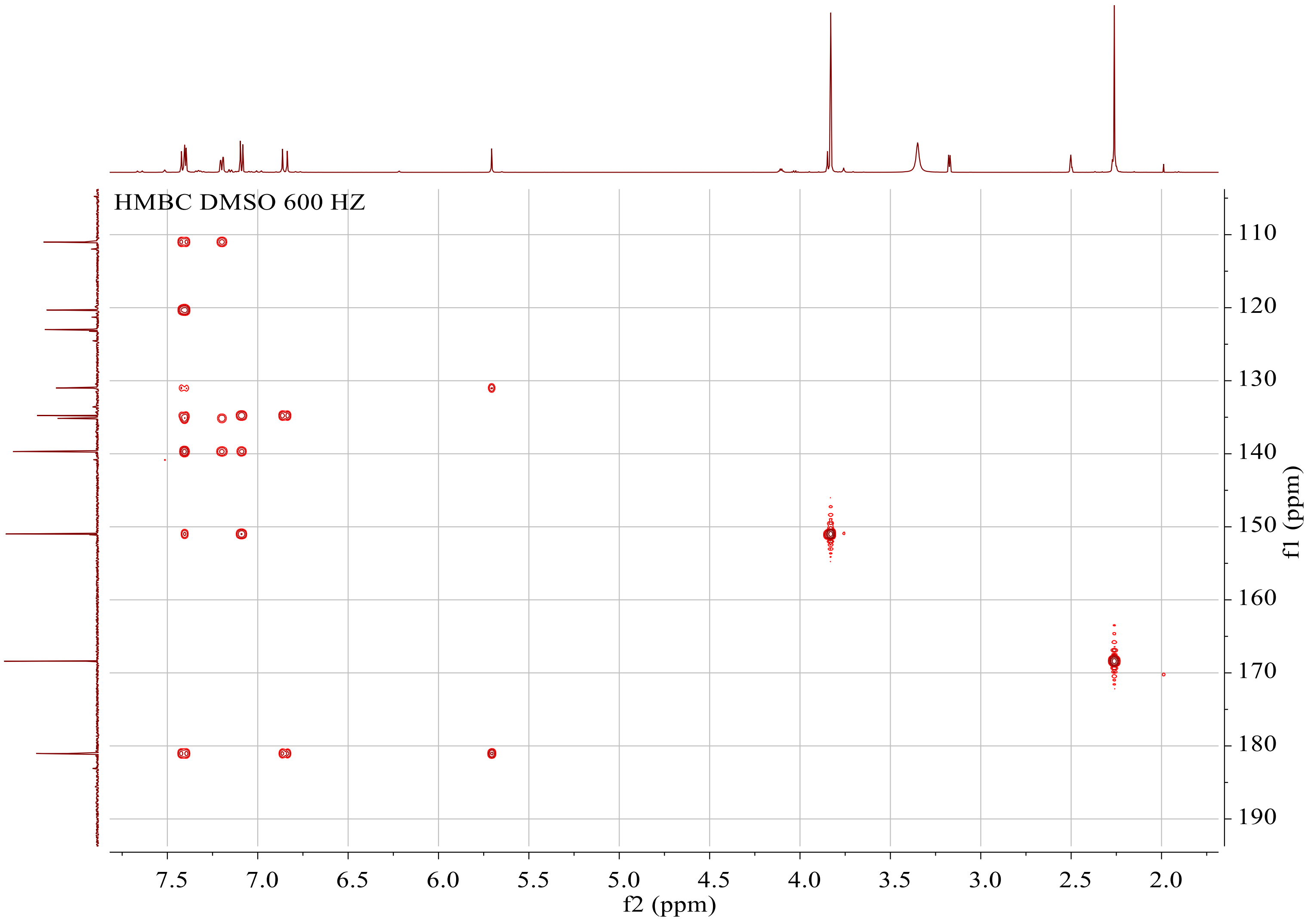

Figure S12. 600 MHz HMBC NMR spectrum expansion of diacetyl-curcumin with Mg (II).

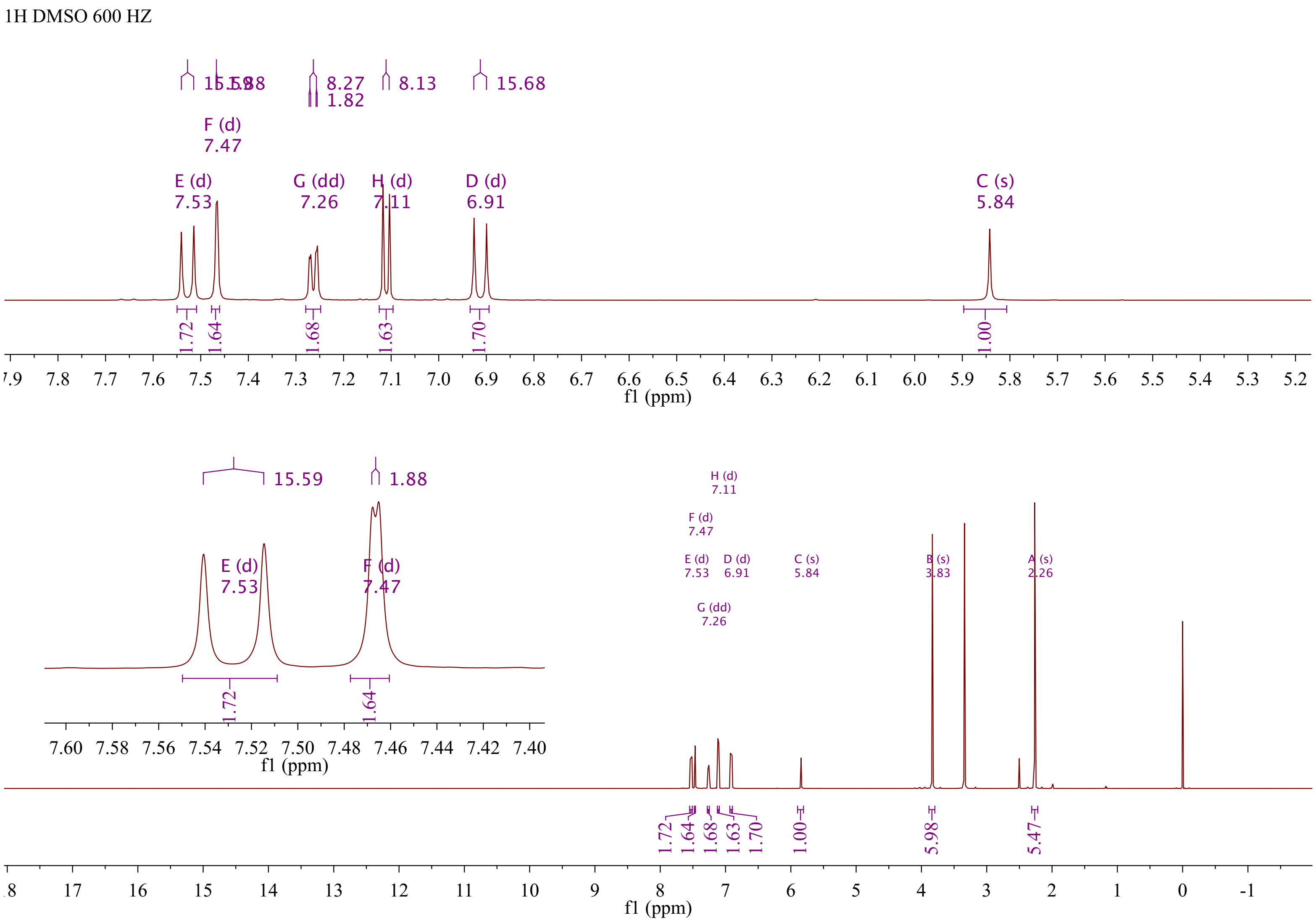

Figure S13. 600 MHz ^{1}H NMR spectrum of diacetyl-curcumin with Zn (II).

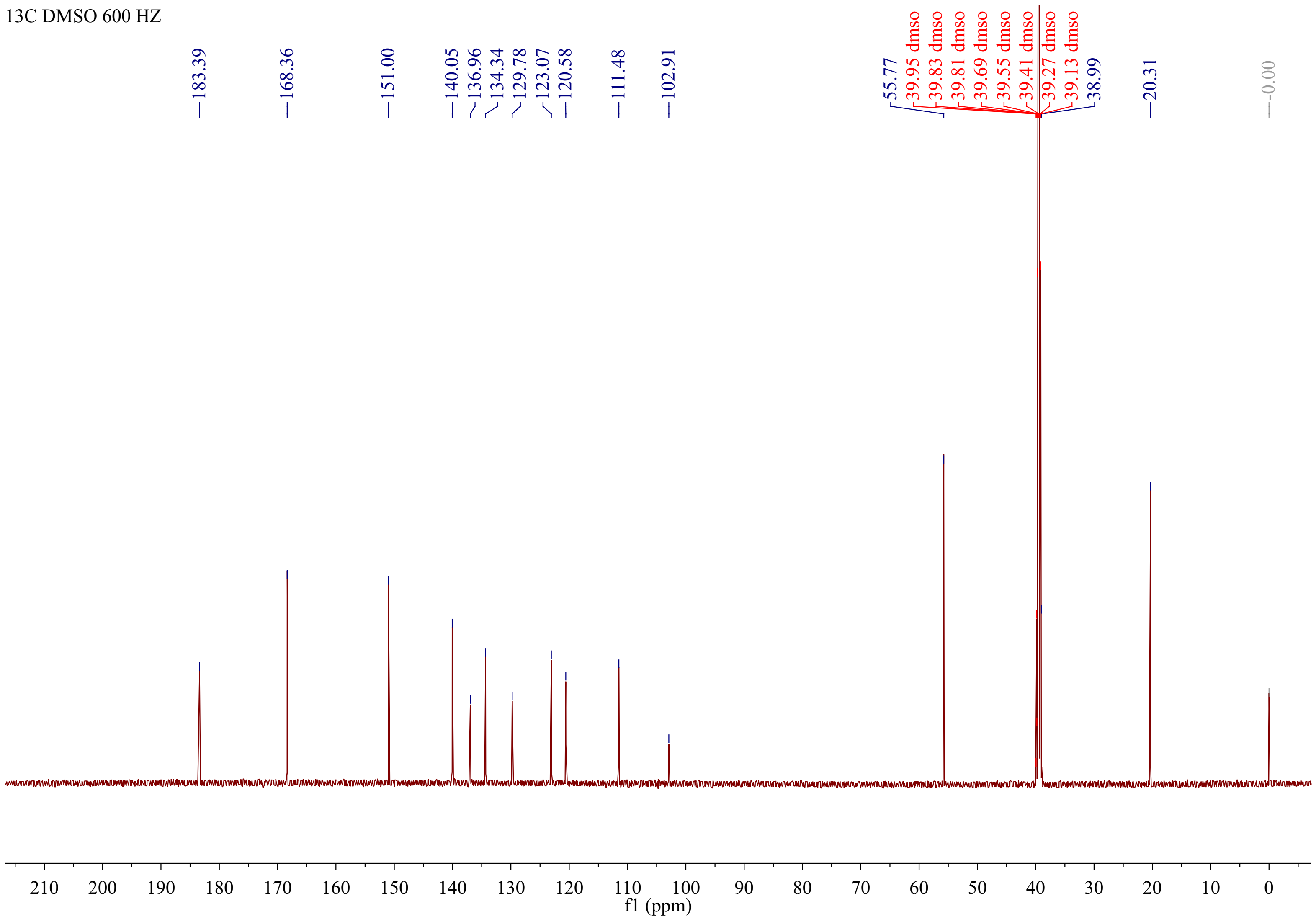

Figure S14. 150 MHz ^{13}C NMR spectrum of diacetyl-curcumin with Zn (II).

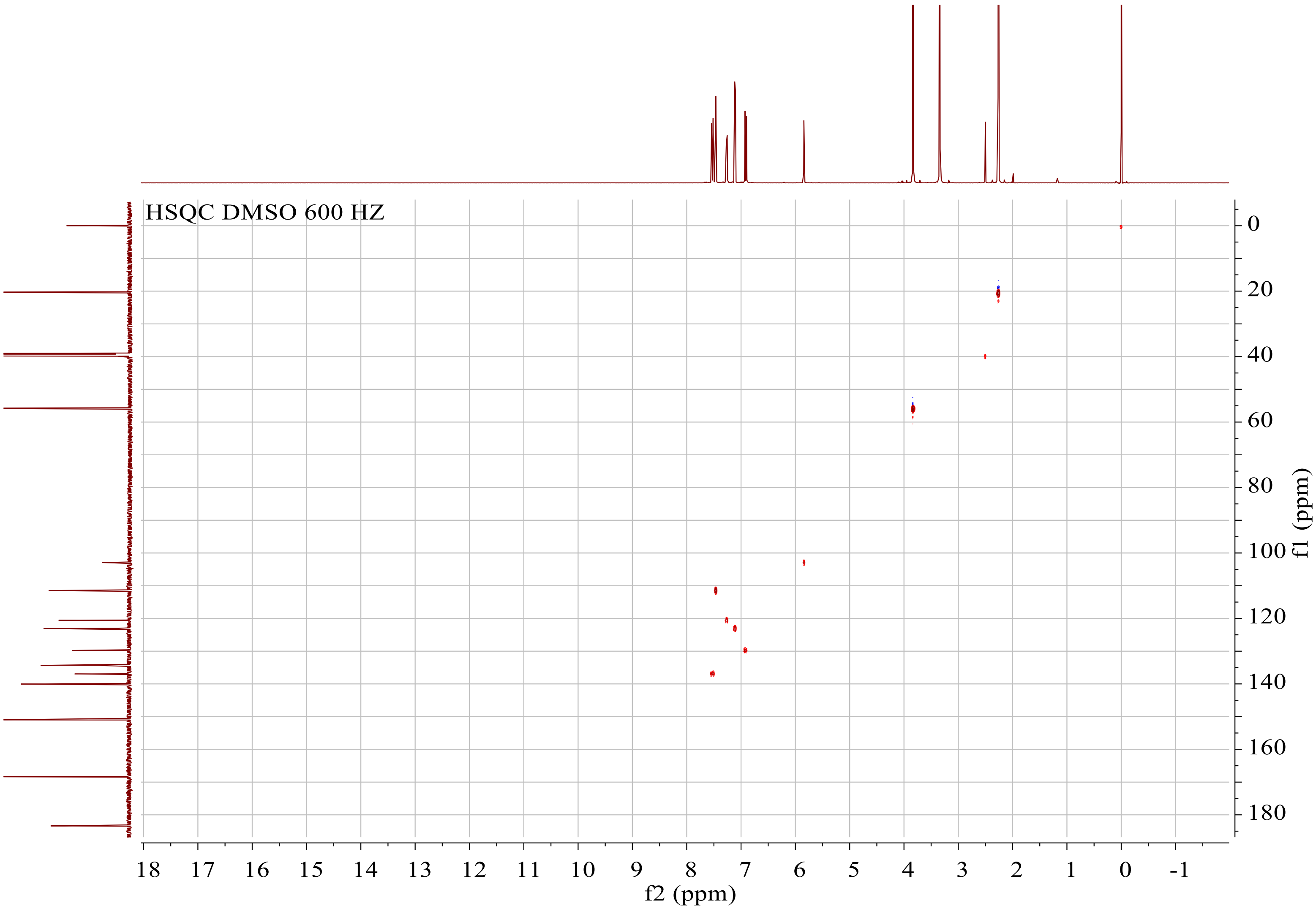

Figure S15. 600 MHz HSQC NMR spectrum of diacetyl-curcumin with Zn (II).

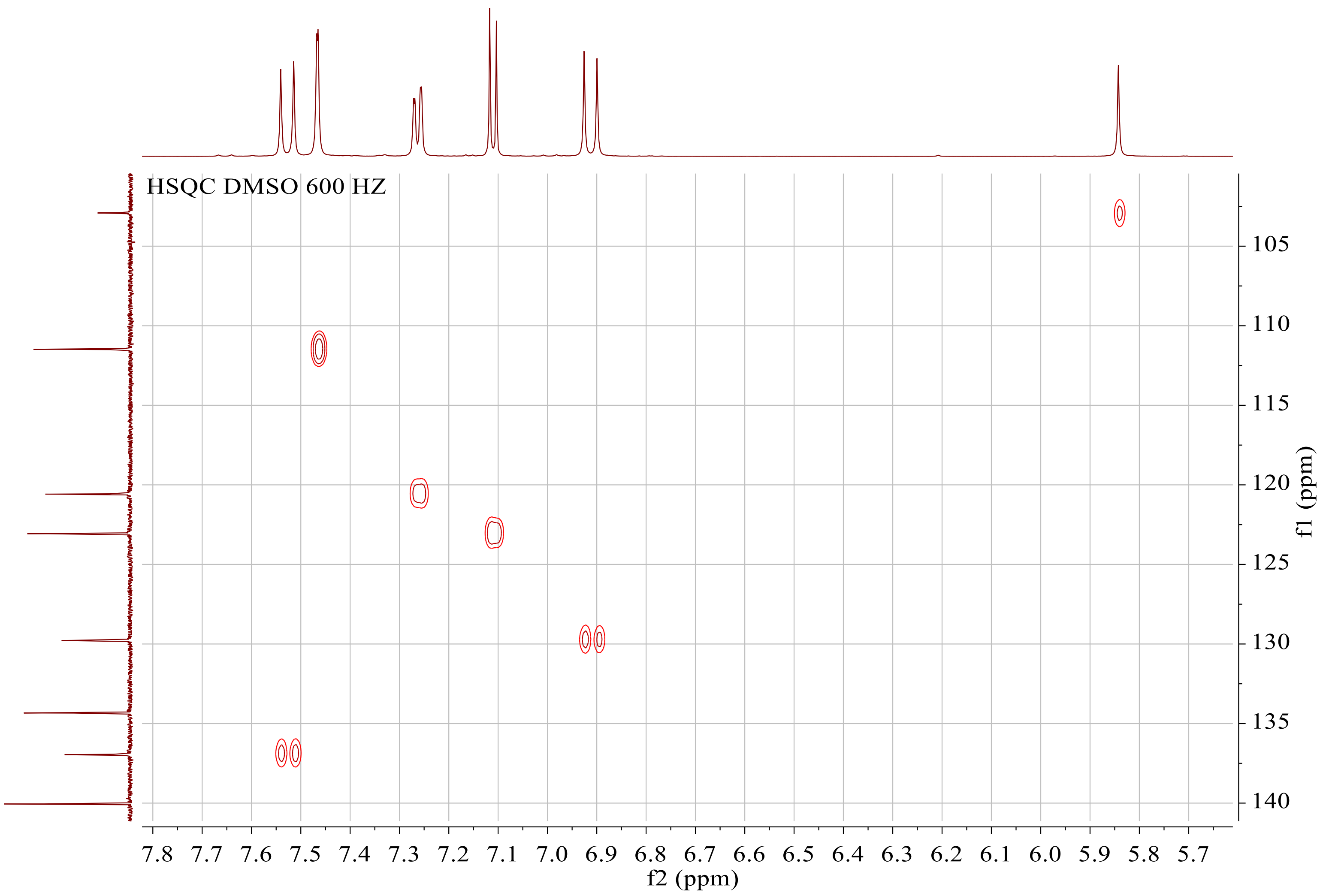

Figure S16. 600 MHz HSQC NMR spectrum expansion of diacetyl-curcumin with Zn (II).

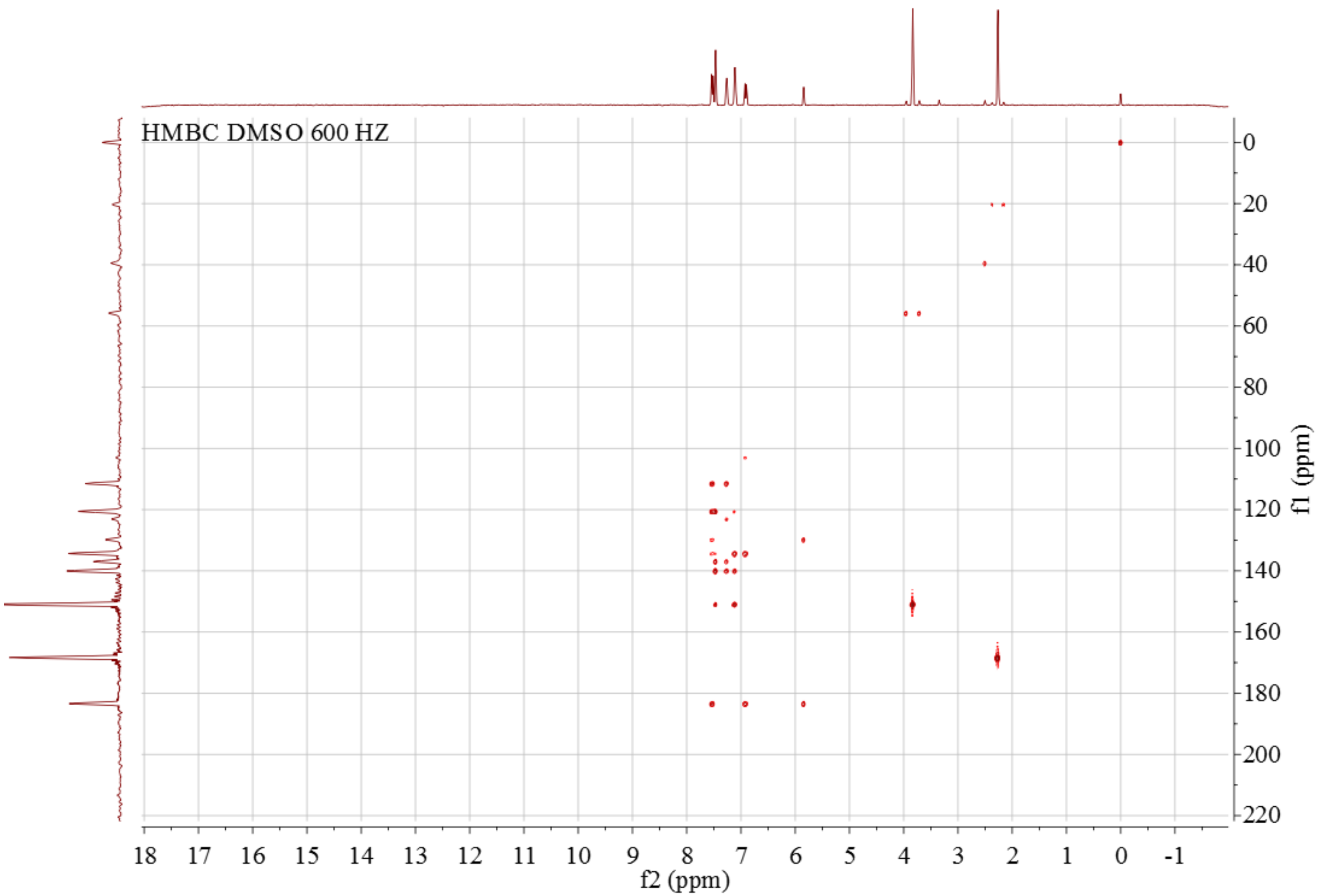

Figure S17. 600 MHz HMBC NMR spectrum of diacetyl-curcumin with Zn (II).

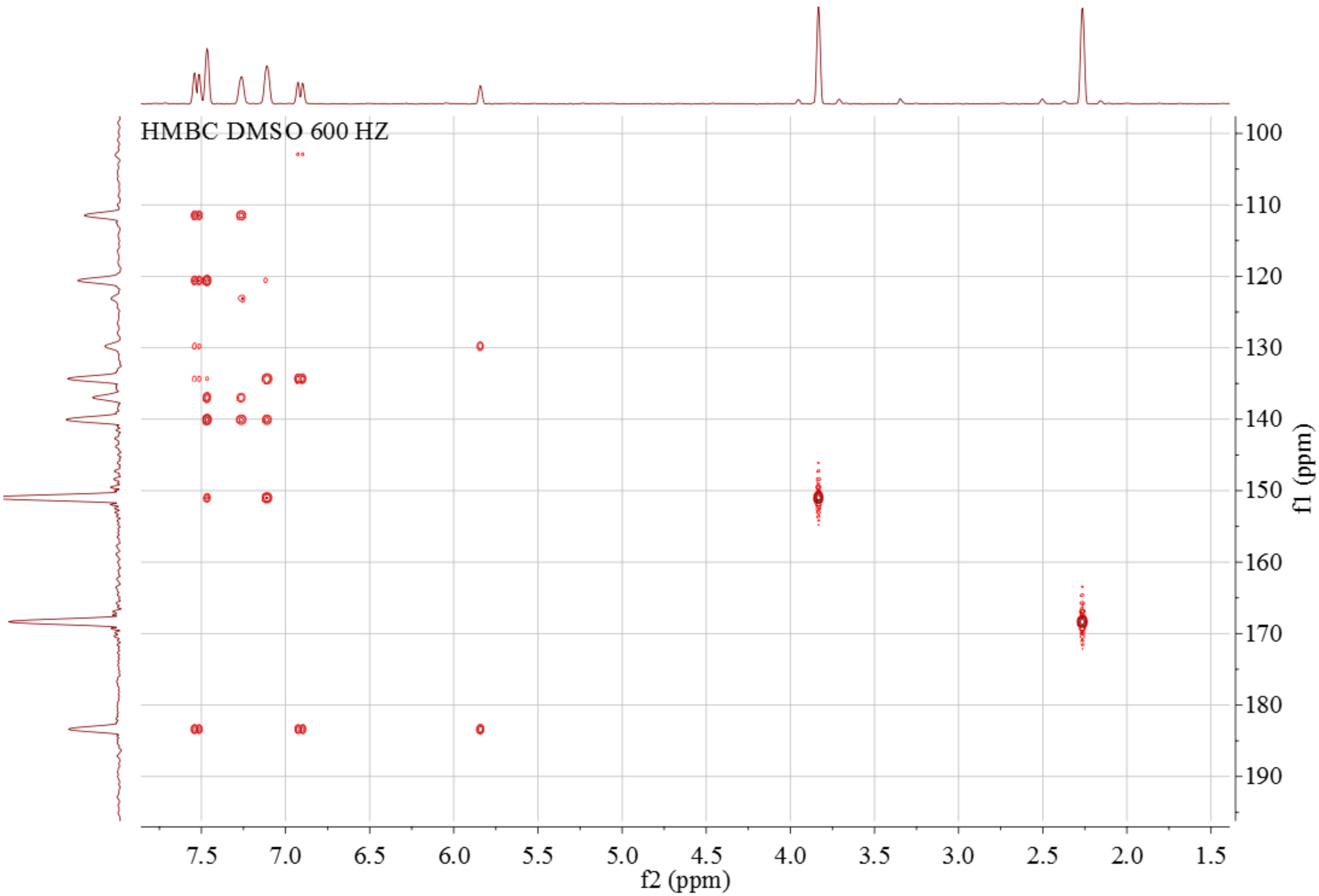

Figure S18. 600 MHz HMBC NMR spectrum expansion of diacetyl-curcumin with Zn (II).

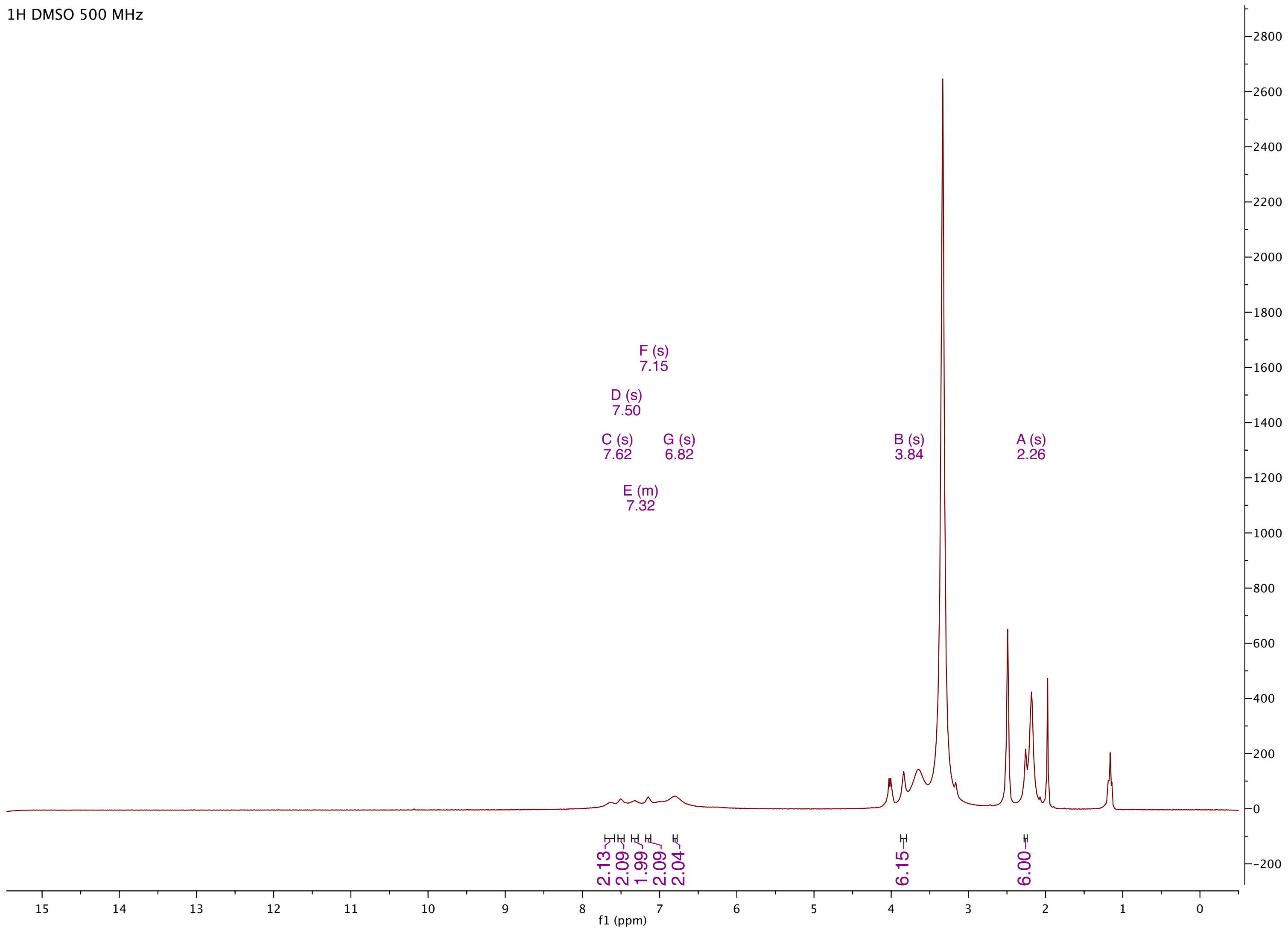

Figure S19. 500 MHz ^{1}H NMR spectrum of diacetyl-curcumin with Cu (II).

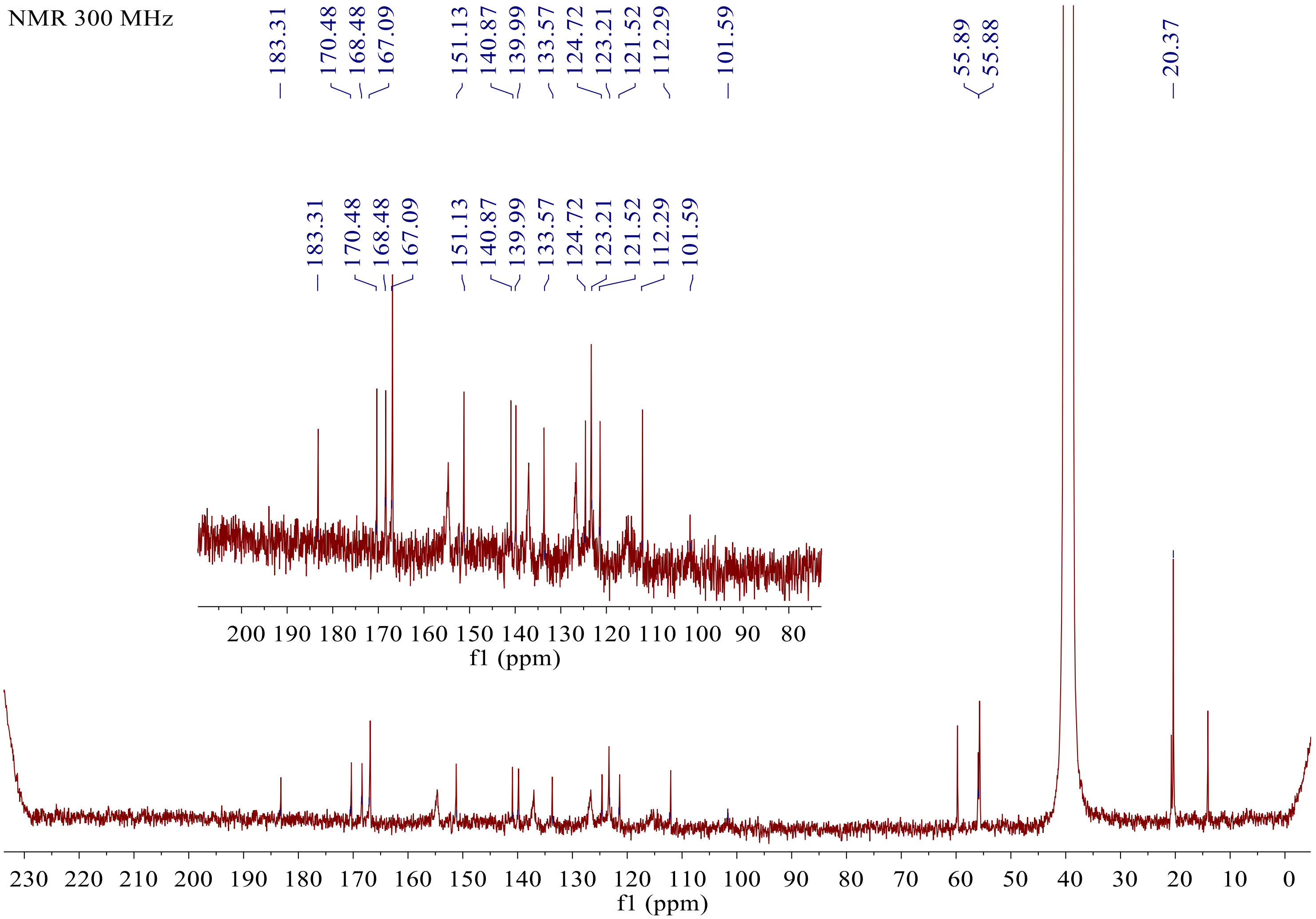

Figure S20. 125 MHz ^{13}C NMR spectrum of diacetyl-curcumin with Cu (II).

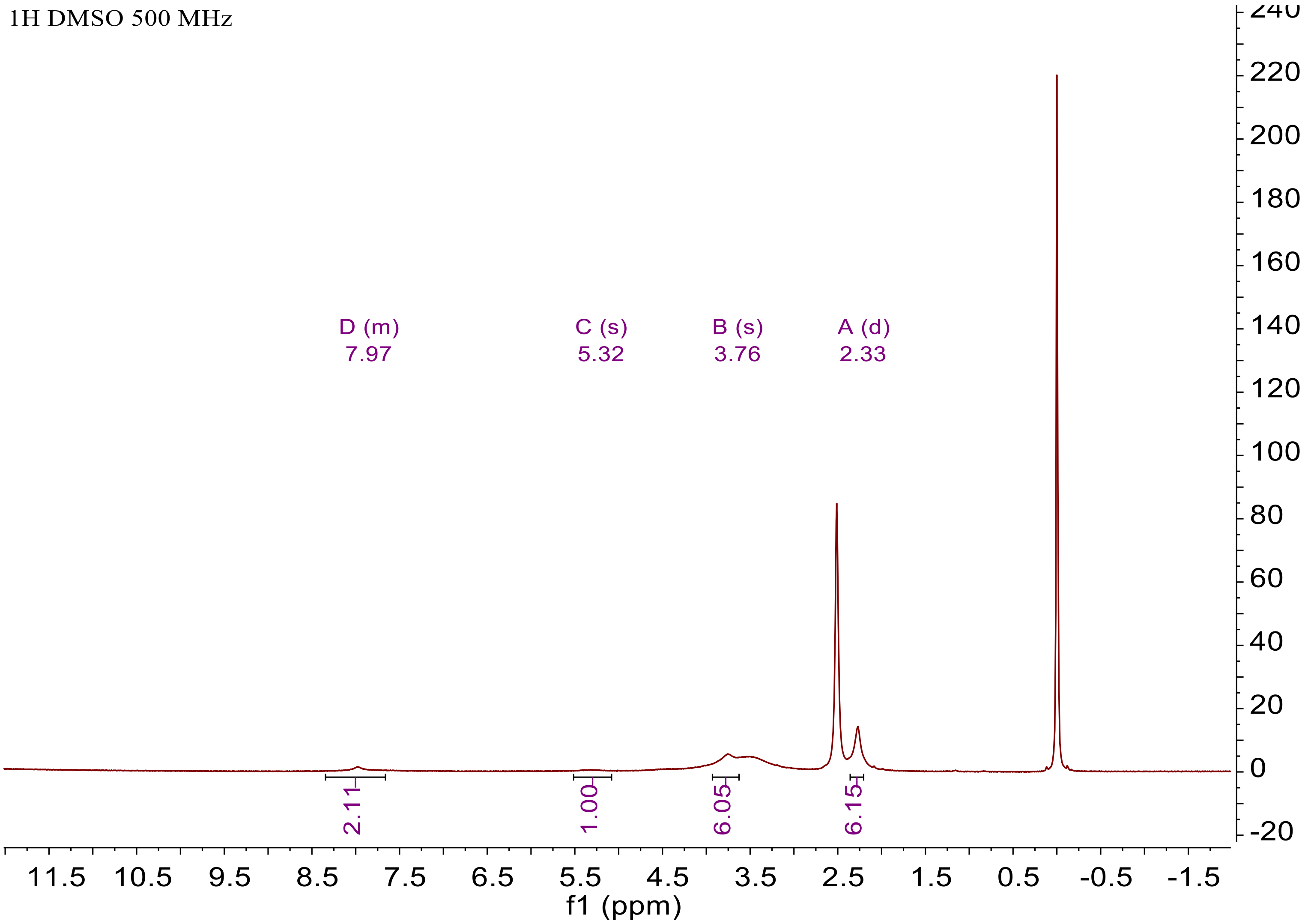

Figure S21. 500 MHz ^{1}H NMR spectrum of diacetyl-curcumin with Mn (II).

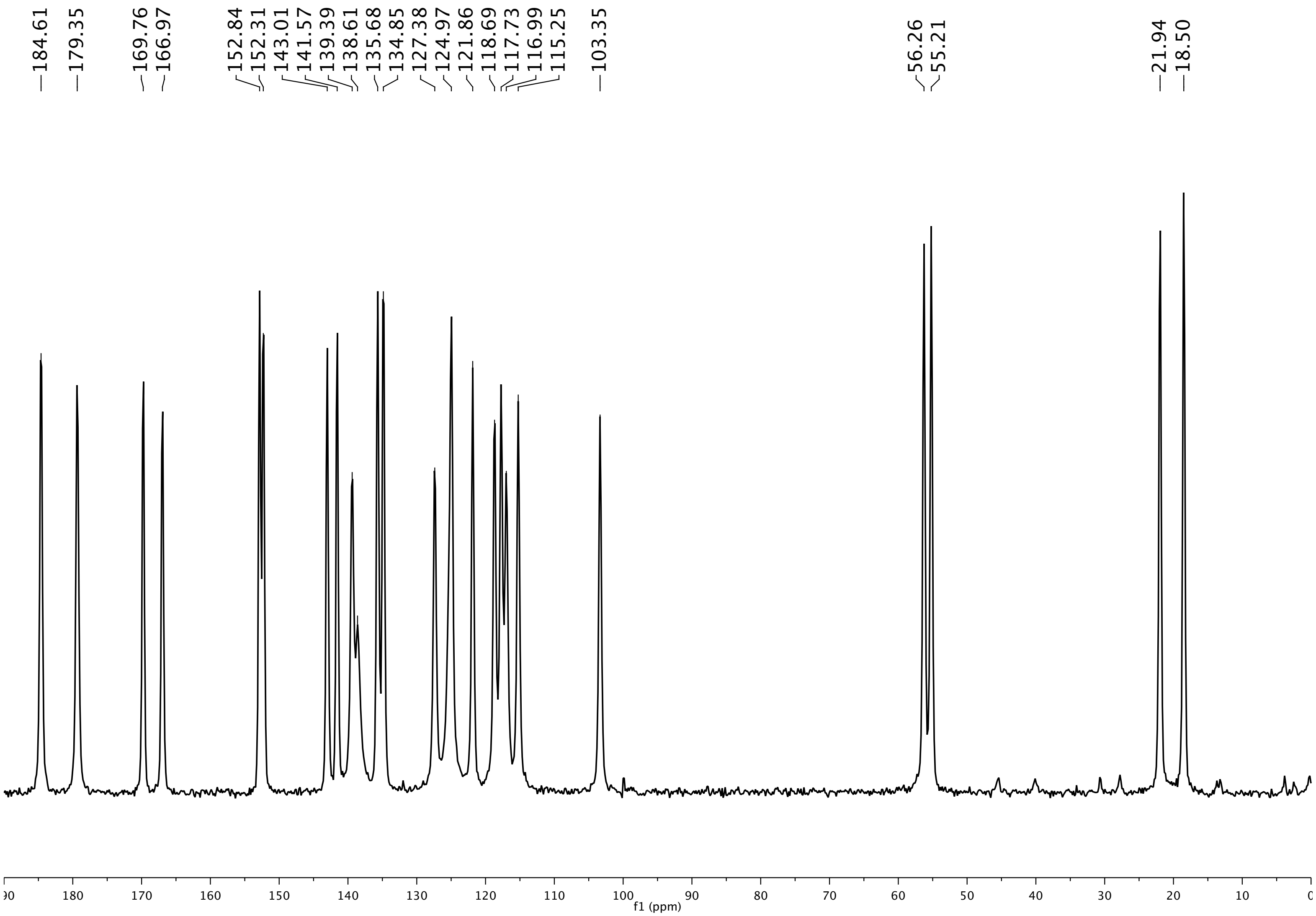

Figura S22. 100 MHz Solid state ^{13}C NMR spectrum of diacetyl-curcumin.

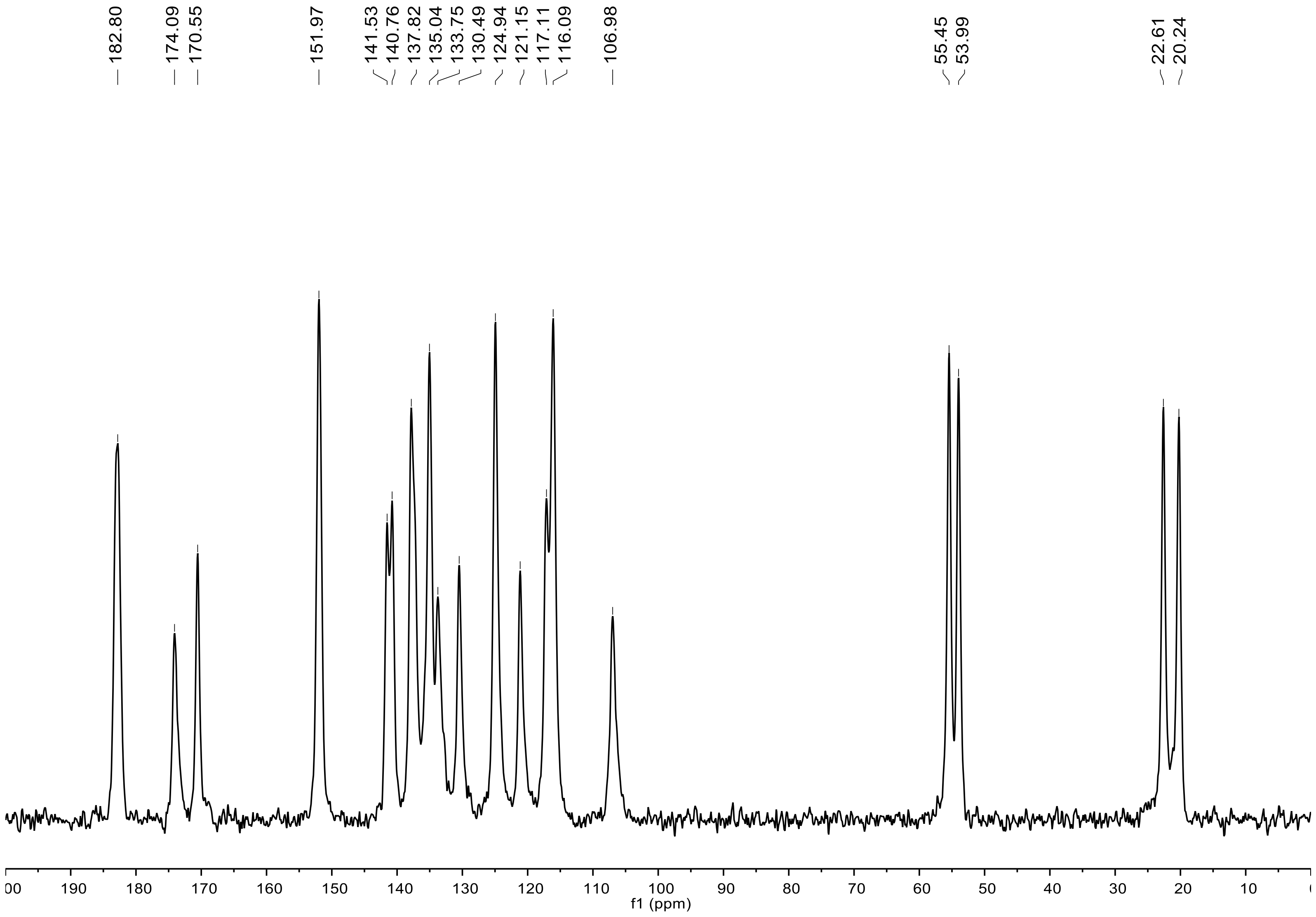

Figura S23. 100 MHz Solid state ^{13}C NMR spectrum of diacetyl-curcumin with Mg (II).

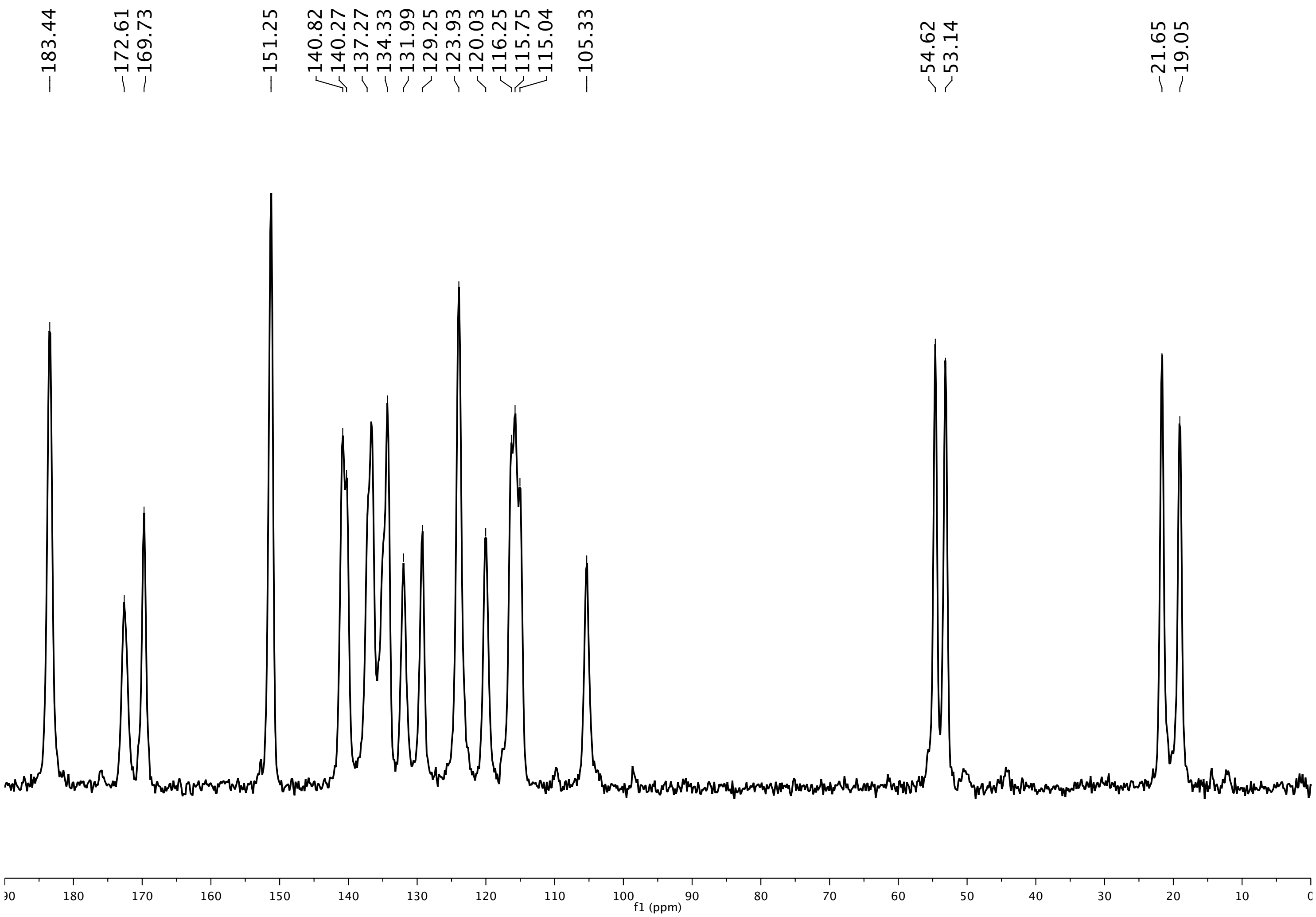

Figura S24. 100 MHz Solid state ^{13}C NMR spectrum of diacetyl-curcumin with Zn (II).

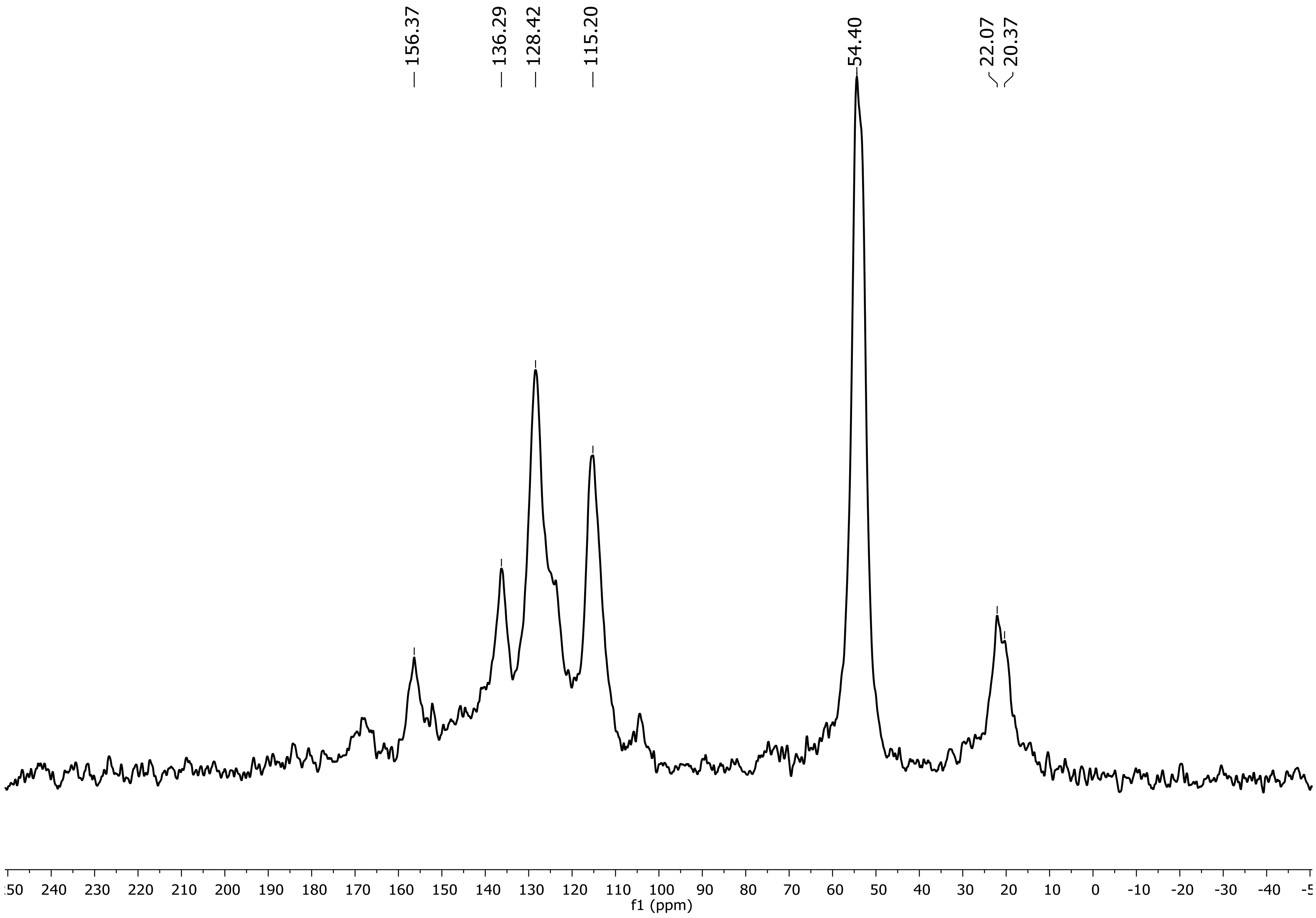

Figura S25. 100 MHz Solid state ^{13}C NMR spectrum of diacetyl-curcumin.with Cu (II).

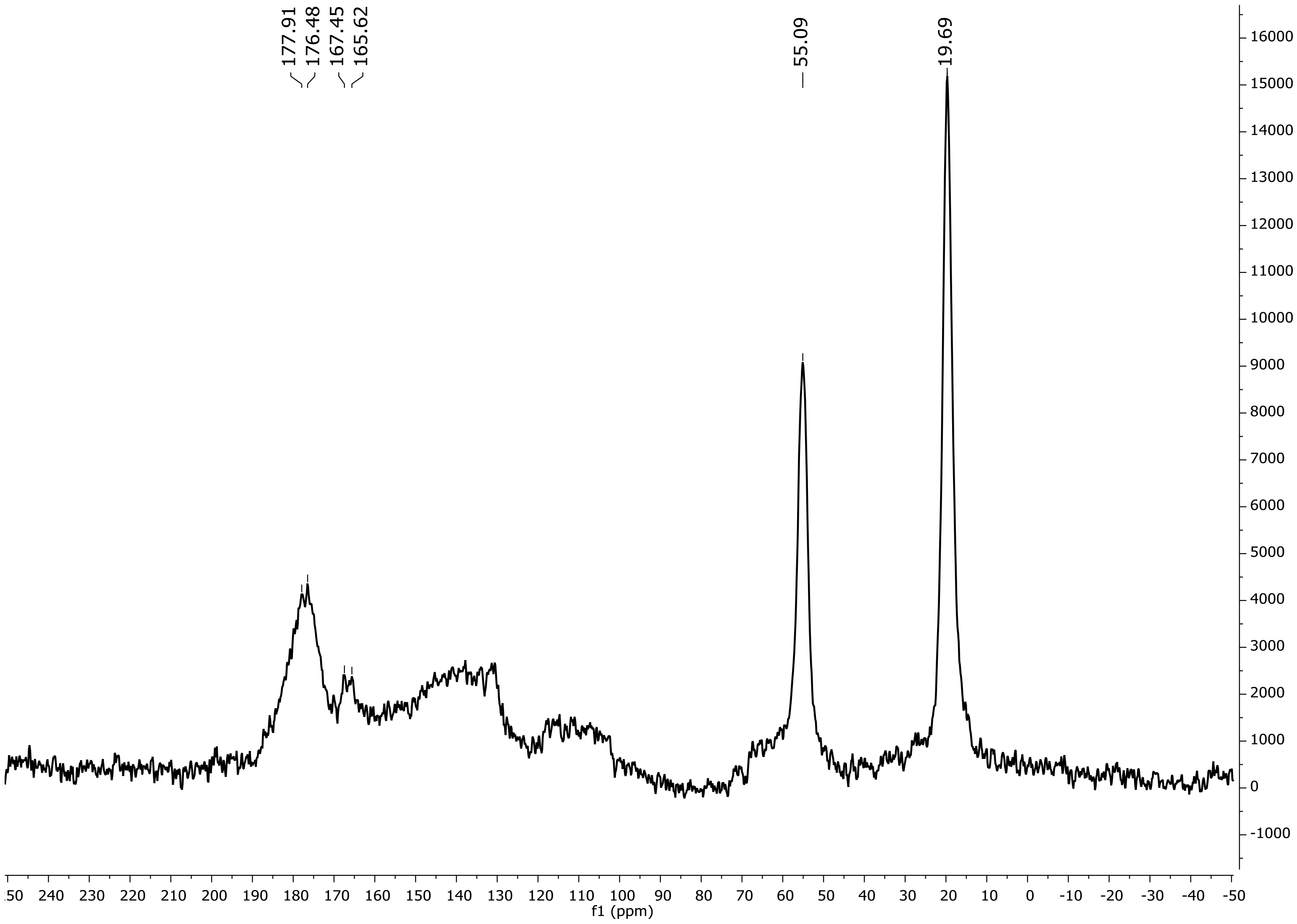

Figura S26. 100 MHz Solid state ^{13}C NMR Spectrum of diacetyl-curcumin.with Mn (II).

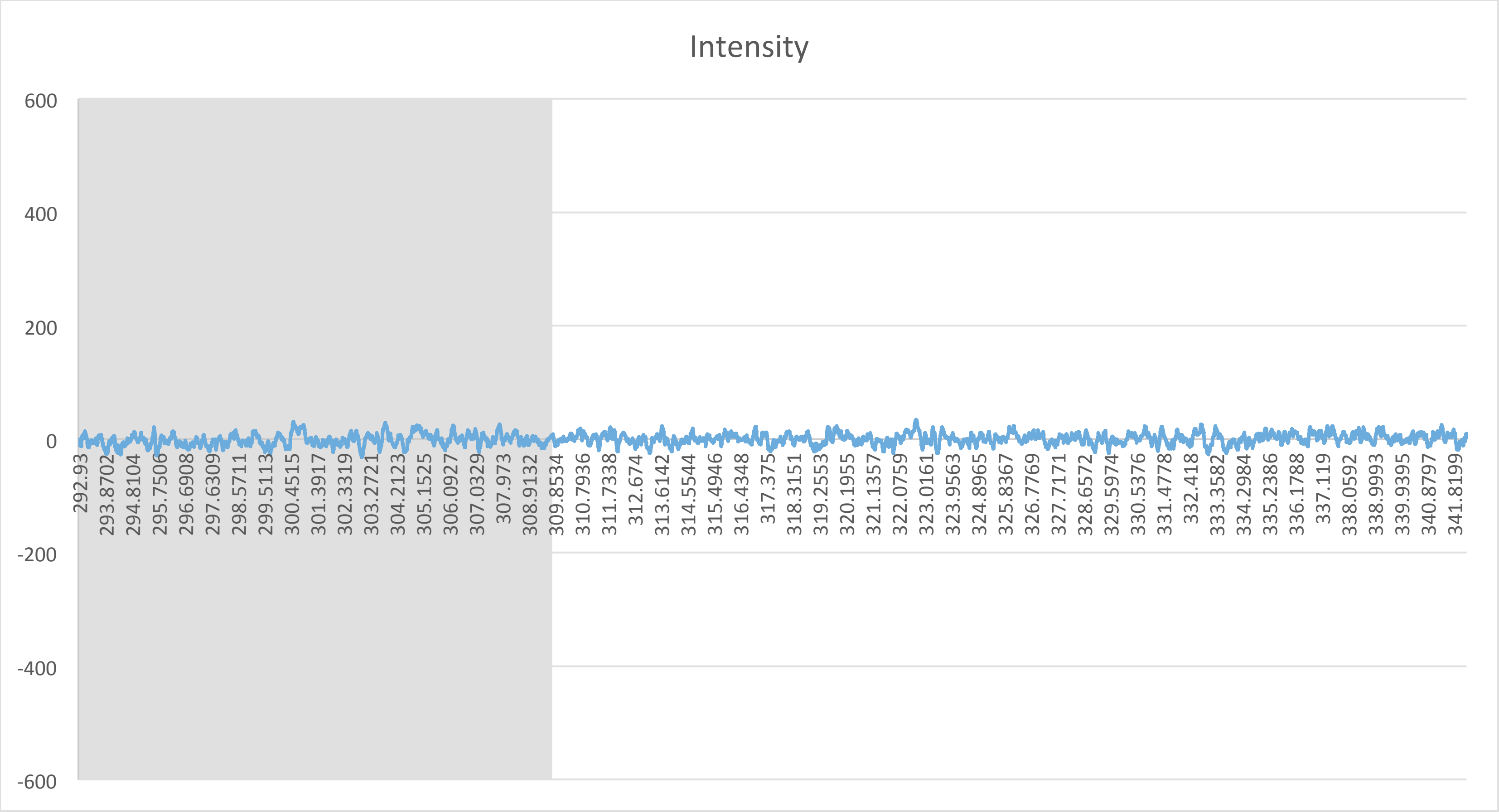

Figura S27. EPR spectrum of diacetyl-curcumin.

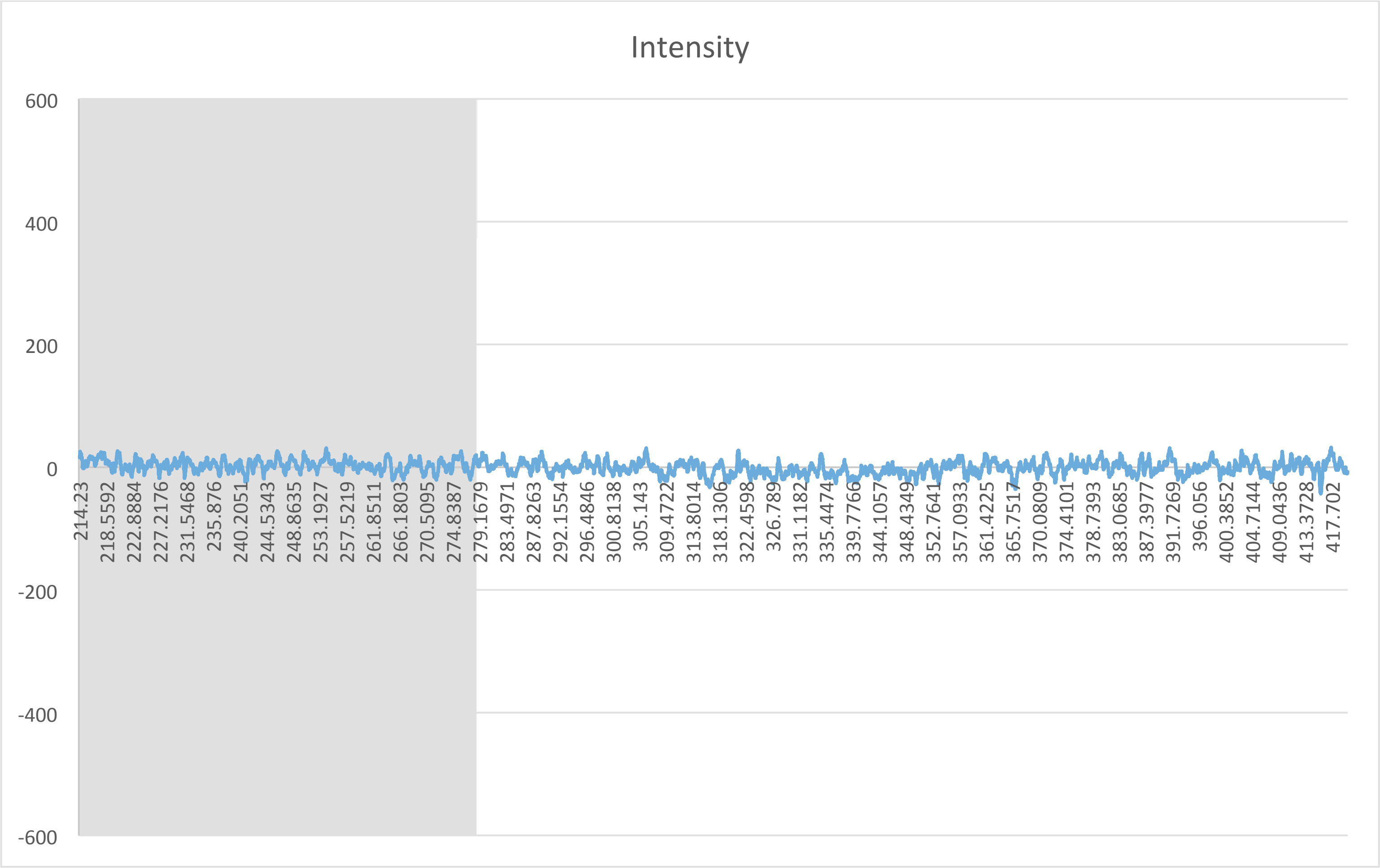

Figura S28. EPR spectrum of diacetyl-curcumin with Mg(II).

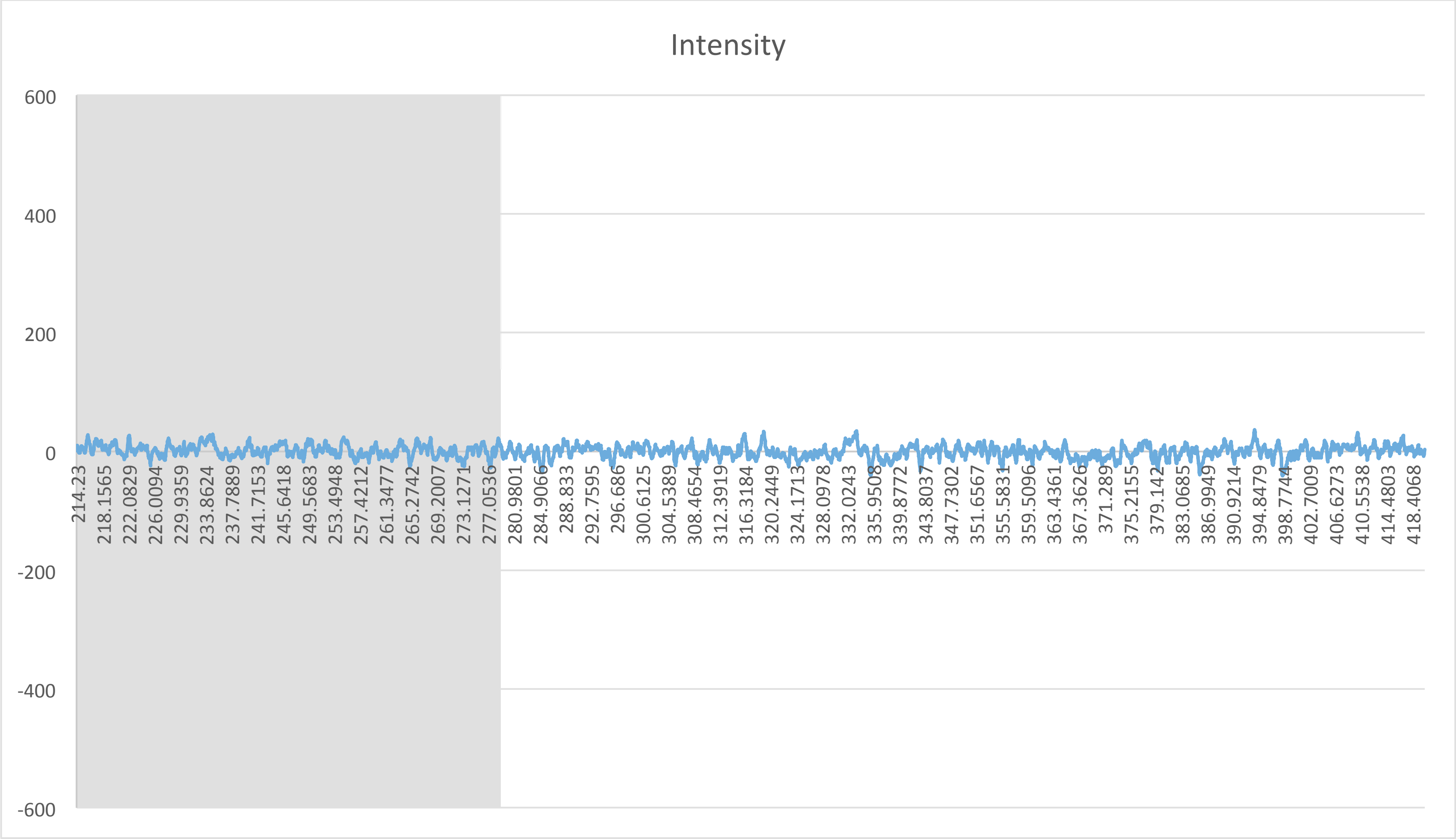

Figura S29. EPR spectrum of diacetyl-curcumin with Zn(II).

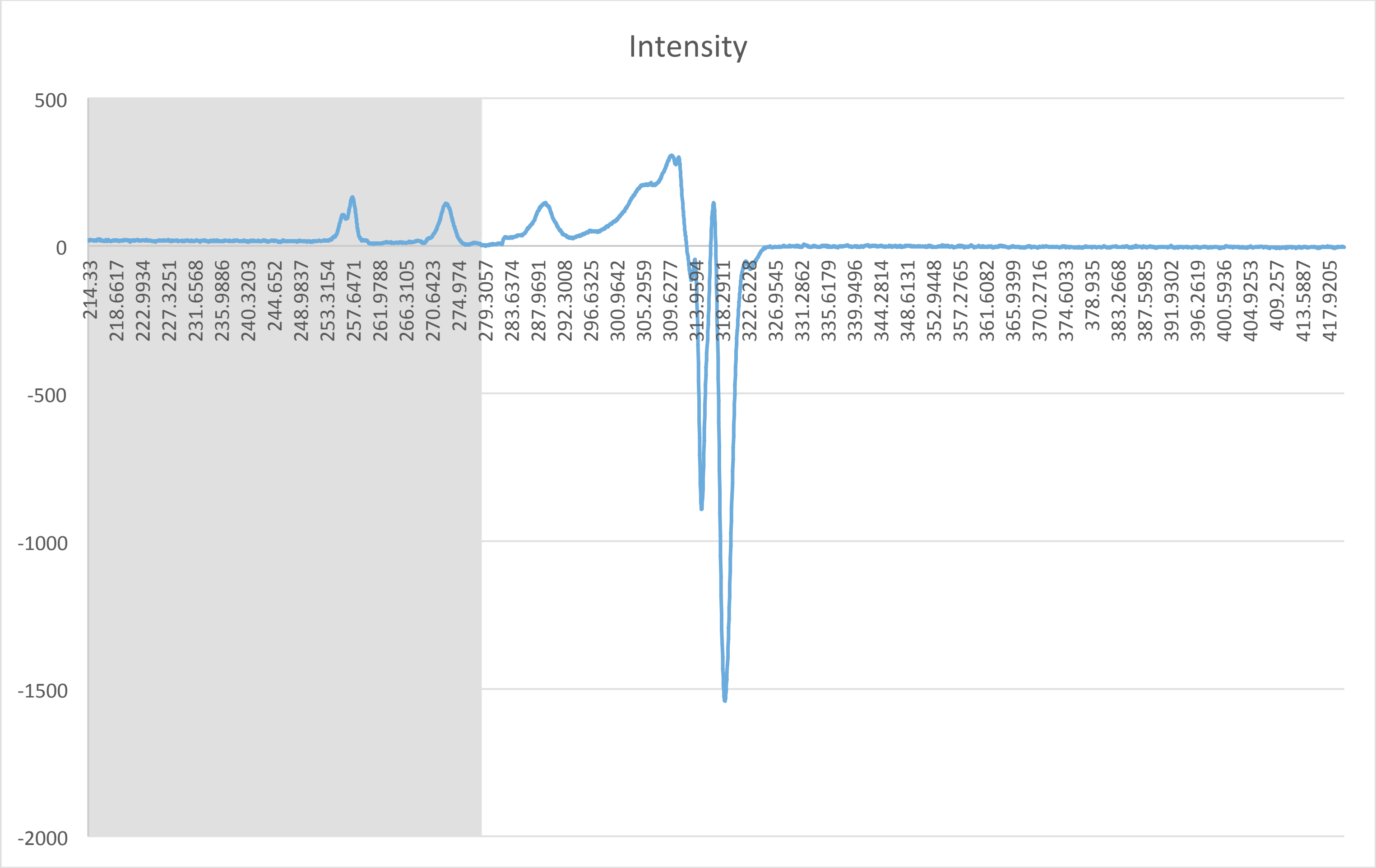

Figura S30. EPR spectrum of diacetyl-curcumin with Cu(II).

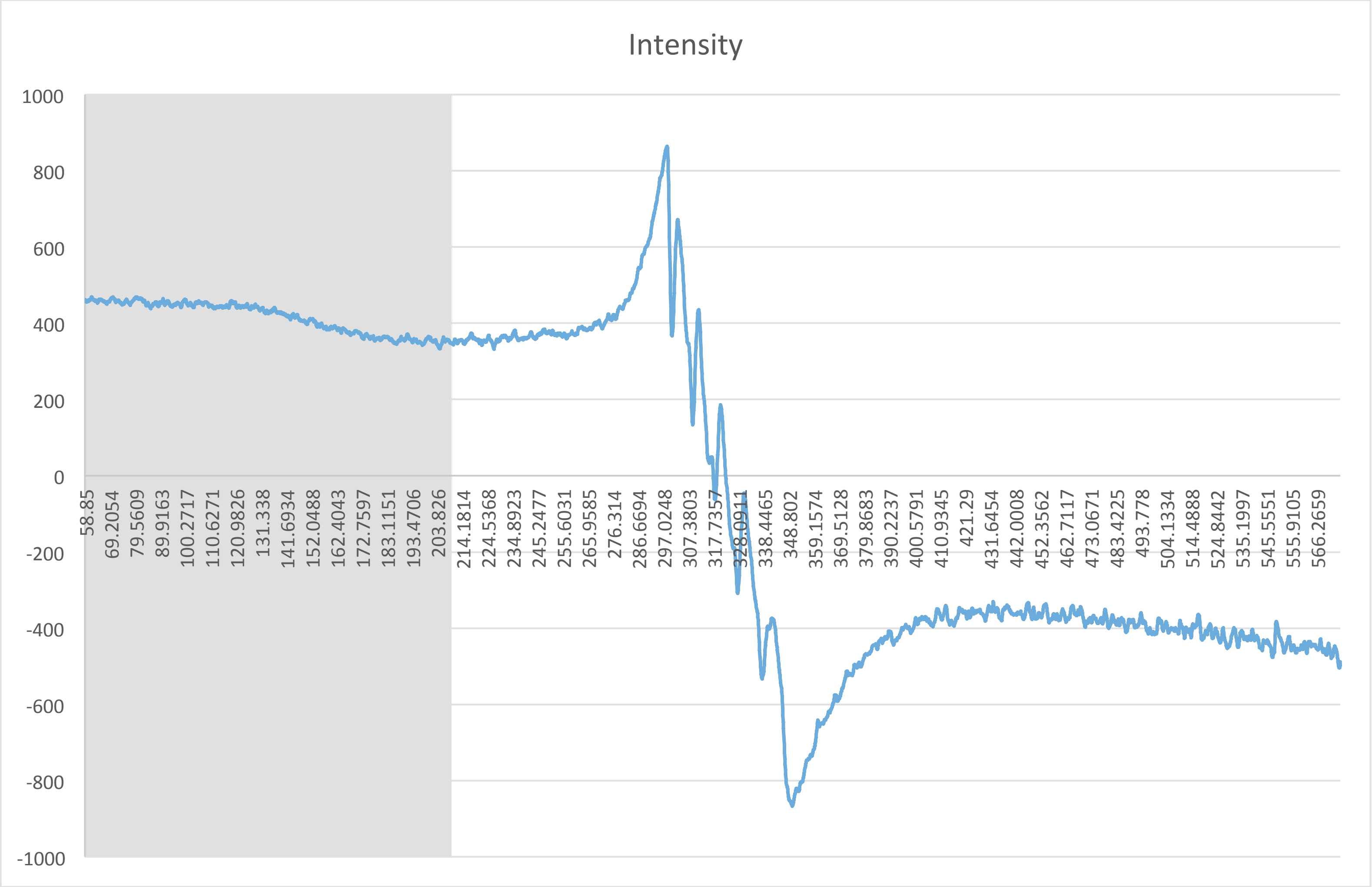

Figura S31. EPR spectrum of diacetyl-curcumin with Mn(II).

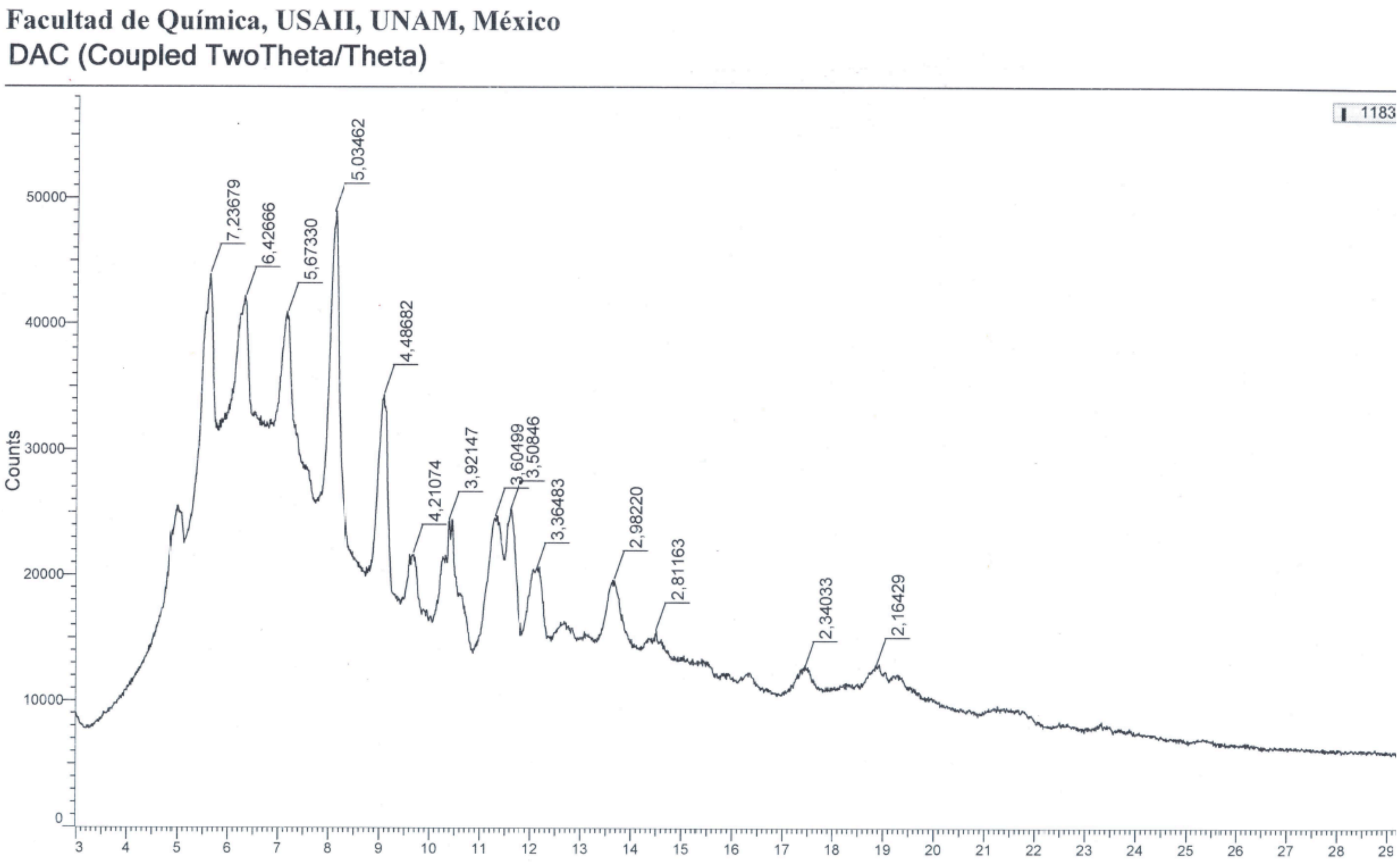

Figura S32. Powder XRD patterns of diacetyl-curcumin.

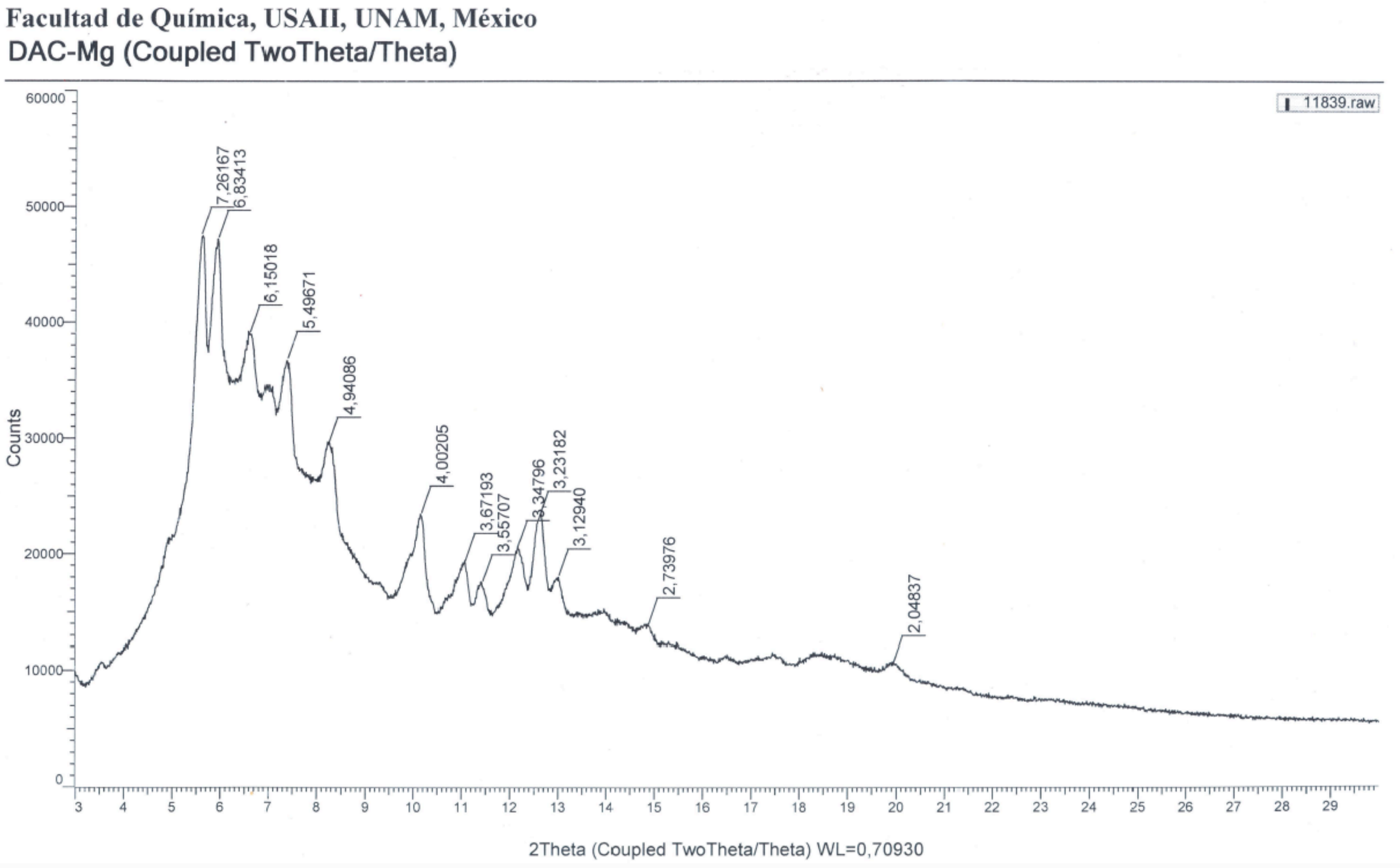

Figura S33. Powder XRD patterns of diacetyl-curcumin with Mg (II).

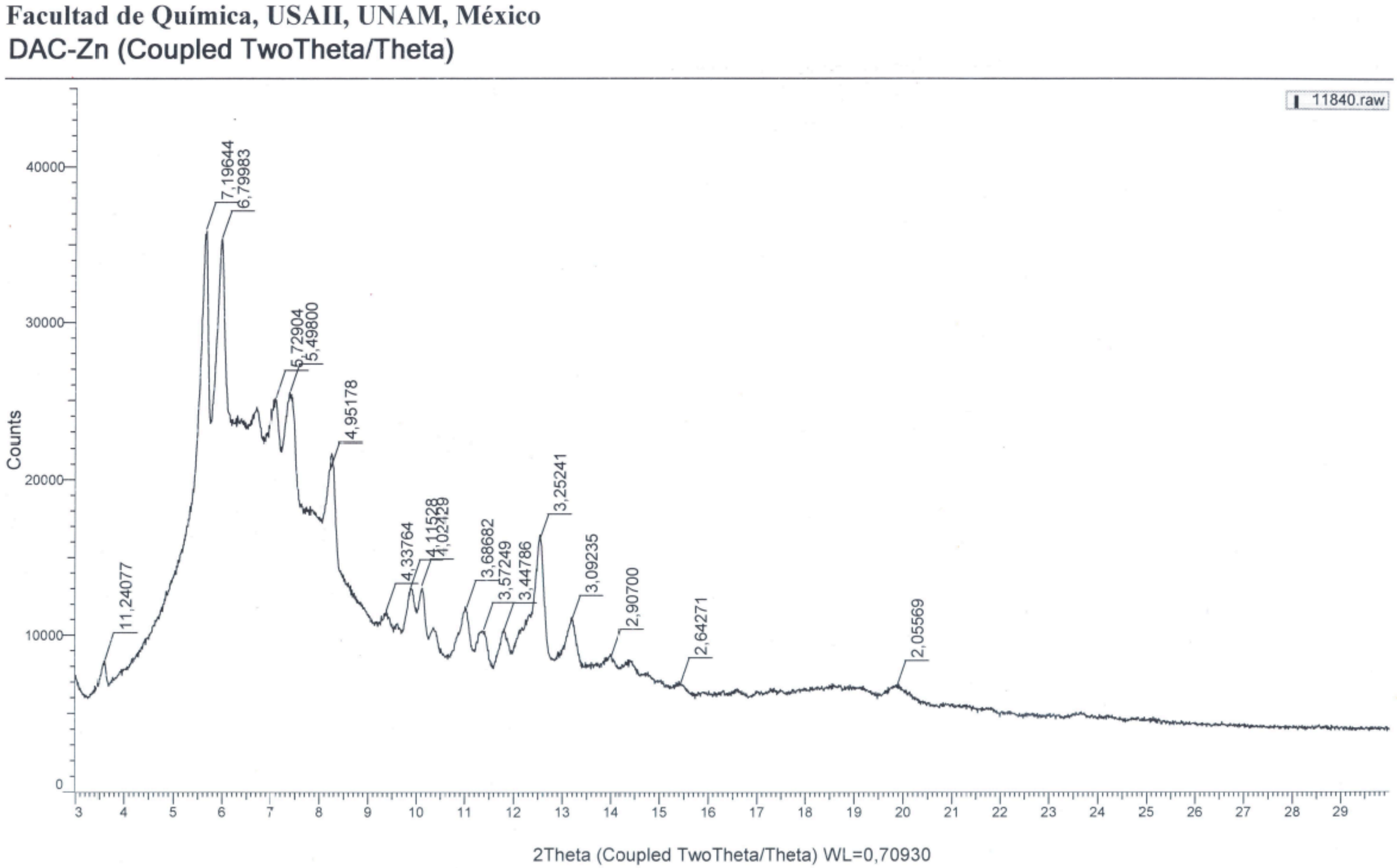

Figura S34. Powder XRD patterns of diacetyl-curcumin with Zn (II).

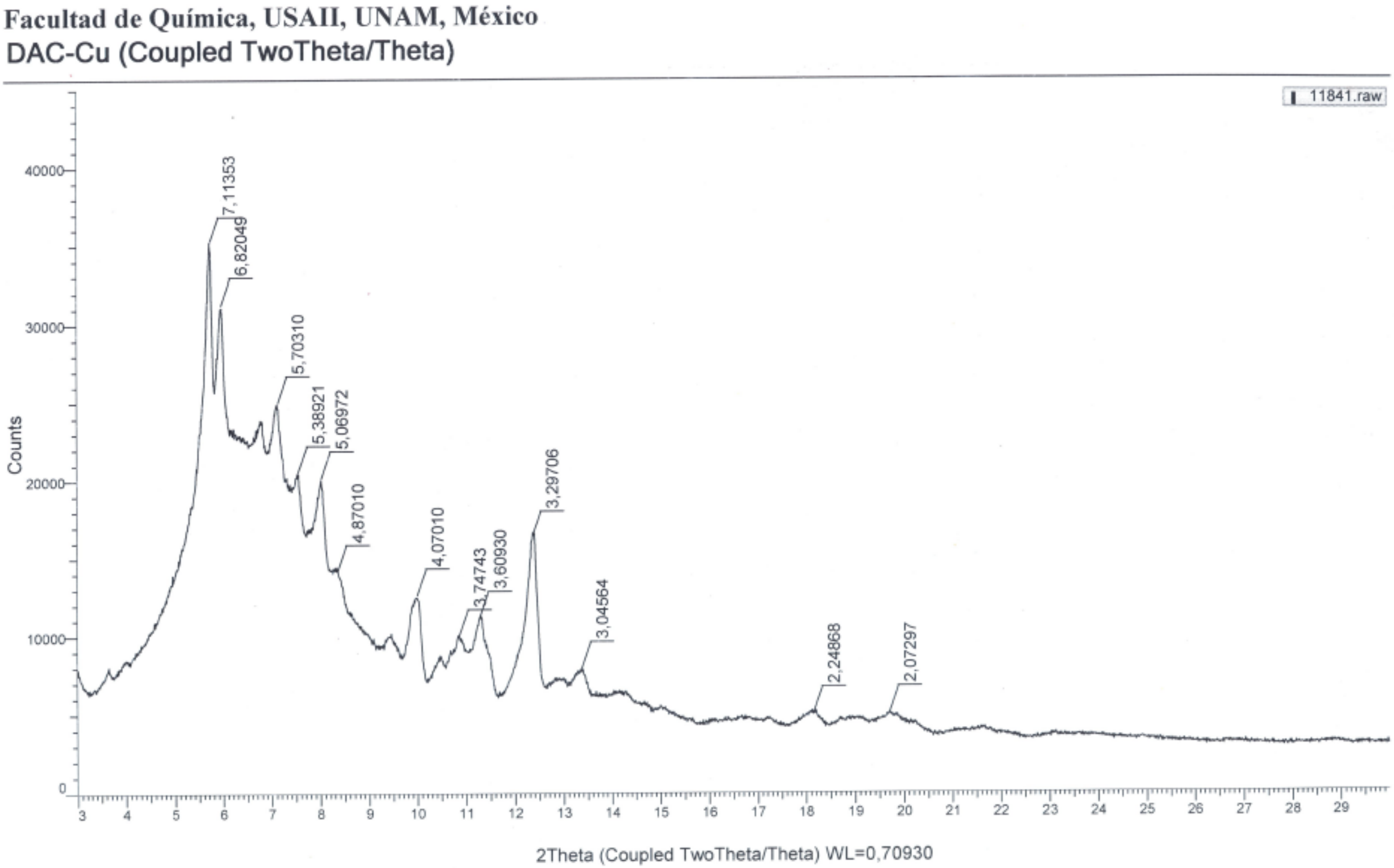

Figura S35. Powder XRD patterns of diacetyl-curcumin with Cu (II).

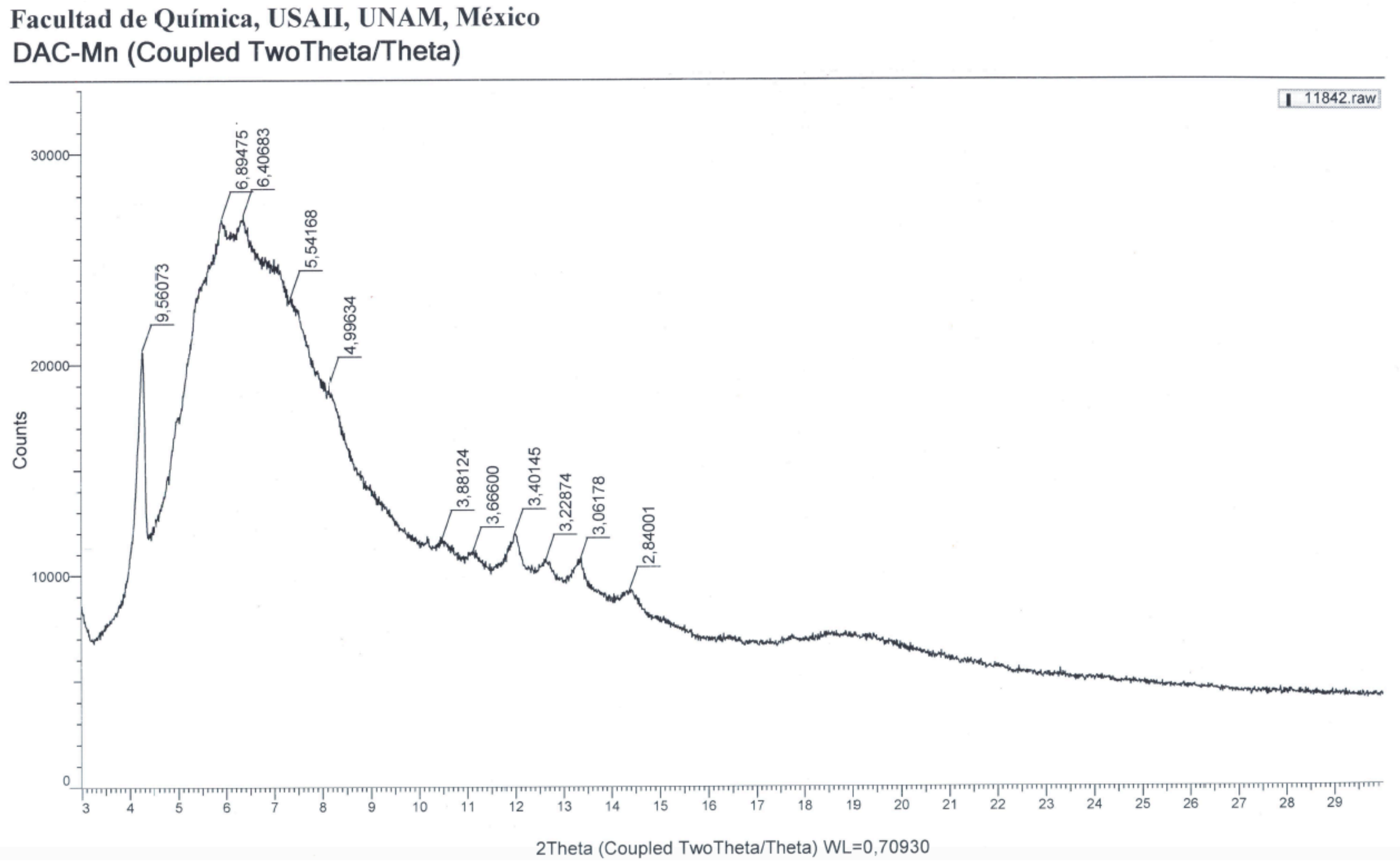

Figura S36. Powder XRD patterns of diacetyl-curcumin with Mn (II).

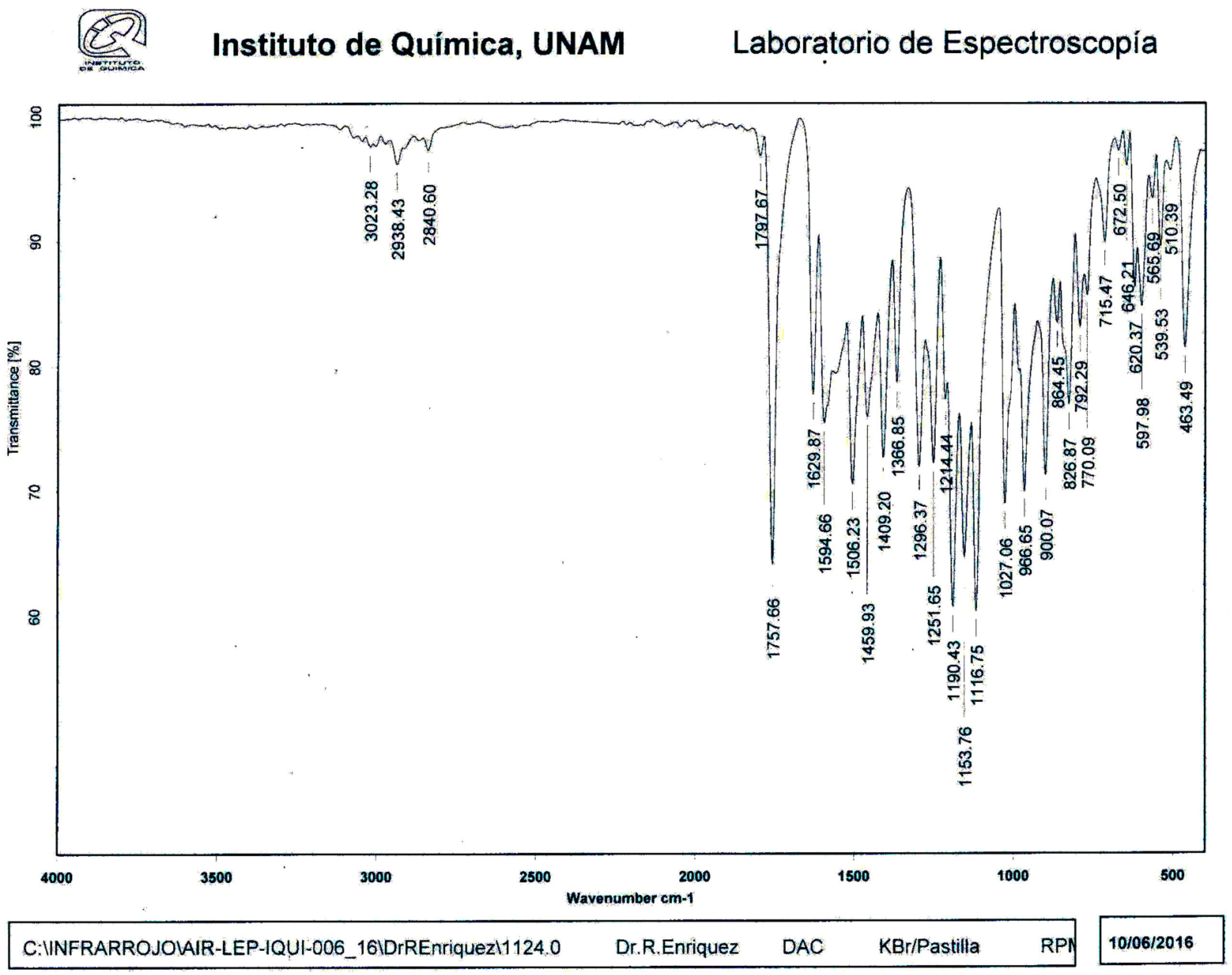

Figure S37. IR Spectrum of diacetyl-curcumin.

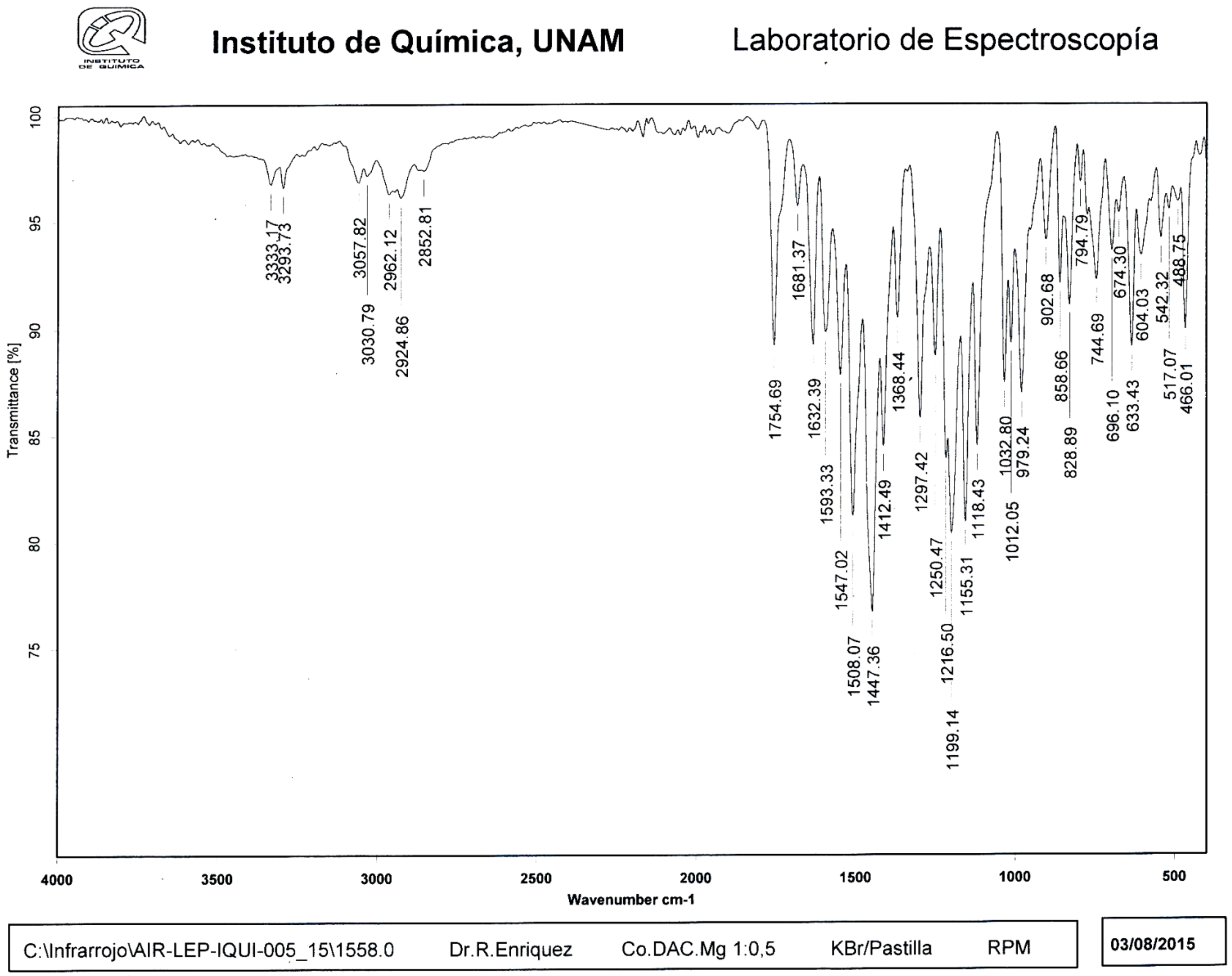

Figure S38. IR Spectrum of diacetyl-curcumin with Mg (II).

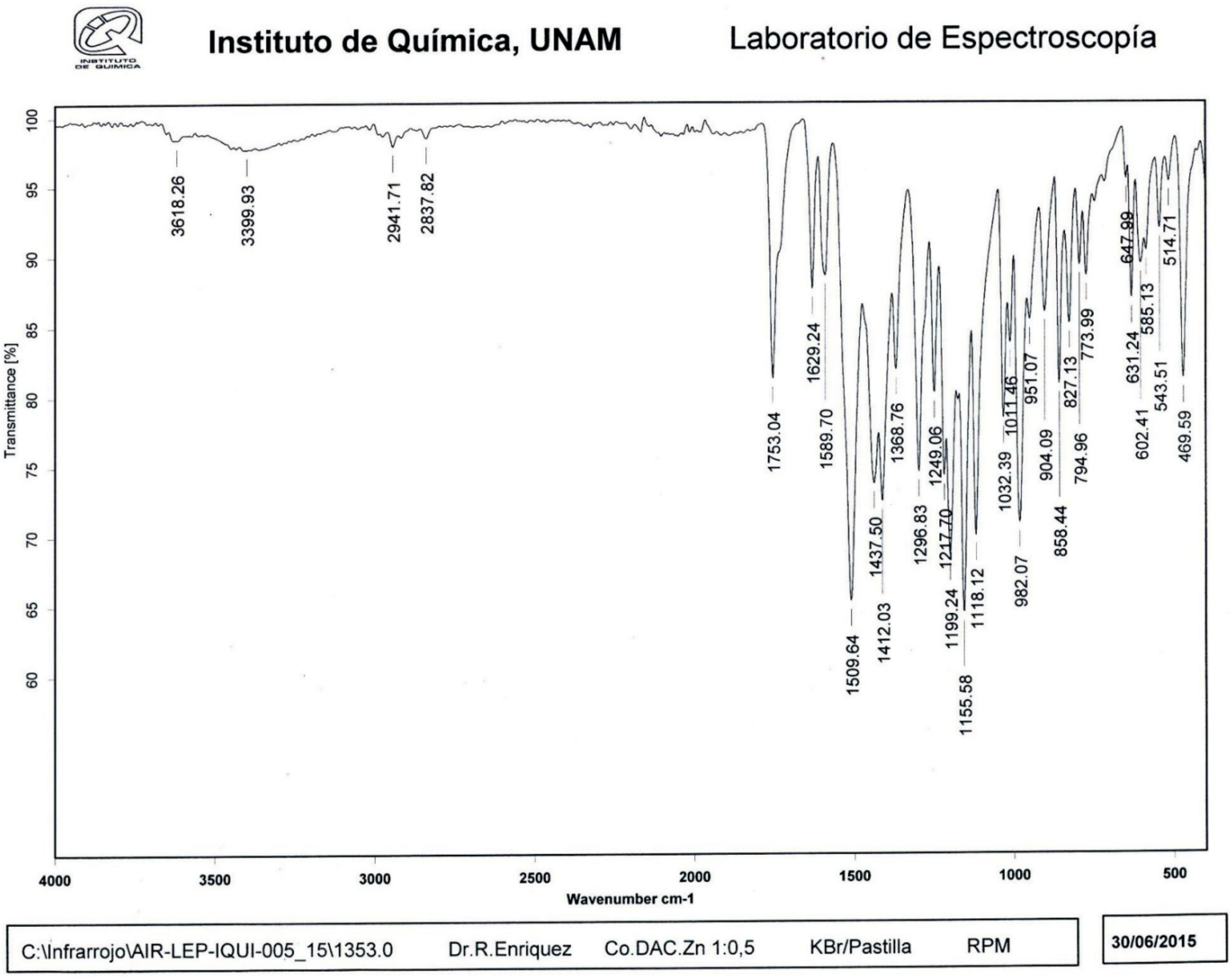

Figure S39. IR Spectrum of diacetyl-curcumin with Zn (II).

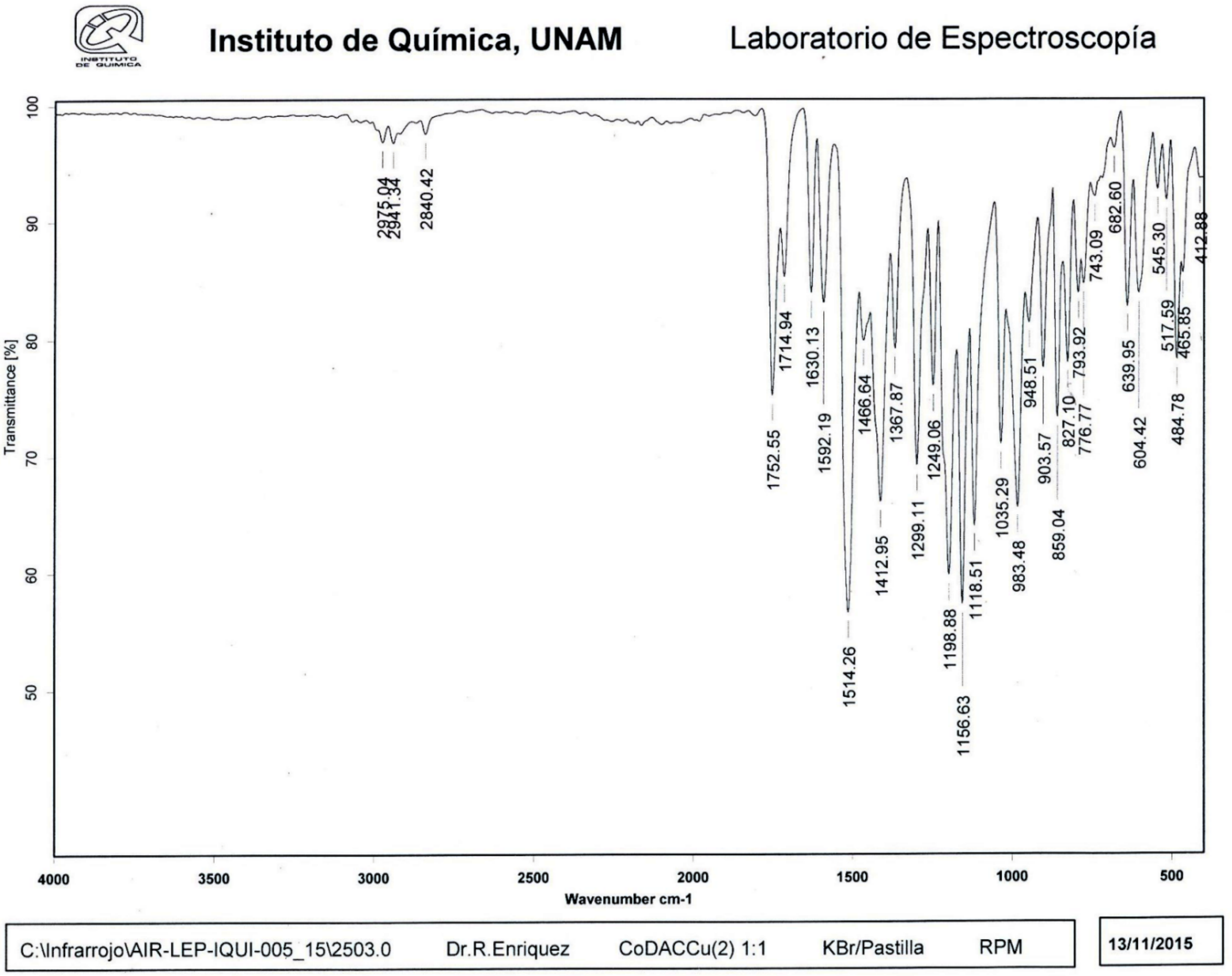

Figure S40. IR Spectrum of diacetyl-curcumin with Cu (II).

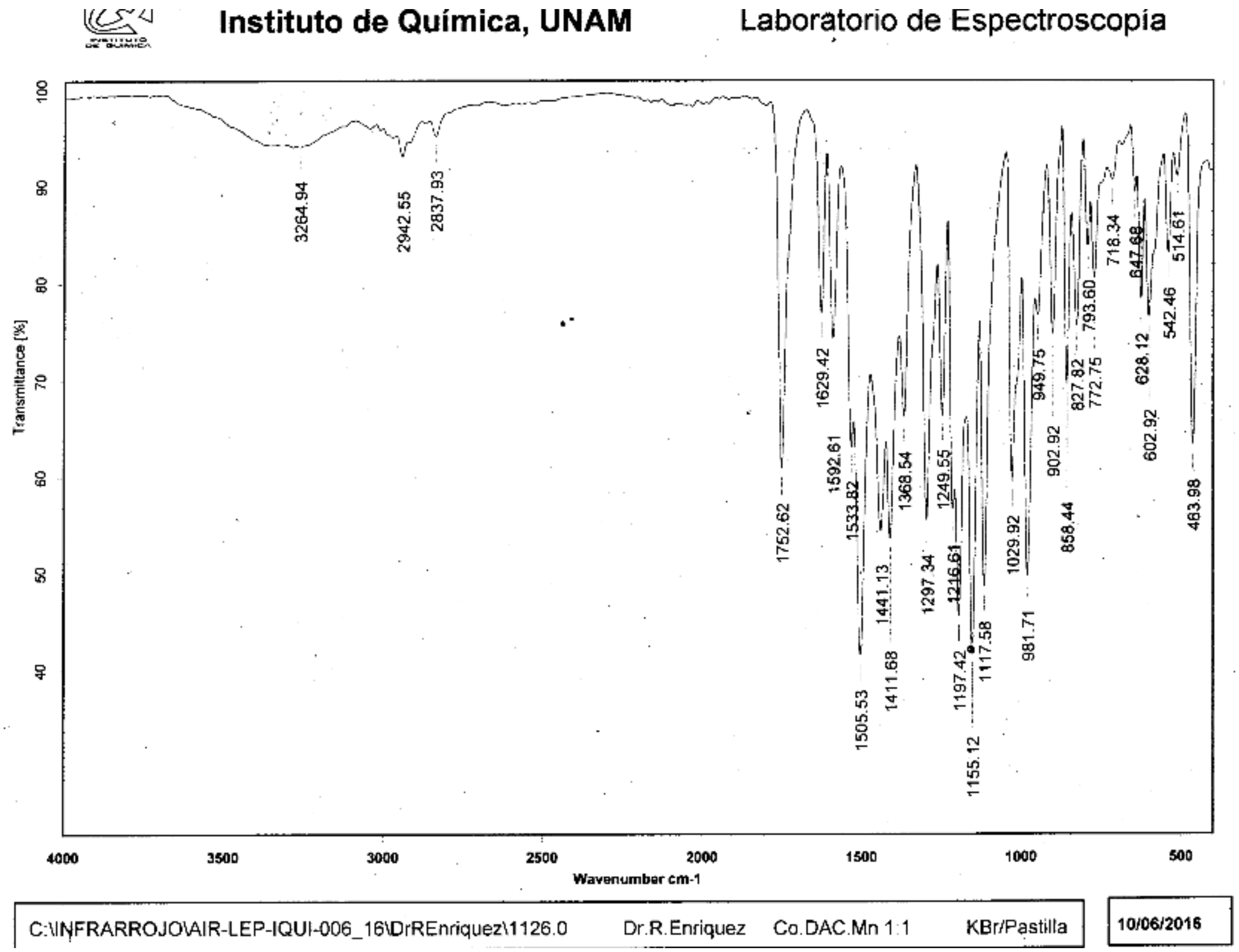

Figure S41. IR Spectrum of diacetyl-curcumin with Mn (II).

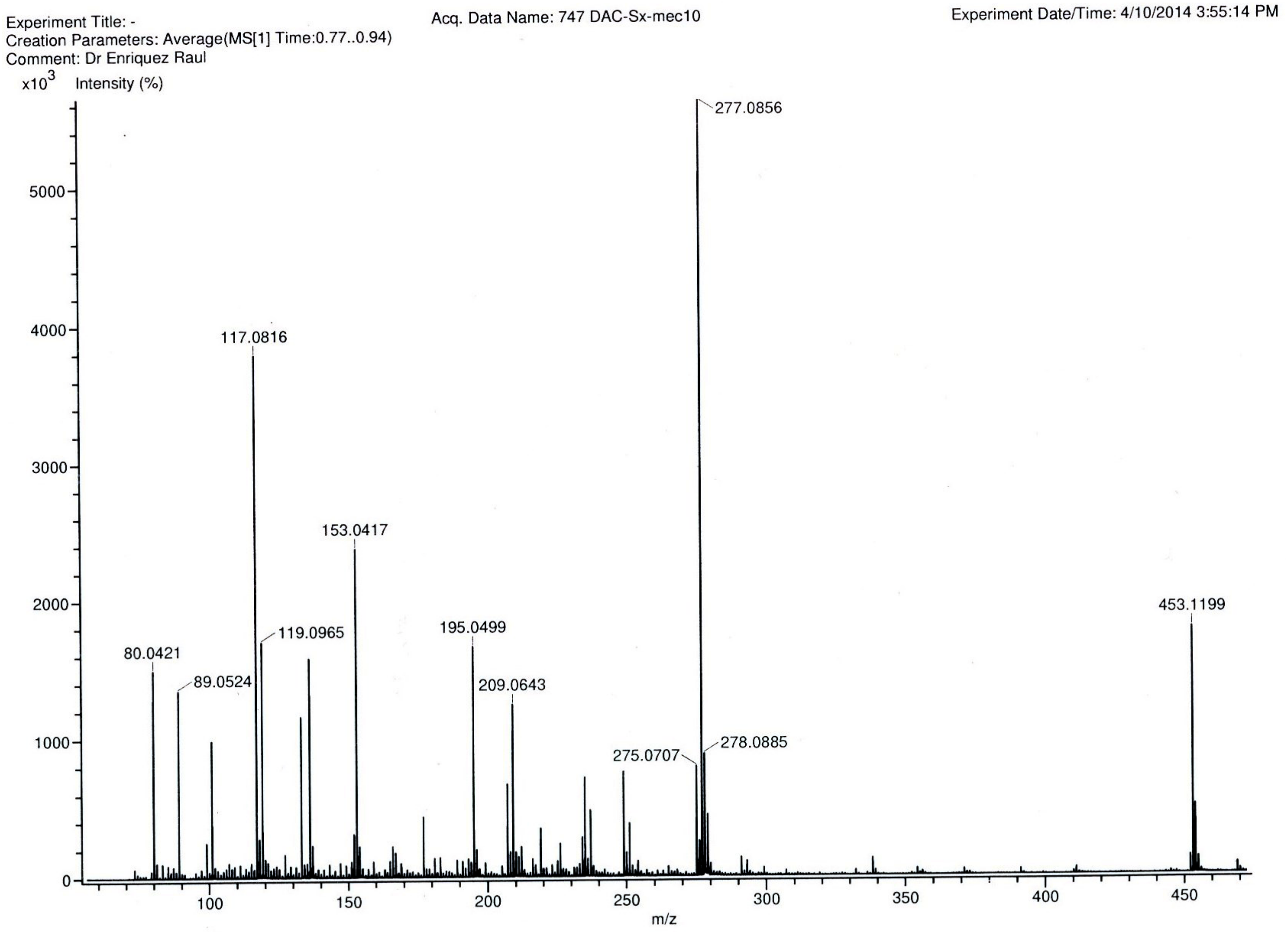

Figura S42. Mass Spectrum of diacetyl-curcumin.

Data : Dr Raul Enriquez011 Date : 28-Sep-2016 18:02
Instrument : MStation
Sample : 3499 DAC
Note : -Luis Velasco
Inlet : Direct Ion Mode : FAB+
RT : 6.18 min Scan# : (41,62)
Elements : C 29/0, H 49/0, O 10/0
Mass Tolerance : 1000ppm, 2mmu if m/z > 2
Unsaturation (U.S.) : -0.5 - 45.0

	Observed m/z	Int%					
	453.1541	46.13					
	Estimated m/z	Err[ppm / mmu]		U.S.	C	H	O
1	453.1549	-1.9 /	-0.8	13.5	25	25	8

Figura S43. Mass Spectrum of mass exact of diacetyl-curcumin.

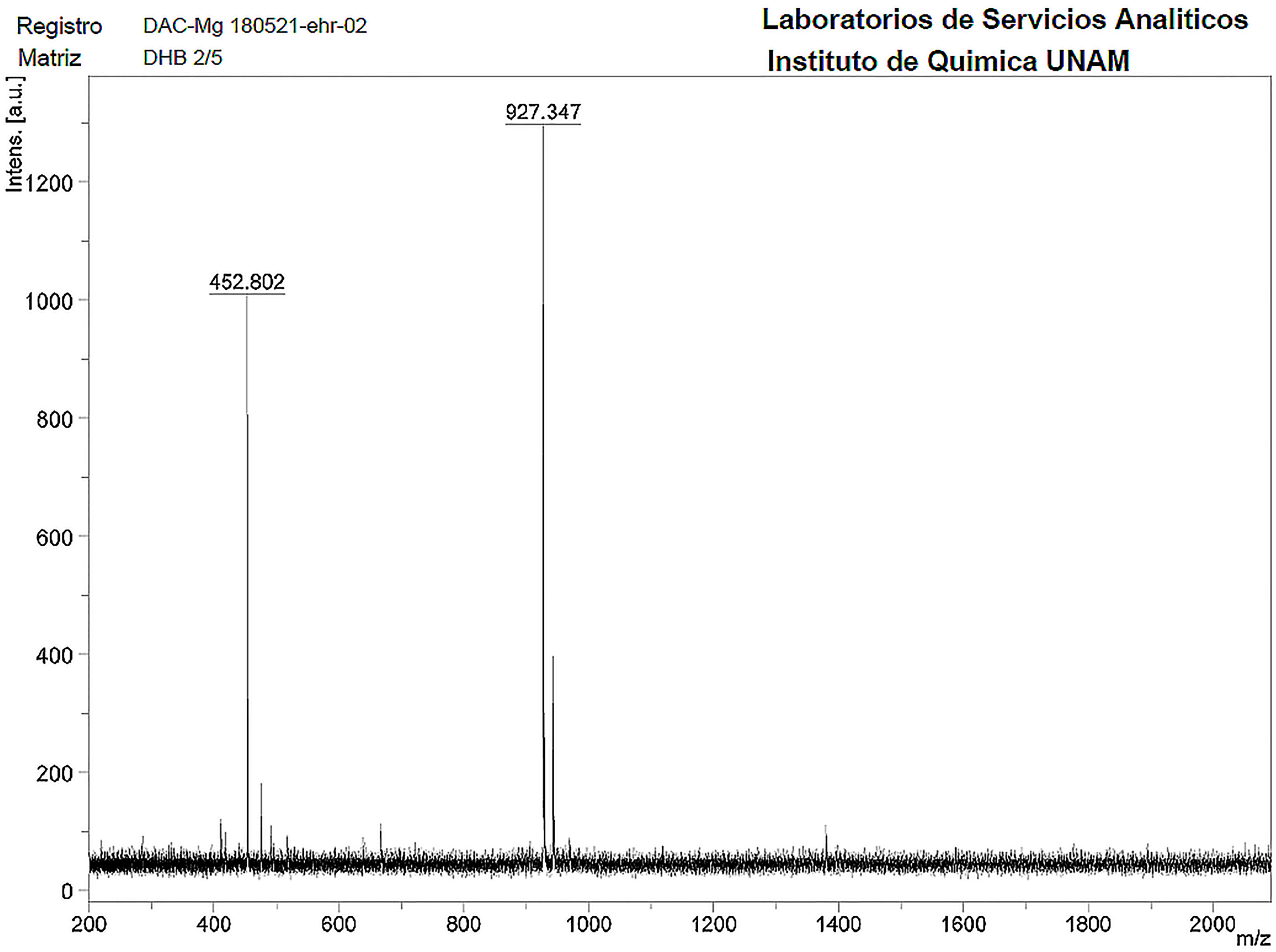

Figura S44. Mass Spectrum of diacetyl-curcumin with Mg(II).

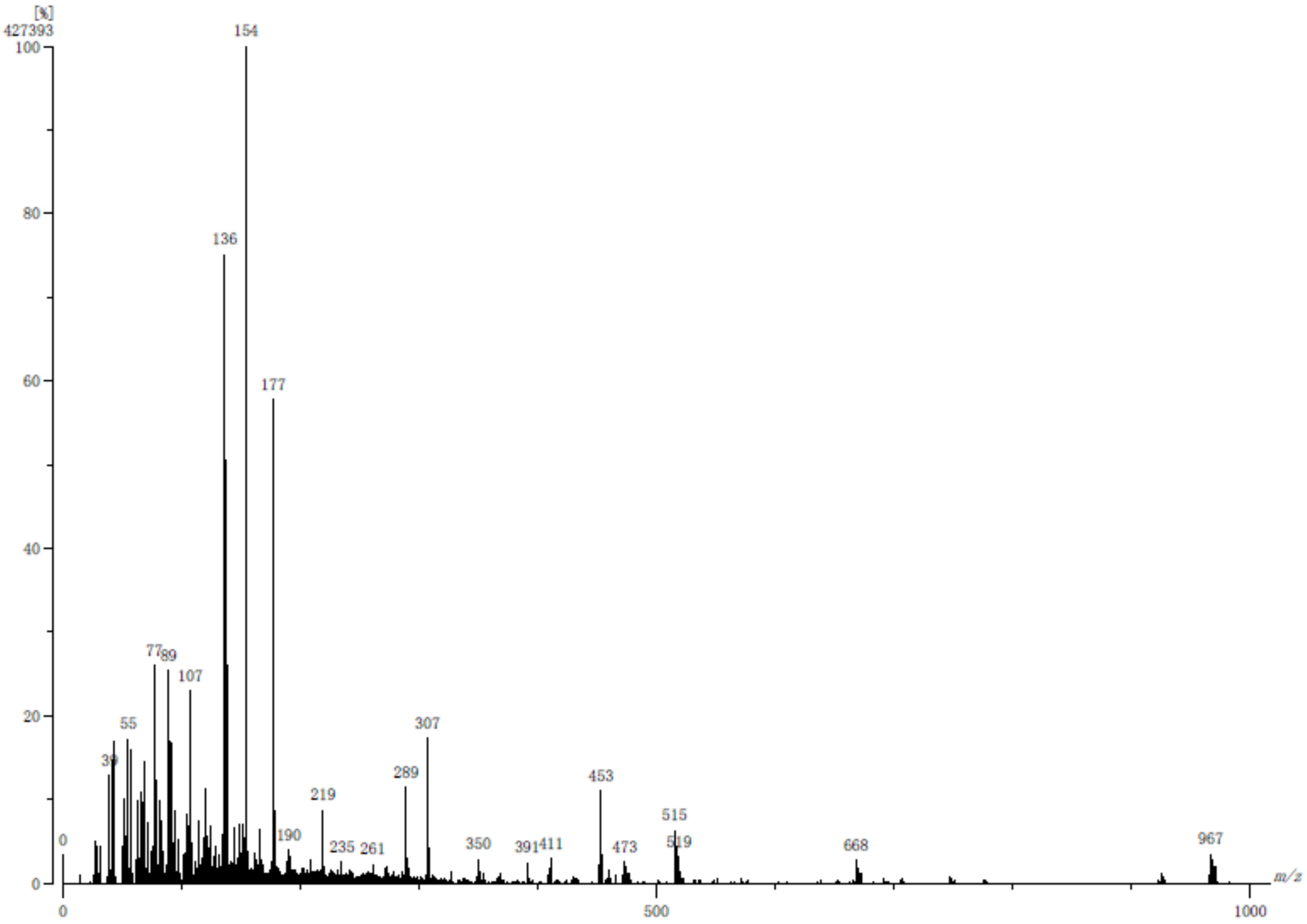

Figura S45. Mass Spectrum of diacetyl-curcumin.with Zn(II).

Data : Dr Raul Enriquez004 Date : 28-Sep-2016 13:34
Instrument : MStation
Sample : 3501 DAC-Zn
Note : -Luis Velasco
Inlet : Direct Ion Mode : FAB+
RT : 9.90 min Scan# : (116,169)+50+(22,44)
Elements : C 55/0, H 49/0, O 18/0, Zn 2/0
Mass Tolerance : 1000ppm, 2mmu if m/z > 2
Unsaturation (U.S.) : -0.5 - 67.0

	Observed m/z	Int%					
	967.2168	85.81					
	Estimated m/z	Err[ppm / mmu]		U.S.	C	H	O
1	967.2156	+1.3 /	+1.2	27.5	50	47	16

Figura S46. Mass Spectrum of mass exact of diacetyl-curcumin.with Zn(II).

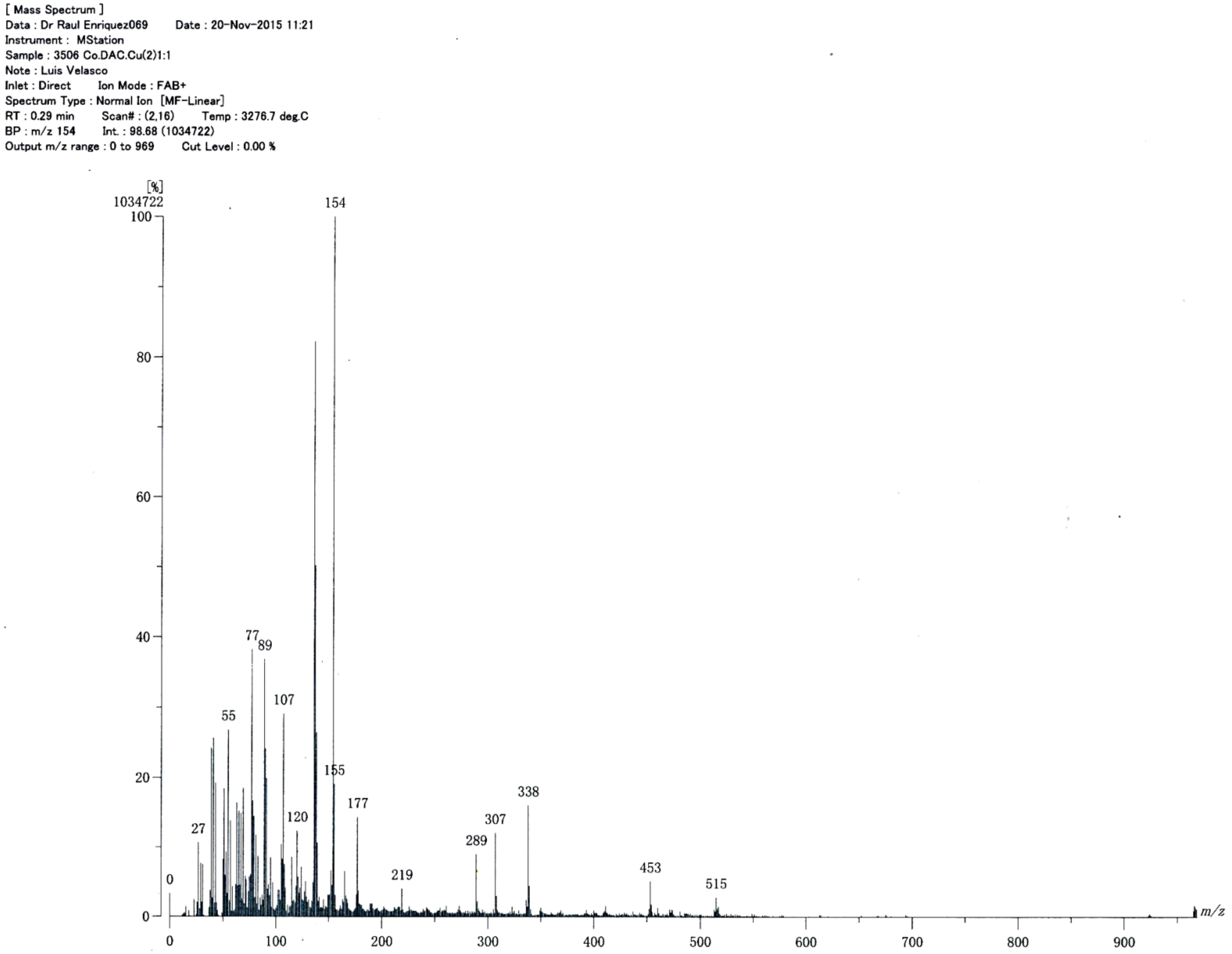

Figura S47. Mass Spectrum of diacetyl-curcumin.with Cu(II).

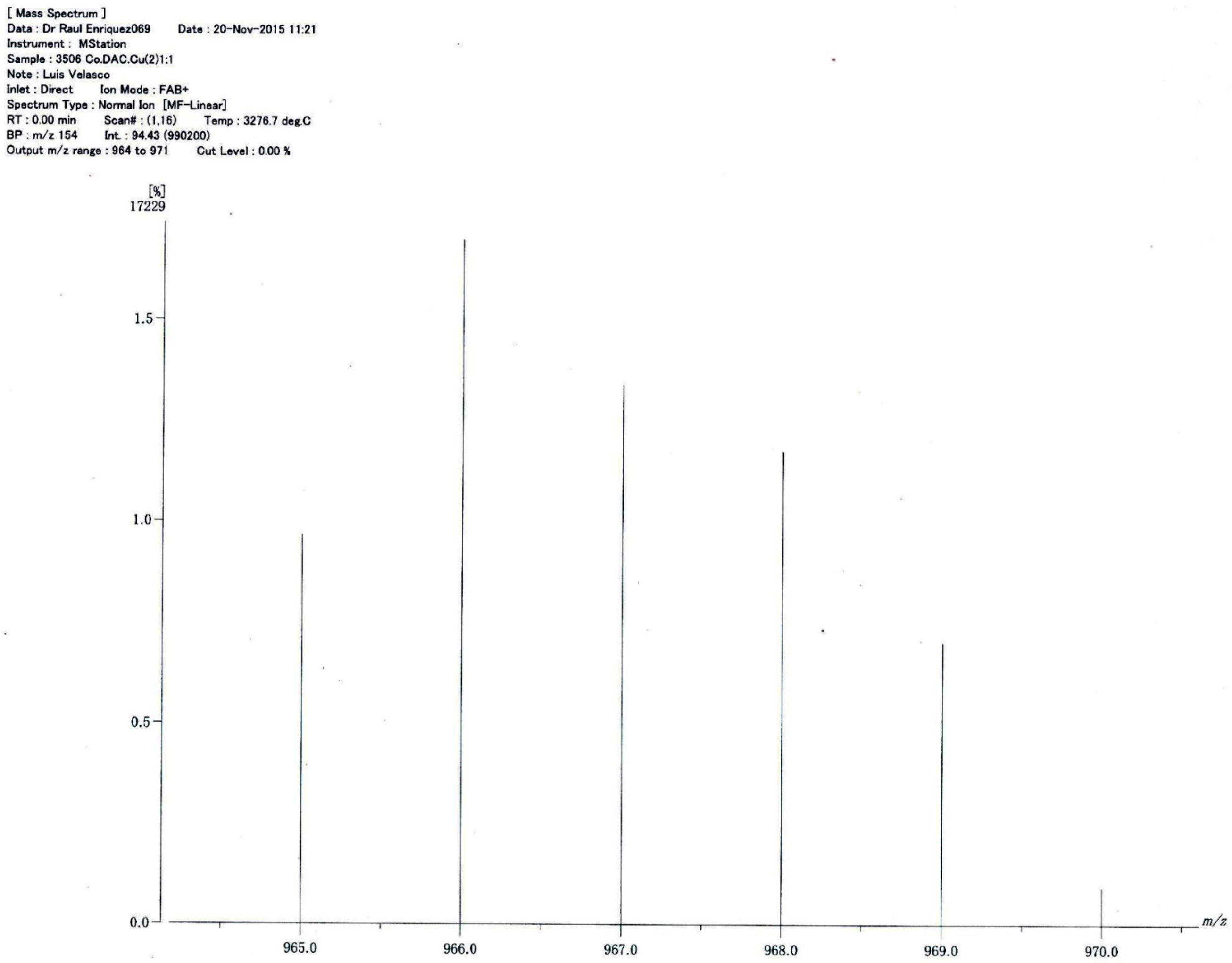

Figura S48. Mass Spectrum expansion of diacetyl-curcumin.with Cu(II).

Data : Dr Raul Enriquez007 Date : 19-Feb-2019 13:21
Instrument : MStation
Sample : 284 DAC-Cu
Note : -Luis Velasco
Inlet : Direct Ion Mode : FAB+
RT : 4.91 min Scan# : (58,134)
Elements : C 54/0, H 49/0, O 17/0, Cu 2/0
Mass Tolerance : 1000ppm, 2mmu if m/z > 2
Unsaturation (U.S.) : 14.0 - 46.0

Observed m/z	Int%
966.2164	100.00

	Estimated m/z	Err[ppm / mmu]	U.S.	C	H	O
1	966.2160	+0.4 / +0.4	27.5	50	47	16

Figura S49. Mass Spectrum of mass exact of diacetyl-curcumin.with Cu(II).

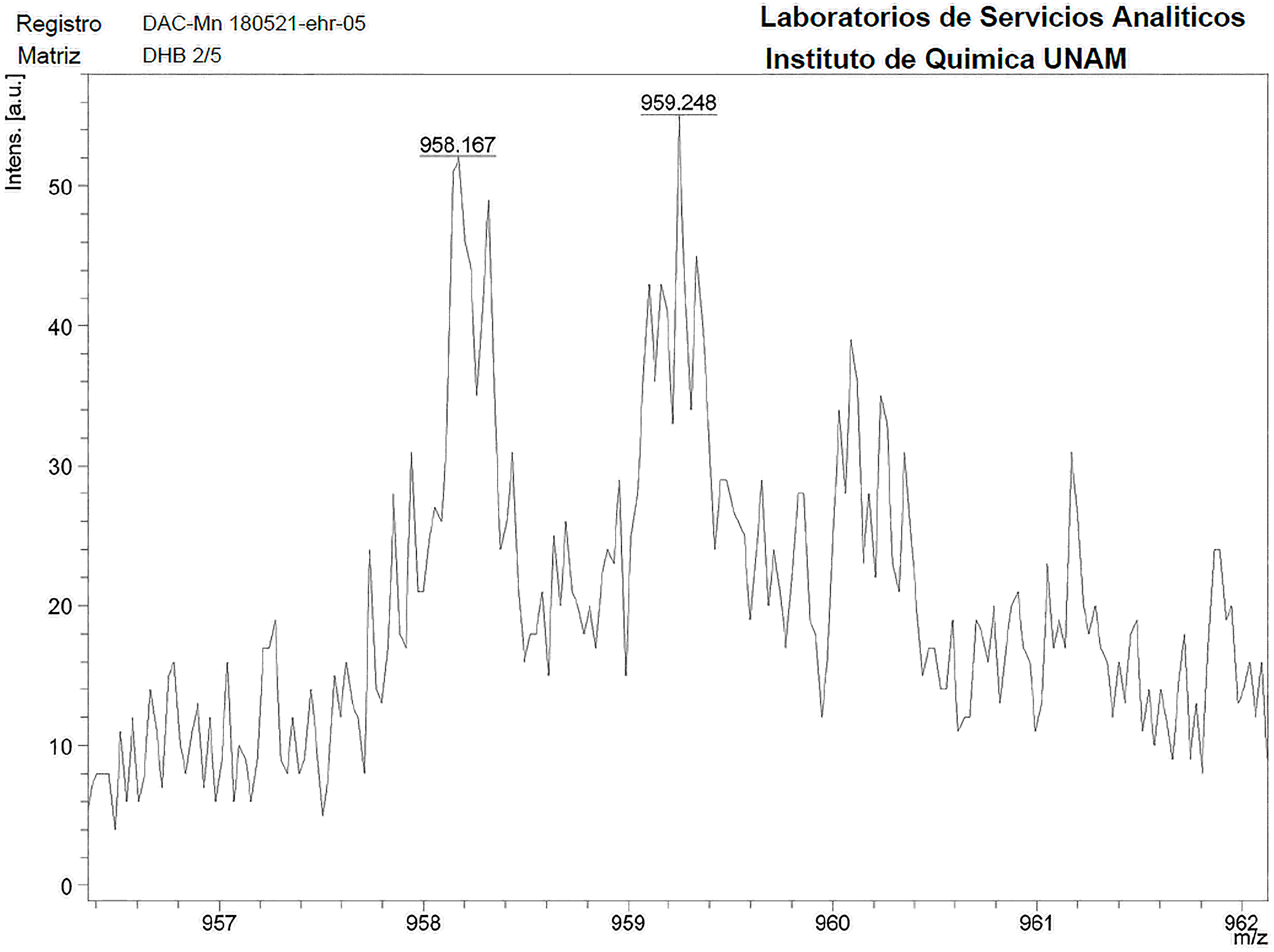

Figura S50. Mass Spectrum expansion of diacetyl-curcumin.with Mn(II).

Table S1. Selected bond distances (Å) and angles (°) for compounds **2, 3**, **4** and **5.**

Compound				
	Mg1 - O1	2.022(3)	Mg1 - O2	2.053(3)
	Mg1 - O9	2.125(3)	O1 - C3	1.270(5)
	O2 - C5	1.266(5)	C1 - C2	1.307(6)
**2**	C1 - C8	1.463(6)	C2 - C3	1.485(6)
	C3 - C4	1.397(6)	C4 - C5	1.411(6)
	O1 - Mg1 - O2	92.4(1)	O1 - C3 - C4	125.1(4)
	C3 - C4 - C5	125.7(4)	O2 - C5 - C4	123.6(4)
	O1 - Mg1 - O9	90.47(12)	O2 - Mg1 - O9	91.52(12)
	Zn1 - O2	1.987(2)	Zn1 - O10	1.991(2)
	Zn1 - O17	2.014(4)	Zn1 - O1	2.021(2)
	Zn1 - O9	2.031(2)	O1 - C3	1.273(4)
	O2 - C5	1.267(4)	O9 - C28	1.270(4)
	O10 - C30	1.272(4)	C3 - C4	1.396(5)
	C4 - C5	1.402(5)	C28 - C29	1.394(5)
	C29 - C30	1.400(5)		
**3**	O2 - Zn1 - O10	151.80(13)	O2 - Zn1 - O17	103.40(15)
	O10 - Zn1 - O17	104.75(15)	O2 - Zn1 - O1	89.50(10)
	O10 - Zn1 - O1	85.92(10)	O17 - Zn1 - O1	96.24(13)
	O2 - Zn1 - O9	86.52(10)	O10 - Zn1 - O9	88.97(10)
	O17 - Zn1 - O9	102.48(14)	O1 - Zn1 - O9	161.27(12)
	C3 - O1 - Zn1	125.6(2)	C5 - O2 - Zn1	125.1(2)
	C28 - O9 - Zn1	126.6(2)	C30 - O10 - Zn1	127.3(2)
	C3 - C4 - C5	125.0(3)	C28 - C29 - C30	125.4(3)
	Cu1 - O2	1.900(4)	Cu1 - O1	1.906(4)
	Cu1 - O10	1.913(4)	Cu1 - O9	1.916(4)
	O1 - C3	1.281(6)	O2 - C5	1.282(7)
	O9 - C28	1.277(7)	O10 - C30	1.279(7)
	C3 - C4	1.392(7)	C4 - C5	1.398(7)
	C28 - C29	1.392(7)	C29 - C30	1.384(7)
	O2 - Cu1 - O1	94.44(16)	O2 - Cu1 - O10	87.08(15)
**4**	O1 - Cu1 - O10	175.24(18)	O2 - Cu1 - O9	174.24(18)
	O1 - Cu1 - O9	85.75(16)	O10 - Cu1 - O9	93.20(16)
	C3 - O1 - Cu1	125.0(3)	C5 - O2 - Cu1	125.2(3)
	C28 - O9 - Cu1	125.8(3)	C30 - O10 - Cu1	125.4(3)
	O1 - C3 - C4	125.2(5)	C3 - C4 - C5	124.1(5)
	O2 - C5 - C4	125.2(5)	C28 - O9 - Cu1	125.8(3)
	C30 - O10 - Cu1	125.4(3)	O9 - C28 - C29	125.4(5)
	C30 - C29 - C28	123.4(6)	C30 - O10 - Cu1	125.4(3)
	Mn01 - O1	2.108(4)	Mn01 - O2	2.132(4)
	Mn01 - O9	2.243(4)	O1 - C3	1.270(5)
	O2 - C5	1.262(5)	C1 - C2	1.317(7)
	C1 - C8	1.464(6)	C2 - C3	1.484(7)
**5**	C3 - C4	1.395(6)	C4 - C5	1.397(7)
	O1 - Mg1 - O2	92.4(1)	O1 - C3 - C4	125.1(4)

C3	- C4	- C5	125.7(4)	O2	- C5	- C4	123.6(4)
O1	- Mn01-	O2	84.82(15)	O1	- Mn01 -	O9	88.09(15)
O2	- Mn01-	O9	88.49(16)	O1	- C3	- C4	126.3(4)
C3	- C4	- C5	125.8(4)	O2	- C5	- C4	124.8(4)

The asymmetric unit of **2** contains one molecule of DAC, one Mg(II) cation and one DMSO molecule. The Mg(II) cation is located on inversion center (½, 0, ½). It is located in a distorted octahedral coordination by four oxygen atoms from two β-diketonate ligands, and two oxygen atoms from two DMSO molecules.

In **3**, the asymmetric unit consists of one Zn(II) cation, two DAC molecules and one DMSO molecule. The Zn(II) cation is located in a distorted square pyramidal coordination by four oxygen atoms from two β-diketonate ligands, and one oxygen atom of a DMSO molecule.

In **4**, the asymmetric unit consists of one Cu(II) cation, two DAC molecules and one DMF molecule. The Cu(II) cation is located in a distorted square planar coordination by four oxygen atoms from two β-diketonate ligands. The DMF molecule does not participate in the metal coordination.

The asymmetric unit of **5** contains one Mn(II) cation, one molecule of DAC and one DMSO molecule. The Mn(II) cation is located in a distorted octahedral coordination by four oxygen atoms from two β-diketonate ligands and two oxygen atoms from two DMSO molecules.

The distance between the Cg centers of gravity for phenyl rings of compounds **2** and **5** are: C8 to C13 and C17 to C22, 12.39 and 12.40 Å, respectively. For compounds **3** and **4** the distances from C8 to C13 and C17 to C22; C33 to C38 and C42 to C47 are: 12.33, 12.34; 12.35 12.24 Å, respectively.

Selected dihedral angles for compounds **2**, **3**, **4** and **5** are given in Table **S2**. In compound **2**, **3**, **4** and **5**, the dihedral angles between the C8 to C13; C17 to C22 phenyl rings and the central moiety (C6-C1 to C7-C17) are: 19.4(2), 11.9(2), 33.3(3); 18.0(2), 17.6(2), 8.9(2)°, respectively. The dihedral angles between C8-to C13; C17 to C22 phenyl rings and the six-membered ring C3-C4-C5-O2-M-O1 for compounds **2**, .**3** and **4** with values of 5.8(1), 21.4(1), 38.7(2); 31.9(1), 8.0(7), 10.78(2)°, respectively [10,32].

Table **S3**, shows the dihedral angles between the phenyl rings and the corresponding acetoxy groups in compounds **2**, **3**, **4** and **5**. Major deviations occur in **4**. In all four compounds we conclude that the molecular conformation of the acetoxy groups is adjusted to accommodate the coordination requirements of each metal.

In **2**, the packing diagram is given Fig. **S51**. The molecules forming the crystal pack are continuous layers of DAC along the *c* axis. DMSO molecules and methoxy and acetoxy groups at C10 and C20, fulfill the spaces in between these layers. The dihedral angle

between both aromatic rings in **2** is 36.8(2)°. Bond lengths are: Mg1-O1, Mg1-O2, and Mg1-O9 are 2.022(3), 2.053(3) and 2.125(3) Å, respectively. The bond angle of O1-Mg1-O2 is 92.4(1)°. The coordination polyhedron about the magnesium atom is a distorted octahedron.

Packing diagram for **3** is given in Fig **S52**. The molecules in the crystal pack in such a way that the DAC molecules are lying parallel to the *b* axis forming continuous layers. Layers of DMSO molecules separate the layers of DAC. The dihedral angle between both aromatic rings in compound **3** is 42.0(2)°. Bond lengths are: Zn1-O1, Zn1-O2, Zn1-O9 and Zn1-O10 are 2.021(2), 1.987(2), 2.031(2) and 1.991(2) Å. Bond angles are: O1-Zn1-O2, O2-Zn1-O9, O9-Zn1-10, O10-Zn1-O1, O2-Zn1-O10, O1-Zn1-O9, O1-Zn1-O17, O2-Zn1-O17, O9-Zn1-17, O10-Zn1-O17, and are 89.5(1), 86.5(2), 88.9(1)°, 85.9(2), 151.8(1), 161.27(12), 96.2(1), 103.4(2), 102.48(14) and 104.7(2), respectively. The polyhedron about the zinc atom has a deformed square pyramidal arrangement.

In **4**, a perspective drawing of the packing arrangement of DAC-Cu molecules along the *b* direction is given in Fig. **S53**. The DAC molecules in the crystal pack are adjusted to accommodate the distortion of the DAC in the deviated portion part (aromatic ring, C8 to C13) and DMF molecules in the crystal. The dihedral angle between both aromatic rings in **4** is 36.8(2)°. The lengths of Cu-O bond of Cu1-O1, Cu1-O2, Cu1-O9 and Cu1-O10 are 1.906(4), 1.900(4), 1.916(4) and 1.913(4) Å, respectively, and are similar to those reported previously [10] while these values are rather different respect to other diketonate complexes with copper [33, 34]. The bond angles are: O2-Cu1-O1, O2-Cu1-O10, O1-Cu1-O10, O2-Cu1-O9, O1-Cu1-O9 and O10-Cu1-O9 are 94.4(2), 87.1(2), 175.2(2), 174.2(2), 85.8(2) and 93.2(2)°, respectively. The coordination renders a planar square geometry.

In **5**, the packing diagram is given Fig. **S54**. The molecules in the crystal pack are in a diagonal along the *ab* plane forming continuous layers. The spaces between these layers are fulfilled by DMSO molecules and the acetoxyl radical at C20 (aromatic ring, C17 to C22) DAC molecules. The dihedral angle between both phenyl rings in **5** is 34.5(2)°. Bond lengths are: Mn01-O1, Mn01-O2, and Mn01-O9 are 2.108(4), 2.132(4), and 2.243(4) Å, respectively: The bond angle of O1-Mn01-O2 is 92.4(1)°. The coordination polyhedron about the magnesium atom is a distorted octahedron.

In all four compounds **2**, **3**, **4** and **5**, the packing of the molecules in the crystal structures is stabilized by van der Waals interactions and linked by intermolecular C-H···O hydrogen interactions. The molecular geometry of these intermolecular interactions is given in Table **S4**. The adjacent molecules are interconnected by intermolecular weak C-H···π interactions (from different Cg centers of gravity), which give additional support to molecular packing stability. In **2**, the (C4-H4···Cg (phenyl ring, C17 to C22) and C14-H14A···Cg (phenyl ring, C8 to C13) with donor and acceptor distances and angles of 3.63, 3.69 Å, 122.7, 144.0°, respectively. In **3**, the (C14-H14A···Cg (phenyl ring, C42 to C47); (C23-H23A···Cg (phenyl ring, C17 to C22); (C16-H16A···Cg (phenyl ring, C17 to C22); and (C16-H16C···Cg (phenyl ring, C17 to C22) with donor and acceptor distances and angles of 3.62,

3.64, 3.63 Å; 157.2, 132.9, 146.0°, respectively. In **4**, the (C4-H4···Cg (phenyl ring, C42 to C47); (C25-H25A···Cg (phenyl ring, C33 to C38); (C39-H39C···Cg (phenyl ring, C33 to C38); and (C14-H14A···Cg (phenyl ring, C8 to C13) with donor and acceptor distances and angles of 3.50, 3.60, 3.55 Å, 102.0, 145.1, 144.7°, respectively. In compound **5**, interaction are observed at C4-H4···Cg (phenyl ring, C17 to C22) and C14-H14C···Cg (phenyl ring, C8 to C13) with donor and acceptor distances and angles of 3.63, 3.63 Å, 118.6, 147.3°, respectively.

Table S2. Selected dihedral angles (°) for compounds **2**, **3, 4** and **5**.

	Compounds			
**Atoms**	**2**	**3**	**4**	**5**
C8-C9-C10-C11-C12-C13<br>C17-C18-C19-C20-C21-C22	36.8(2)	29.1(1)	42.0(2)	34.6(2)
C8-C9-C10-C11-C12-C13<br>C8-C1-C2-C3-C4-C5-C6-C7-C17	19.4(2)	11.9(2)	33.3(3)	18.7(3)
C8-C9-C10-C11-C12-C13<br>C3-C4-C5-O2-Mg1-O1	5.8(1)			
C8-C9-C10-C11-C12-C13<br>C3-C4-C5-O2-Zn1-O1		21.4(1)		
C8-C9-C10-C11-C12-C13<br>C3-C4-C5-O2-Cu1-O1			38.7(2)	
C8-C9-C10-C11-C12-C13<br>C3-C4-C5-O2-Mn01-O1				7.2(2)
C17-C18-19-C20-C21-C22<br>C8-C1-C2-C3-C4-C5-C6-C7-C17	18.0(2)	17.6(2)	8.9(2)	16.5(2)
C17-C18-19-C20-C21-C22<br>C3-C4-C5-O2-Mg1-O1	31.9(1)			
C17-C18-C19-C20-C21-C22<br>C3-C4-C5-O2-Zn1-O1		8.0(7)		
C17-C18-C19-C20-C21-C22<br>C3-C4-C5-O2-Cu1-O1			10.7(2)	
C17-C18-19-C20-C21-C22<br>C3-C4-C5-O2-Mn01-O1				28.8(1)
C33-C34-C35-C36-C37-C38<br>C42-C43-C44-C45-C46-C47		29.3(1)	4.2(2)	
C33-C34-C35-C36-C37-C38<br>C33-C26-C27-C28-C29-C30-C31-C32-C42		9.7(2)	10.6(2)	
C33-C34-C35-C36-C37-C38<br>C3-C4-C5-O2-Zn1-O1		25.1(1)		
C33-C34-C35-C36-C37-C38<br>C3-C4-C5-O2-Cu1-O1			4.9(2)	
C42-C43-C44-C45-C46-C47<br>C42-C26-C27-C28-C29-C30-C31-C32-C33		14.4(2)	11.1(2)	
C42-C43-C44-C45-C46-C47<br>C28-C29-C30-O10-Zn1-O9		4.3(1)		

C42-C43-C44-C45-C46-C47	1.9(2)
C28-C29-C30-O10-Cu1-O9	

Table S3. Dihedral angles (º) for acetoxy groups in compounds **2**, **3, 4** and **5**.

Atoms	2	3	4	5
C8-C9-C10-C11-C12-C13<br>C11-O4-C15(O5)-C16	85.2(2)	89.4(2)	73.5(2)	87.1(2)
C17-C18-C19-C20-C21-C22<br>C20-O7-C24(O8)-C25	61.2(2)	64.0(1)	34.6(2)	79.2(2)
C11-O4-C15(O5)-C16<br>C20-O7-C24-(O8)-C25	67.1(2)	61.5(2)	88.2(2)	66.5(3)
C33-C34-C35-C36-C37-C38<br>C36-O12-C40(O13)-C41		87,7(2)	75.4(2)	
C42-C43-C44-C45-C46-C47<br>C45-O15-C49(O16)-C50		76.8(1)	77.3(2)	
C36-O12-C40(O13)-C41<br>C45-O15-C49(O16)-C50		61.4(2)	50.4(2)	

Table S4. Geometry of the hydrogen bonding interactions (Å, °) for compounds **2**, **3**, **4** and **5**.

Compound	D-H · · · *A*	D-H	H · · · *A*	D · · · *A*	D-H · · ·*A*	Symmetry code
	C16 – H16A· · ·O8	0.960(6)	2.772(5)	3.372(8)	121.3(4)	-1+*x*, *y*, -1+*z*
	C14 – H14C· · ·O3	0.960(5)	2.845(4)	3.608(6)	137.1(3)	-*x*, -*y*, -*z*
	C18 – H18· · ·O5	0.930(5)	2.564(4)	3.483(6)	169.9(3)	+*x*, +*y*, 1 +*z*
**2**	C26 – H26C· · ·O1	0.961(6)	2.966(3)	3.539(7)	119.5(3)	-*x*, -*y*, 1 -*z*
	C26 – H26C· · ·O9	0.961(6)	2.641(4)	3.528(8)	153.6(4)	-*x*, -*y*, 1 -*z*
	C27 – H27A· · ·O1	0.960(6)	2.910(3)	3.408(6)	113.4(3)	-*x*, -*y*, 1 -*z*
	C27 – H27A· · ·O9	0.960(6)	2.444(3)	3.361(7)	159.8(4)	-*x*, -*y*, 1 -*z*
	C26 – H26C· · ·O2	0.961(6)	2.664(3)	3.450(6)	139.4(2)	-1+*x*, +*y*, +*z*
	C27 – H27B· · ·O7	0.959(6)	2.774(3)	3.330(6)	117.7(3)	-1+*x*,-1+*y*, +*z*
	C52 – H52C· · ·O9	0.959(1)	2.803(3)	3.459(14)	126.4(8)	*x*, *y*, *z*
	C52 – H52F· · ·O9	1.480(15)	2.548(3)	3.459(14)	115.8(6)	*x*, *y*, *z*
	C9 – H9· · ·O5	0.930(4)	2.658(3)	3.506(5)	151.9(2)	1- *x*, 2- *y*, 1 – *z*
	C48 – H48A· · ·O4	0.960(6)	2.901(3)	3.605(7)	131,1(4)	2- *x*, 2- *y*, 1 – *z*
**3**	C12– H12· · ·O16	0.930(5)	2.575(4)	3.431(6)	153.2(3)	2- *x*, 2- *y*, 1 – *z*

	C34 – H34· · ·O13	0.930(4)	2.690(3)	3.538(5)	151.9(2)	1- *x*, 1- *y*, 2 – *z*
	C18 – H18· · ·O13	0.930(4)	2.631(4)	3.531(5)	163.1(2)	1- *x*, 1- *y*, 2 – *z*
	C37 – H37· · ·O8	0.929(4)	2.646(4)	3.471(5)	148.2(2)	-*x*, 1-*y*, 2 – *z*
	C51 – H51A· · ·O10	0.960(15)	2.612(3)	3.557(15)	168.2(9)	1+*x*, +*y*, +*z*
	C6 – H6· · ·O17	0.949(6)	2.604(6)	3.550(9)	174.2(4)	*x*, *y*, *z*
	C22 – H22· · ·O17	0.950(6)	2.415(5)	3.355(8)	169.8(4)	*x*, *y*, *z*
	C9– H9· · ·O16	0.951(6)	2.627(4)	3.518(7)	156.4(4)	*x*, *y*, 1 + *z*
	C25 – H25A· · ·O3	0.980(6)	2.880(5)	3.502(8)	122.2(4)	-*x*, 1- *y*, 1 - *z*
	C25 – H25B· · ·O4	0.979(6)	2.733(4)	3.524(8)	138.2(4)	-*x*, 1- *y*, 1 -
	C4 – H4· · ·O17	0.950(5)	2.826(7)	3.423(9)	121,9(3)	-*x*, 1- *y*, 1 - *z*
	C52 – H52B· · ·O13	0.980(9)	2.534(5)	3.299(10)	134.8(6)	*x*, 1+*y*, *z*
**4**	C13 – H13· · ·O13	0.949(7)	2.506(5)	3.269(8)	137.6(4)	*x*, 1+*y*, *z*
	C18 – H18· · ·O8	0.950(5)	2.467(4)	3.280(7)	143.5(3)	-*x*, 1- *y*, -*z*
	C43 – H43· · ·O8	0.950(7)	2.563(4)	3.416(7)	149.5(4)	-*x*, 1- *y*, -*z*
	C48– H48C· · ·O8	0.980(6)	2.682(4)	3.503(7)	141.6(4)	-*x*, 1- *y*, -*z*
	C23 – H23A· · ·O8	0.981(6)	2.658(4)	3.488(7)	142.5(4)	-*x*, 1- *y*, -*z*
	C31 – H31· · ·O5	0.951(7)	2.507(5)	3.441(9)	167.4(4)	1-*x*, - *y*, 1 –*z*
	C37 – H37· · ·O13	0.949(7)	2.785(4)	3.478(7)	130.5(4)	1-*x*,-1- *y*, 1 –*z*
	C50 – H50C· · ·O9	0.981(8)	2.885(4)	3.500(9)	121.6(4)	*x*, *y*, -1+z
	C16 – H16C· · ·O8	0.960(7)	2.814(7)	3.389(11)	119.3(5)	+1 + *x*, *y*, 1 + *z*
	C16 – H16A· · ·O2	0.959(7)	2.732(4)	3.541(8)	142,4(4)	*x*, *y*, 1 + *z*
	C18– H18· · ·O5	0.930(5)	2.594(5)	3.511(8)	168.5(4)	*x*, *y*, -1+ *z*
	C23 – H23A· · ·O4	0.962 (26)	2.687(4)	3.488(33)	141.0(17)	-1 + *x*, *y*, -1+
**5**	C26 – H26C· · ·O1	0.959(8)	2.910(4)	3.520(9)	122.63(5)	-1 + *x*, *y*, + *z*
	C26 – H26C· · ·O2	0.959(8)	2.687(4)	3.492(8)	141.8(5)	-*x*, 2- *y*, 1 - *z*
	C27 – H27A· · ·O9	0.959(7)	2.405(4)	3.339(9)	164.3(5)	*x*, 2- *y*, 1 - *z*
	C27 – H27B· · ·O7	0.959(7)	2.740(5)	3.346(8)	121.83(5)	-*x*, 1- *y*, 1 - *z*

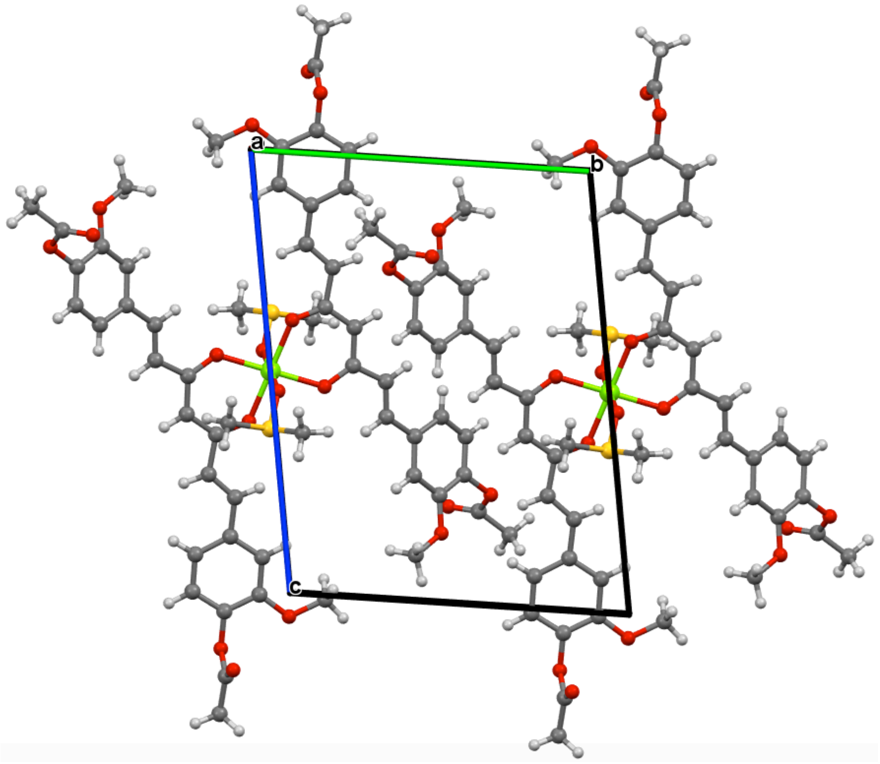

Figure S51. The packing diagram for **2** projected along the *a-axis*.

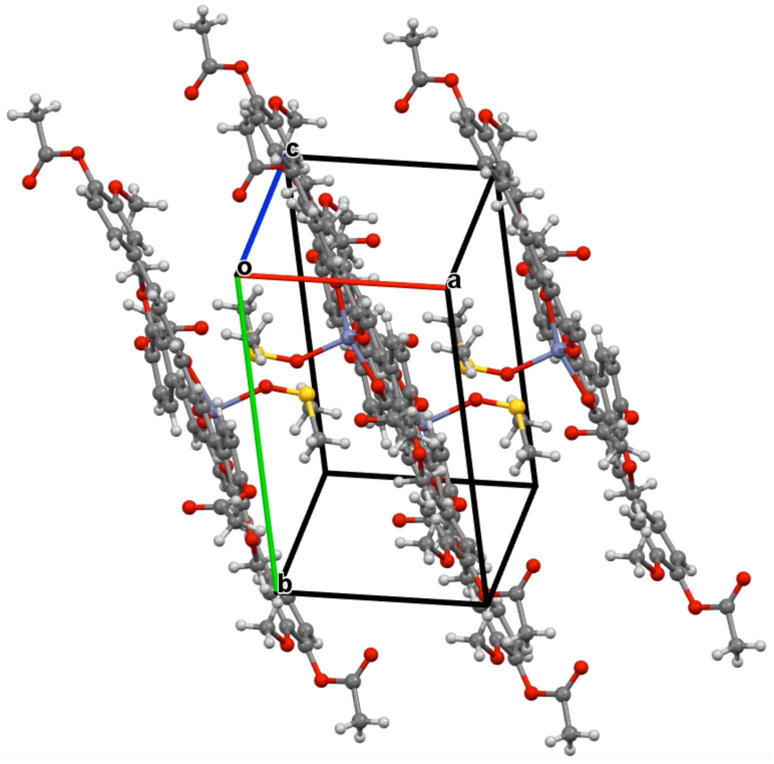

Figure S52. A perspective drawing of the packing arrangement for compound **3** along the *c-axis*.

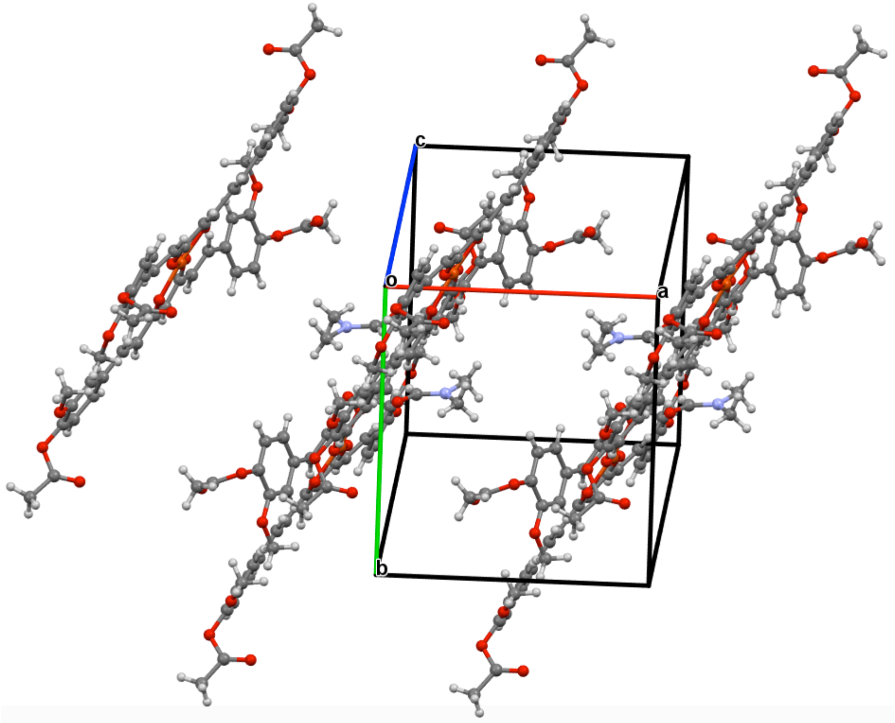

Figure S53. A perspective drawing of the packing arrangement for compound **4** along the *c-axis*.

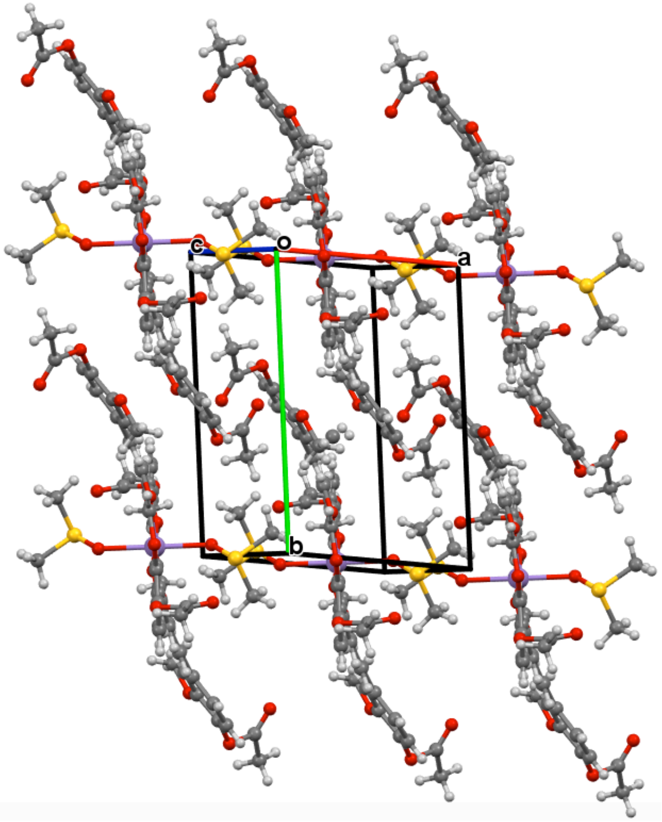

Figure S54. A perspective drawing of the packing arrangement for compound **5** along the *c-axis*.

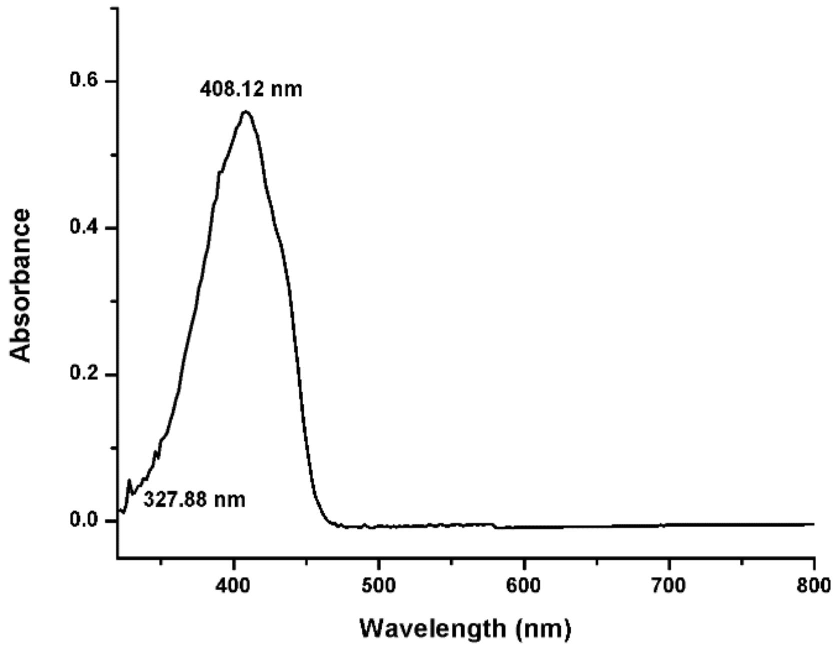

Figure S55. UV-Vis spectra of DAC.

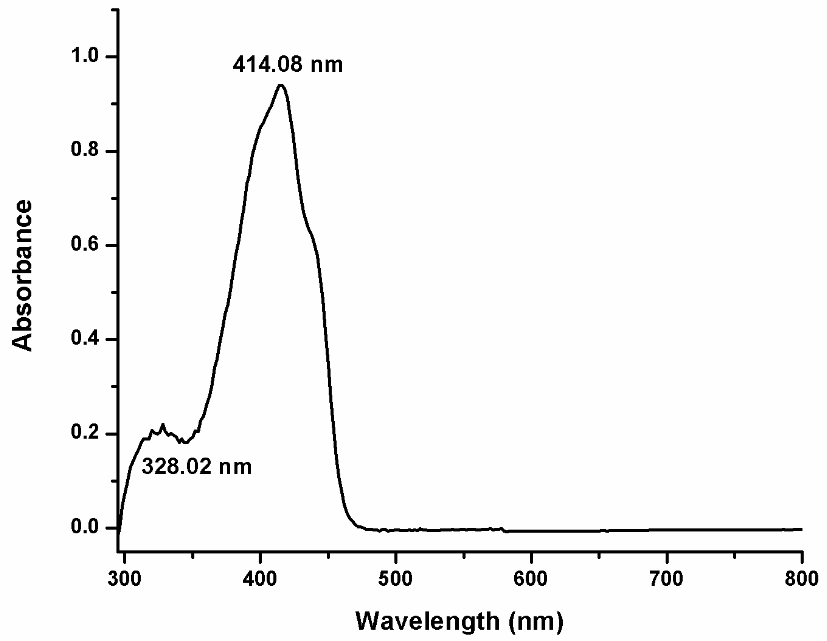

Figure S56. UV-Vis spectra of DAC-Mg.

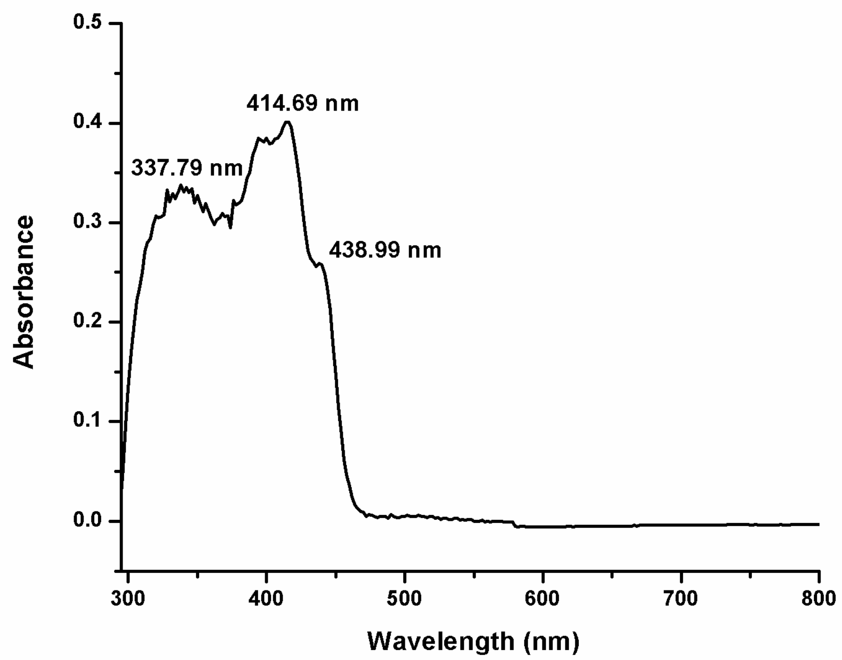

Figure S57. UV-Vis spectra of DAC-Zn.

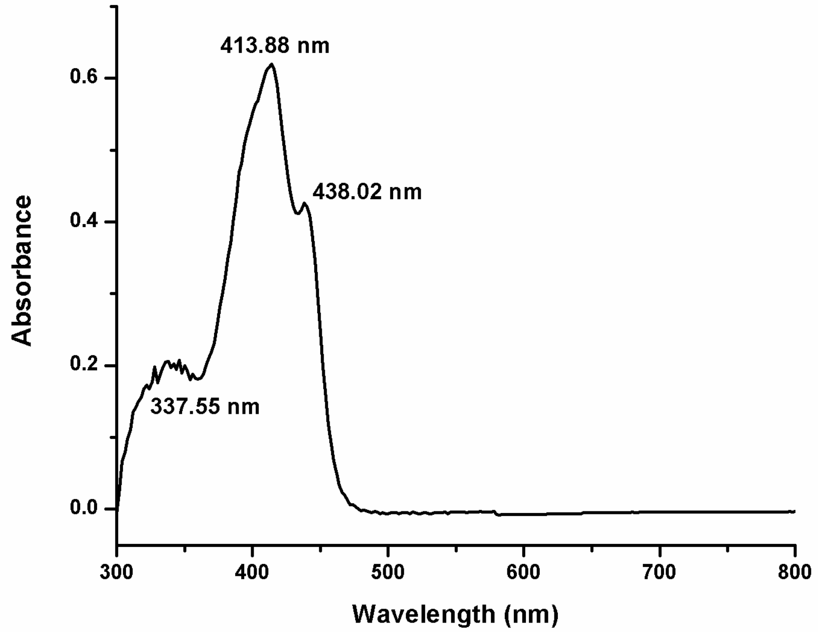

Figure S58. UV-Vis spectra of DAC-Cu.

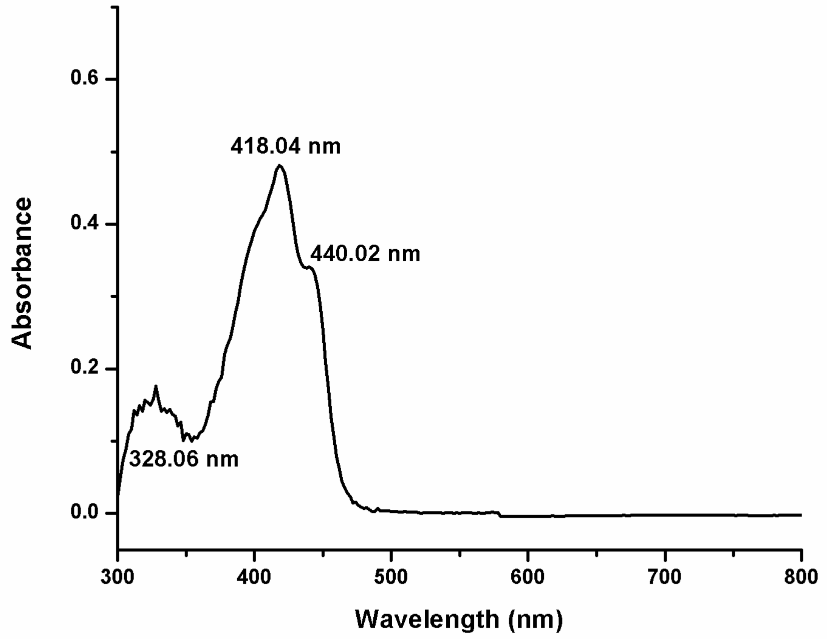

Figure S59. UV-Vis spectra of DAC-Mn.

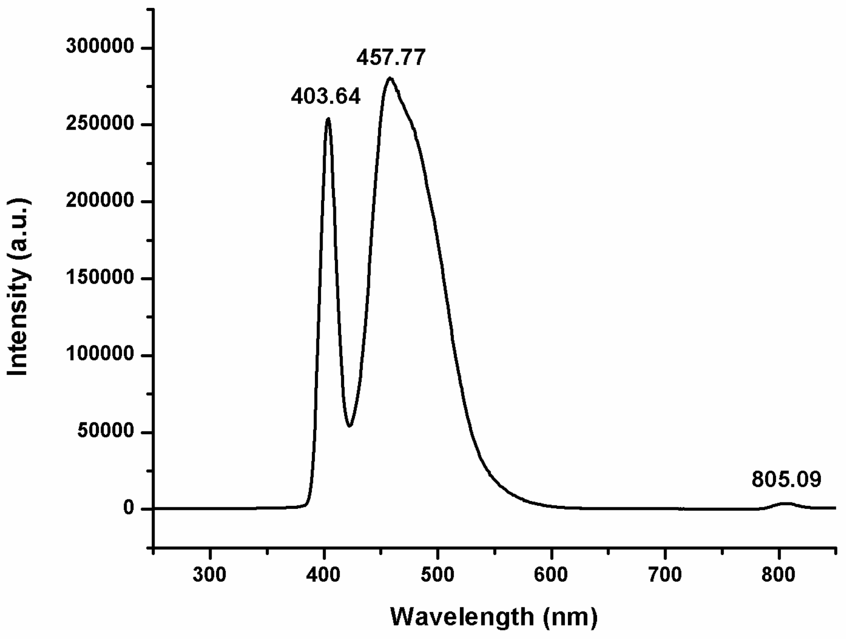

Figure S60. Fluorescence spectra of DAC.

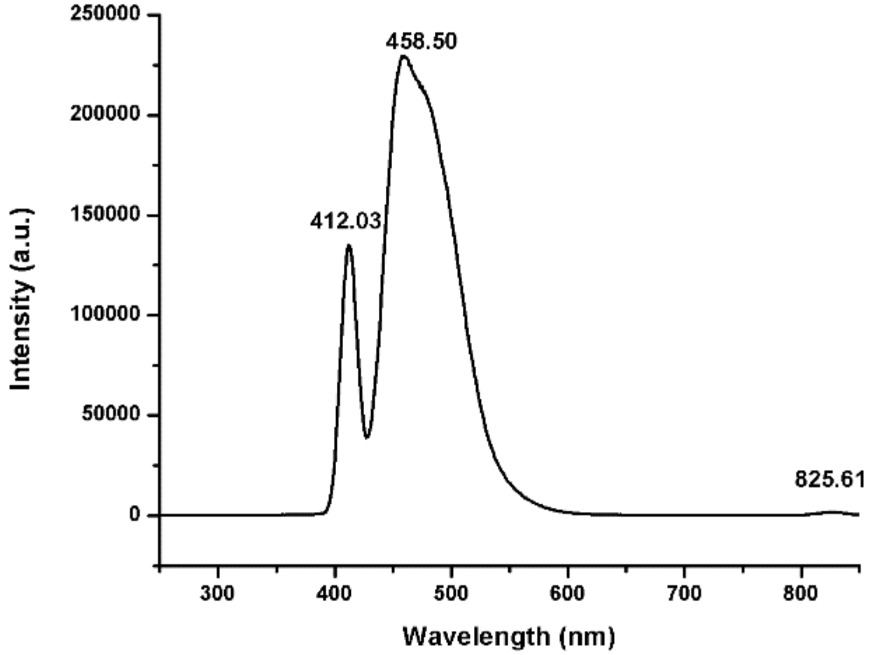

Figure S61. Fluorescence spectra of DAC-Mg.

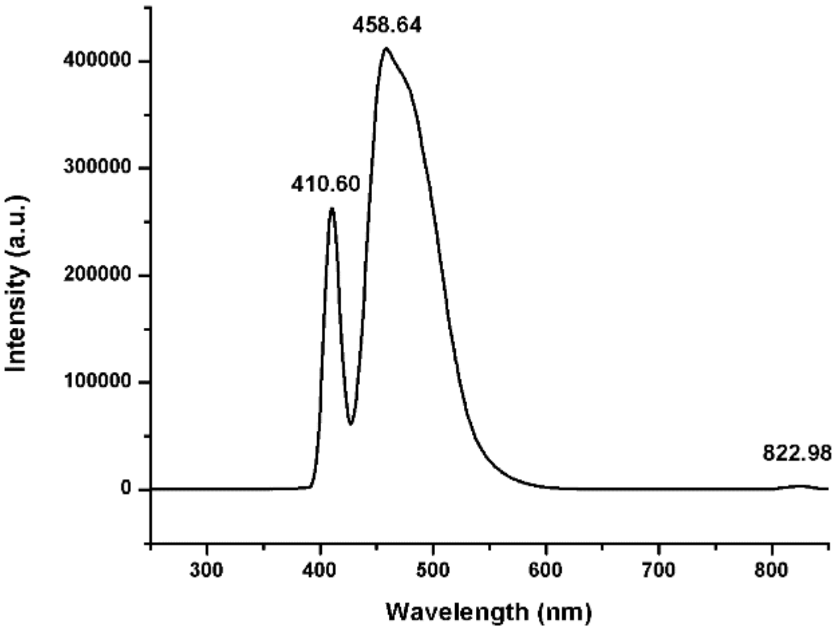

Figure S62. Fluorescence spectra of DAC-Zn.

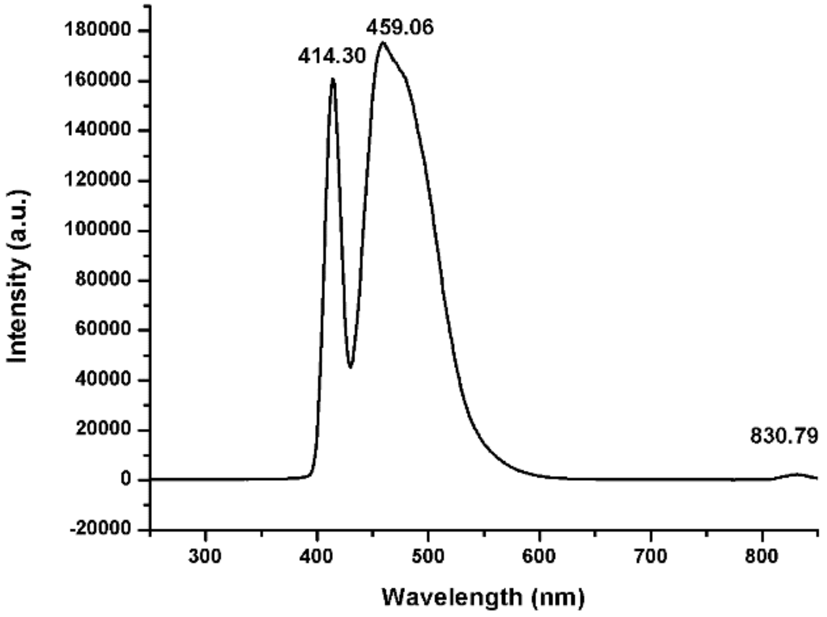

Figure S63. Fluorescence spectra of DAC-Cu.

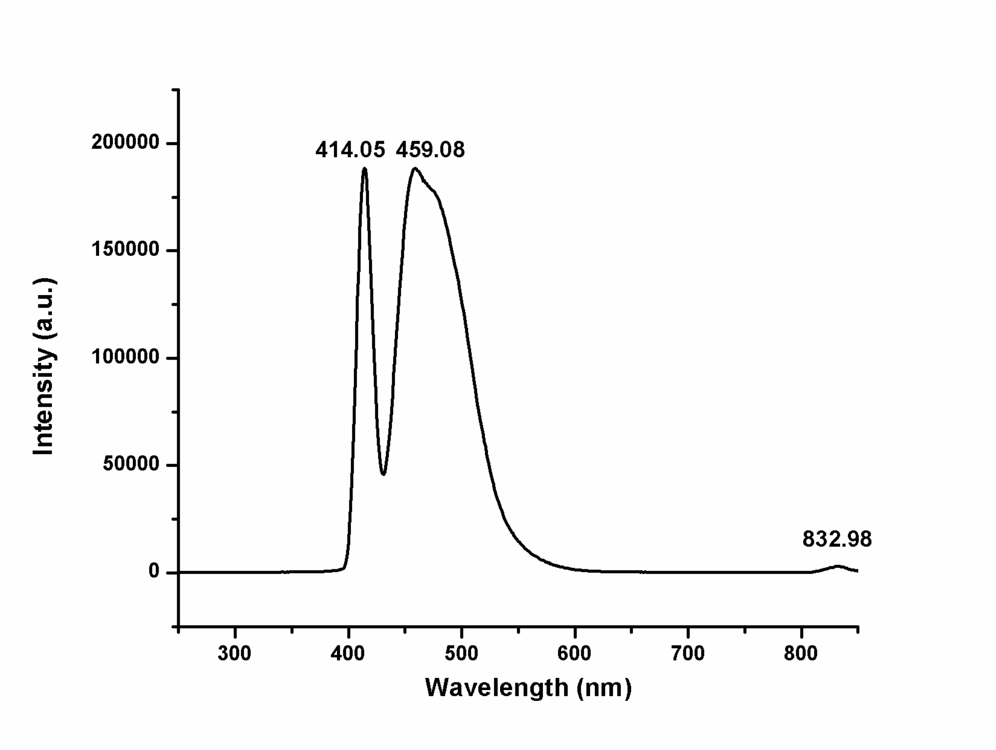

Figure S64. Fluorescence spectra of DAC-Mn.

METHODOLOGY OF CYTOTOXICITY ASSAY

Cell lines culture and culture medium

The compounds were screened *in vitro* against the following human cancer cell lines: HCT-15 (human colorectal adenocarcinoma), MCF-7 (human mammary adenocarcinoma), SKLU-1 (human lung adenocarcinoma). The cell lines were supplied by National Cancer Institute (USA). The human tumor cytotoxicity was determined using the protein-binding dye sulforhodamine B (SRB) in microculture assay to measure cell growth following the protocols established by the NCI.[30] The cell lines were cultured in RPMI-1640 medium supplemented with 10% fetal bovine serum, 2mM L-glutamine,10,000 IU penicillin G sodium, 10,000 μg/ml streptomycin sulfate (Corning) and 1% non-essential amino acids (Gibco). They were maintained at 37 °C in humidified atmosphere with 5% CO_2. The viability of the cells used in the experiments exceeded 95% as determined with trypan blue.

Cytotoxicity assay

The cells were removed from the tissue culture flasks by treatment with trypsin, and diluted with fresh media. Of these cell suspension, 100 μl /well of $5x10^4$ cell/ml (MCF7), $7.5x10^4$ cell/ml (SKLU-1) and $10x10^4$ cell/ml (HCT15) of cells suspension were seeded in 96 wells (micro-titer plates Costar) and incubated for 24 h to allow for cell adherence. The compounds were prepared from a stock concentration of 20 mM and solubilized in DMSO. After of incubation, 100 μl of each solution were added to each well at different concentrations (from 1.25 to 50 μM). After 48 h the incubation period, the adherent cell culture was fixed *in situ* by the addition of 50 μl of cold 50 % (wt/vol) in aqueous trichloroacetic acid (TCA) and incubated for 60 min at 4°C. The supernatant was discarded and the plates were washed three times with water and air-dried. Cultures fixed with TCA were stained for 30 min with 100 μl of a 0.4% SRB solution. Free SRB solution was then removed by washing with 1% aqueous acetic acid. The plates were the air-dried, and the bound dye was solubilized by the addition of 10 mM unbuffered Tris base (100 μl). The plates were placed on a shaker for 10 min and the absorption was determined at 515 nm using an ELISA plates reader (Bio-Tex Instruments).
